# EMT-ciliary signaling in quasi-mesenchymal-stem-like cells drives therapeutic resistance and is a druggable vulnerability in triple-negative breast cancer

**DOI:** 10.1038/s44321-025-00289-1

**Published:** 2025-08-26

**Authors:** Camille E Tessier, Jennifer Derrien, Aurore M M Dupuy, Thomas Pelé, Martin Moquet, Julie Roul, Elise Douillard, Camille El Harrif, Xavier Pinson, Matthieu Le Gallo, Florence Godey, Patrick Tas, Roselyne Viel, Eloïse Grasset, Claude Prigent, Éric Letouzé, Peggy Suzanne, Patrick Dallemagne, Mario Campone, Robert A Weinberg, Jacqueline A Lees, Philippe P Juin, Vincent J Guen

**Affiliations:** 1 Nantes Université, Inserm UMR 1307, CNRS UMR 6075, Université d’Angers, CRCI2NA, Équipe Labellisée LIGUE Contre le Cancer, Nantes, F-44000 France; 2 ICO René Gauducheau, Saint Herblain, France; 3 University Rennes, CNRS, Inserm, Biosit UAR3480 US_S 018, MRic Core Facility, Rennes, 35000 France; 4 Inserm UMR_S 1242, Oncogenesis Stress Signalling, University of Rennes, Rennes, France; 5 Centre de lutte contre le Cancer Eugène Marquis, Rennes, France; 6 Plateforme d’Histopathologie de Haute Précision (H2P2), Rennes, France; 7 CRBM, CNRS, Université de Montpellier, Montpellier, France; 8 Inserm, Nantes Université, CNRS, Université d’Angers, Nantes, CRCI2NA France; 9 Normandie Univ, UNICAEN, CERMN, Caen, 14000 France; 10 Department of Biology, Massachusetts Institute of Technology, Cambridge, MA USA; 11 MIT Department of Biology and the Whitehead Institute, Cambridge, MA USA; 12 Koch Institute for Integrative Cancer Research @ MIT, Cambridge, MA USA; 13 Nantes Université, CHU Nantes, CNRS, Inserm, BioCore, US16, SFR Bonamy, Nantes, France

**Keywords:** EMT, Primary cilia, Therapeutic Resistance, Triple-Negative Breast Cancer, Cancer, Stem Cells & Regenerative Medicine

## Abstract

Cancer therapeutic resistance is mediated, in part, by phenotypic heterogeneity and the plasticity of tumor cells, the latter being enabled by epithelial–mesenchymal transition (EMT). However, EMT in human cancer therapeutic response remains poorly understood. We developed patient-derived organoids (PDOs) from human triple-negative breast cancer (TNBC) and investigated their response to chemotherapy. We found that chemotherapy treatment kills the bulk of tumor cells in PDOs, but there is selective survival of malignant cells that had activated an EMT program, entered a quasi-mesenchymal, stem cell-like state and display primary cilia. We developed a family of small-molecule inhibitors of ciliogenesis and show that treatment with these inhibitors, or genetic ablation of primary cilia, is sufficient to suppress this chemoresistance via NFκB-induced cell death. We conclude that an EMT–ciliary signaling axis induces chemoresistance in quasi-mesenchymal ciliated stem-like cells to help tumors evade chemotherapy and represents a druggable vulnerability in human TNBC.

## Synopsis

Cells that activate an epithelial-mesenchymal transition (EMT) program to acquire a quasi-mesenchymal ciliated stem-like state were shown to mediate therapeutic resistance in human triple-negative breast cancer (TNBC).

- Quasi-mesenchymal ciliated stem-like cells represent a subset of late hybrid epithelial-mesenchymal cells.
- EMT-induced primary cilia mediate chemoresistance of quasi-mesenchymal ciliated stem-like cells.
- The small molecule Nao-3 represses primary ciliogenesis and induces the death of quasi-mesenchymal ciliated stem-like cells.

## Abstract (web summary)

Cells that activate an epithelial-mesenchymal transition (EMT) program to acquire a quasi-mesenchymal ciliated stem-like state were shown to mediate therapeutic resistance in human triple-negative breast cancer (TNBC).

## Graphical abstract

**Figure:**
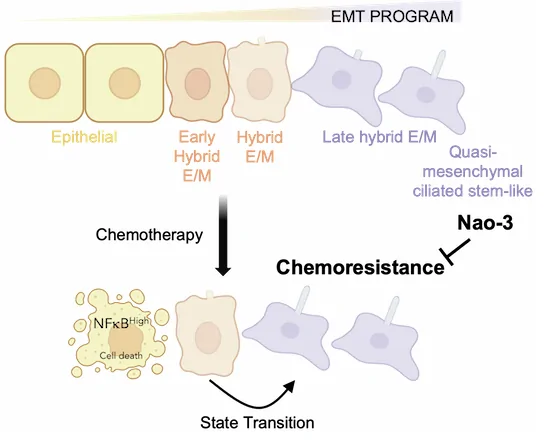

**Figure:**
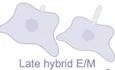

The paper explained **Problem**

Triple-negative breast cancer (TNBC) represents the most aggressive subtype of breast tumors. Most of the highly progressed forms of the disease display long-term resistance to therapy and therefore represent an unmet medical need. A better understanding of TNBC therapeutic response is urgently needed to define novel therapeutic strategies.

**Results**

Using patient-derived cancer samples, we reveal that quasi-mesenchymal ciliated stem-like cells drive therapeutic resistance in TNBC. We establish that primary cilia in these cells act downstream of EMT program activation to drive therapeutic resistance via regulation of NFκB signaling. We developed novel small-molecule inhibitors of primary ciliogenesis to selectively kill chemoresistant ciliated stem-like cells. We demonstrate that these drugs counteract EMT-induced TNBC therapeutic resistance.

**Impact**

Our findings reveal that an EMT–ciliary signaling axis drives therapeutic resistance of stem-like cells and is a druggable vulnerability in triple-negative breast cancer. We developed novel drugs to tackle this vulnerability.

## Introduction

Tumor cell heterogeneity and phenotypic plasticity contribute to chemotherapeutic resistance, which is responsible, in turn, for the death of most cancer patients (Bonnefoi et al, 2014; Gupta et al, 2009; Kim et al, 2018; Marine et al, 2020; Sharma et al, 2010). Epithelial–mesenchymal transition (EMT) is a cell biological program that drives tumor heterogeneity (Boyer et al, 1989; Dulbecco et al, 1981; Gupta et al, 2011; Mani et al, 2008; Thiery, 2002; Yang et al, 2004), enabling epithelial cancer cells to acquire an array of mesenchymal phenotypes (Bierie et al, 2017; Huang et al, 2013; Nieto et al, 2016; Pastushenko et al, 2018). Tumor cells that activate this cell plasticity program can transit in successive intermediate epithelial–mesenchymal (E/M) states before reaching a fully elongated mesenchymal morphology, an endpoint that is rarely seen in spontaneously arising tumors (Grasset et al, 2022; Gupta et al, 2019; Pastushenko and Blanpain, 2019). The distinct phenotypic intermediate E/M states arrayed between the two E-M extreme phenotypes are also referred to as quasi-mesenchymal states (Yang et al, 2020). Quasi-mesenchymal states encompass at least three distinct phenotypic states, including early hybrid, hybrid, and late hybrid, with different functional properties (Huang et al, 2013; Nieto et al, 2016; Pastushenko and Blanpain, 2019; Wilson et al, 2020; Yu et al, 2013).

The degree to which EMT is activated in the neoplastic cells of a tumor is thought to rely on its cell-of-origin, which influences tumor heterogeneity and impacts response to therapy (Gupta et al, 2019; Pommier et al, 2020). Early studies revealed that the EMT program endows carcinoma cells with chemotherapy resistance properties by influencing the expression of ABC drug efflux pumps (Del Vecchio et al, 2014; Saxena et al, 2011), antioxidant enzymes (Del Vecchio et al, 2014), or pro-apoptotic proteins (Wu et al, 2015), or by promoting DNA damage repair (Debaugnies et al, 2023). Other important studies showed that EMT can also trigger cancer cell resistance to targeted therapies, by promoting a shift in cell signaling dependencies (Tam et al, 2013; Zhang et al, 2012), as well as an elevated resistance to immunotherapy (Dongre et al, 2017), the latter achieved by orchestrating changes in tumor immune microenvironment (Dongre et al, 2021). Progress in understanding EMT-induced therapeutic resistance have been possible with the help of mouse tumor models and cancer cell lines in vitro (Shibue and Weinberg, 2017). Emerging studies indicate that neoplastic cells in distinct quasi-mesenchymal states operate as chemotherapy- and immune-checkpoint blockade (ICB)-resistant cells (Dongre et al, 2021; Luond et al, 2021; Sahoo et al, 2021). However, there is still limited understanding of which intermediate EMT states together with their related signaling circuitries mediate therapeutic resistance in human cancers.

Here, we used patient tumor biopsies and patient-derived cancer organoids (PDOs) to investigate the EMT states associated with therapeutic resistance in human triple-negative breast cancers (TNBC), and the role of primary cilia in this setting. The primary cilium is a microtubule-based organelle which is assembled as a solitary structure at the surface of a subset of cells in normal and neoplastic tissues, where it serves as a cell signaling hub (Guen and Prigent, 2020; Hilgendorf et al, 2024). Our past research revealed that EMT programs induce the formation of primary cilia to promote stemness of TNBC cancer cell lines by inducing the formation of primary cilia (Guen et al, 2017). Using a mouse carcinoma model, we further showed that EMT programs promote TNBC tumorigenesis by inducing primary ciliogenesis and ciliary signaling in tumor-initiating stem-like cells (Guen et al, 2017; Wilson et al, 2021). Importantly, the contribution of these cancer stem-like cells, and particularly their EMT–ciliary signaling axis, in the therapeutic response of human TNBC remains unknown.

In the present work we examine human TNBC, and show that cancer cells that have activated EMT and reside in a quasi-mesenchymal ciliated stem-like state represent a subset of late hybrid E/M cells that bear a primary cilium. We determine that quasi-mesenchymal ciliated stem-like cells display resistance to chemotherapy. We further establish that primary cilia promote EMT-induced chemotherapeutic resistance by repressing NFκB-dependent cell death in quasi-mesenchymal cells. TNBC represent the most aggressive group of human breast tumors. Most of the highly progressed forms of the disease display long-term resistance to therapy and therefore represent an unmet medical need despite recent progress with antibody-drug conjugates (Bardia et al, 2021). Our work reveals primary cilia as a novel vulnerability in TNBC and establishes that this is druggable.

## Results

### Quasi-mesenchymal stem-like cells assemble primary cilia in human TNBCs

**Figure 1:**
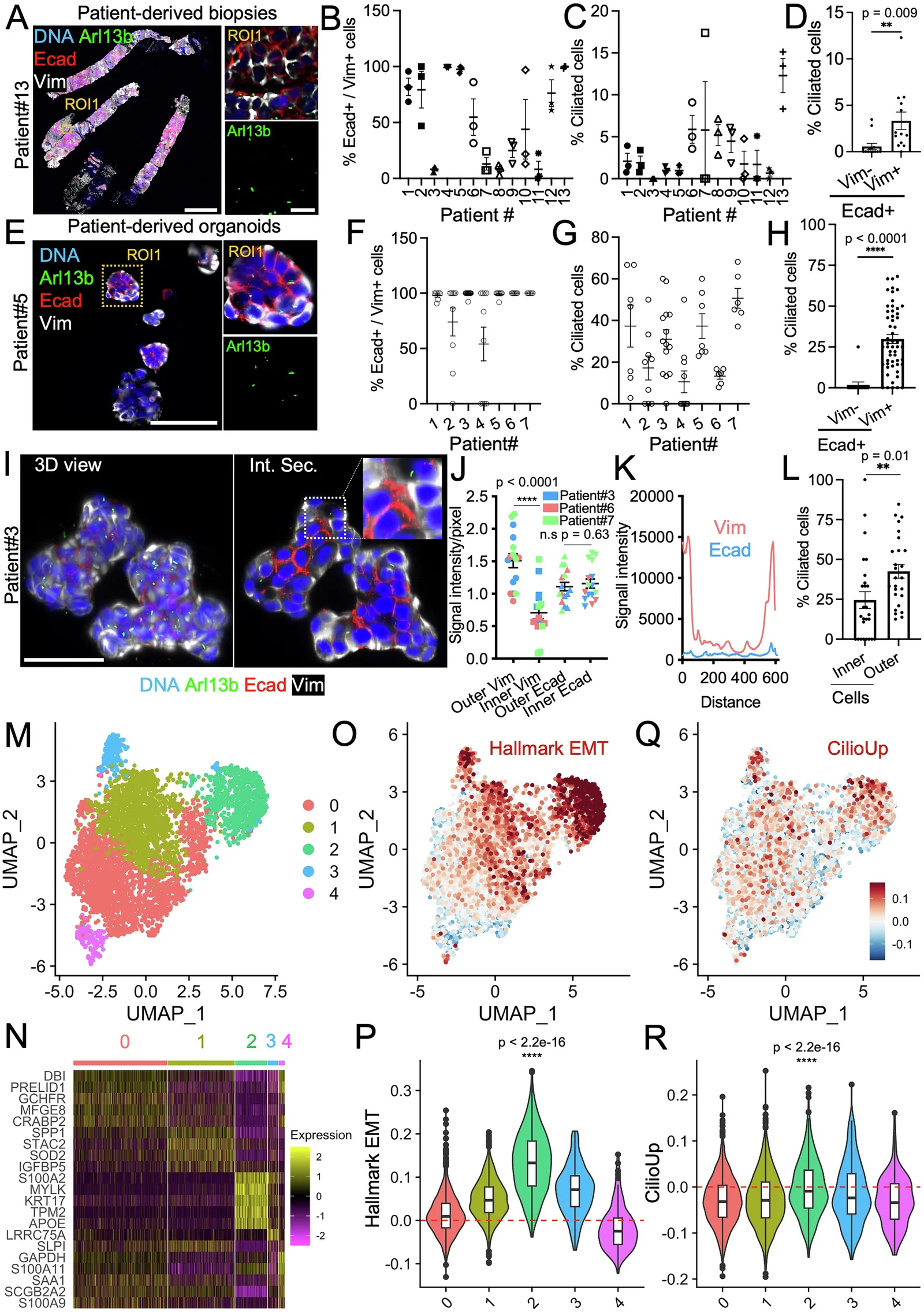
Quasi-mesenchymal cancer cells assemble primary cilia in human TNBCs. (**A**) Patient-derived tumor biopsies (PDB) were stained for the indicated proteins (*n* = 13, representative result for patient#13 is shown). Scale bar: 2 mm (insets: 3×). (**B**) The percentage of Ecad + /Vim+ cells was quantified in three distinct Ecad+ tumor region of interest (ROI) for each PDB (*n* = 13, mean ± s.e.m.). (**C**) The percentage of Ecad+ ciliated cells was quantified in the same ROIs (*n* = 13 mean ± s.e.m.). (**D**) The percentage of Ecad + /Vim+ vs Ecad + /Vim- ciliated cells was determined (*n* = 13, mean ± s.e.m.; Student’s *t* test: **P = 0.0090). (**E**) Patient-derived tumor organoids (PDOs) from distinct patient samples were stained for the indicated proteins (*n* = 7, representative result for patient #5). Scale bar: 100 μm (insets: 3×). (**F**, **G**) The percentage of Ecad + /Vim+ cells and the percentage of ciliated cells were quantified in optical sections per PDO in each patient sample (*n* ≥5 PDOs/patient sample, mean ± s.e.m.). (**H**) The percentage of Ecad + /Vim+ vs Ecad + /Vim− ciliated cells was determined. (*n* = 68 PDOs, six distinct patient samples, mean ± s.e.m.; Student’s *t* test: ****P < 0.0001). (**I**) Whole-mount immunofluorescence staining was conducted on PDOs for the indicated markers. 3-dimensional imaging of PDOs was performed. The 3D representation (3D view) as well as internal optical sections (int. sec.) are shown for representative PDOs for patient sample#3. Scale bar: 50 μm (inset: 2×). (**J**) Signal intensity for Ecad and Vim staining was measured across cells in individual PDOs for the three distinct patient samples, in cells residing within PDOs (inner) in comparison to cells residing at the periphery of PDOs (outer, *n* ≥5 PDOs/patient sample, mean ± s.e.m.; Student’s *t* test: n.s. *P* = 0.63; ****P < 0.0001). (**K**) Distribution of the Ecad and Vim signal across one representative PDO is shown. (**L**) Ciliation of cells residing within PDOs in comparison to cells on the outer part of PDOs was quantified (*n* = 26 PDOs from five distinct patient samples, mean ± s.e.m.; Student’s *t* test: **P = 0.0102). (**M**) Gene expression in cancer cells of PDOs was analyzed by scRNAseq (*n* = 4888 cells). UMAP illustrating transcriptional heterogeneity between cancer cells of PDOs. Each point represents a cell colored according to its cell cluster (0–4), with clustering performed at a resolution of 0.2. (**N**) Heatmap highlighting marker genes of each cluster. (**O**–**R**) UMAP and violin plots illustrating the expression of EMT and ciliogenesis signatures in the distinct cell clusters. Kruskal–Wallis test: ****P < 2.2e-16. Box plots show the median (center line), the 25th and 75th percentiles (lower and upper bounds of the box), and whiskers extending up to 1.5 times the interquartile range from the box limits. Data points beyond this range are considered outliers and are shown individually. Cluster 0 *n* = 2201, cluster 1 *n* = 1558, cluster 2, *n* = 747, cluster 3 *n* = 242, cluster 4 *n* = 140. Source data are available online for this figure.

To investigate epithelial–mesenchymal features and primary ciliogenesis in human TNBCs, we acquired patient-derived tumor biopsies (PDBs), from 13 individuals prior to any treatment, and also generated patient-derived organoids (PDOs) from 7 distinct patient samples (Fig. EV1A,B). We then stained sections of PDBs and entire PDOs, for the epithelial marker E-cadherin (Ecad), the mesenchymal marker Vimentin (Vim), the primary cilium marker (Arl13b) and the centrosome marker γTubulin (γTub), and analyzed the immunostainings by microscopy (Figs. 1A–H and EV1C,D). We found double-positive Ecad (Ecad + )/Vimentin (Vim + ) cancer cells residing in hybrid epithelial–mesenchymal (E/M) states in all patient samples (Fig. 1B,F). Importantly, the representation of Ecad + /Vim+ cancer cells varied between PDBs from distinct patients, and even between distinct neoplastic lesions of each tumor (Fig. 1B). Similarly, the representation of Ecad + /Vim+ cancer cells varied between PDOs from distinct patient samples as well as between PDOs arising from a single tumor specimen (Fig. 1F). The Arl13b staining revealed the presence of primary cilia on tumor cells in both PDB and PDO samples (Fig. 1A,E and EV1C,D). Their frequency also varied between and within single patient samples (Fig. 1C,G). Most importantly, we found that double-positive Ecad + /Vim+ cancer cells are significantly more ciliated than Ecad + /Vim- cells in PDBs (*P* = 0.009, Fig. 1D) and PDOs (*P* < 0.0001, Fig. 1H). These findings establish that human TNBCs display significant phenotypic heterogeneity at the level of both EMT state and primary ciliogenesis. Moreover, the propensity of cancer cells to form primary cilia is clearly coordinated with the acquisition of hybrid E/M phenotypes. However, only a subset of cells residing in hybrid E/M states form primary cilia.

**Figure EV1:**

Cancerous lesions in patient-derived biopsies and additional features of patient-derived cancer organoids. (**A**) Paraffin sections from patient-derived biopsies (PDBs) were stained with H&E (*n* = 13, a representative result for patient#13 is shown), Scale bars: 2 mm (low magnification) and 100 μm (high magnification). (**B**) The morphology of PDOs from distinct patient samples (pat. #1-7) was examined by brightfield microscopy. Scale bar: 100 μm. (**C**) Paraffin sections of tumors were stained for the indicated proteins (a representative image is shown). Scale bar: 15 μm, inset: 3x. (**D**) Patient-Derived Organoids (PDOs) were stained for the indicated proteins (a representative image is shown). Scale bar: 15 μm, inset: 3x. (**E**) Heatmap showing inferred CNVs in cells of PDOs from patient sample#3 with unsupervised hierarchical clustering. (**F**–**I**) UMAP and violin plots illustrating the expression of mammary stem cell and pluripotency signatures in the distinct cell clusters composing PDOs from patient sample#3. Kruskal–Wallis test: ****P < 2.2e-16. Box plots show the median (center line), the 25th and 75th percentiles (lower and upper bounds of the box), and whiskers extending up to 1.5 times the interquartile range from the box limits. Data points beyond this range are considered outliers and are shown individually. Cluster 0 *n* = 2201, cluster 1 n = 1558, cluster 2, *n* = 747, cluster 3 *n* = 242, cluster 4 *n* = 140.

To gain additional insight into the identity of hybrid E/M cells that form primary cilia, we conducted additional studies using PDOs that were exclusively composed of Ecad + /Vim+ cells, which expressed Vim over a range of levels, from very high to low but detectable (Fig. 1I,J). To begin, using whole-mount immunofluorescent staining and 3D-imaging studies of these structures, we found that the Ecad+ cells with significantly higher Vim levels were located on the outer layers of the PDOs (*P* < 0.0001, Fig. 1I–K), and they were significantly more likely to have primary cilia than the inner layer cells with lower levels of Vim (*P* = 0.01, Fig. 1I–L; Movie EV1). This finding supports the notion that the most mesenchymal-like state of the hybrid E/M cells are the ones that form primary cilia. We next conducted single-cell RNA sequencing (scRNAseq) analysis of tumor cells dissociated from PDOs and conducted unsupervised clustering based on differential gene expression (Fig. 1M). This revealed five different cell states (Cluster 0–4, Fig. 1M), of which cluster 2 displayed the most significant difference in gene expression (Fig. 1N; Dataset EV1). We used the inferCNV program to identify copy number alterations within our scRNAseq data, and found evidence for candidate genetic alterations in cancer cells (Fig. EV1E). The existence of shared CNVs between cells allowed us to identify subclones within the tumor cell population (Clone 1–4, Fig. EV1E). Constituent members of these subclones mapped to more than one of the cell state clusters (Fig. EV1E), supporting the notion that CNV-independent events contribute to the transcriptional heterogeneity of cell states.

To determine whether differential expression of EMT-associated genes contribute to transcriptional heterogeneity between cell states, we next assessed expression of an EMT signature within each of the identified clusters (Fig. 1O,P; Dataset EV2). This reveals clear differences in the levels between clusters; clusters 0 and 4 displayed the lowest levels, cluster 1 and 3 expressed intermediate levels, and cluster 2 expressed the highest level (Fig. 1O,P). Moreover, compared to the other clusters, cluster 2 showed significantly higher levels of gene sets associated with ciliogenesis (*P* < 2.2e-16, Fig. 1Q,R; Dataset EV2), and stem cell signatures (*P* < 2.2e-16, Fig. EV1F–I; Datasets EV2 and 3). Collectively, our findings establish that the subset of hybrid E/M cancer cells that reside in the most mesenchymal-like state, and display a stem-like phenotype, represents a subpopulation of late hybrid E/M stem-like cells that assemble primary cilia in human TNBCs. From here on, we refer to these cells as quasi-mesenchymal ciliated stem-like cells.

### Quasi-mesenchymal ciliated stem-like cells mediate chemoresistance

**Figure 2:**
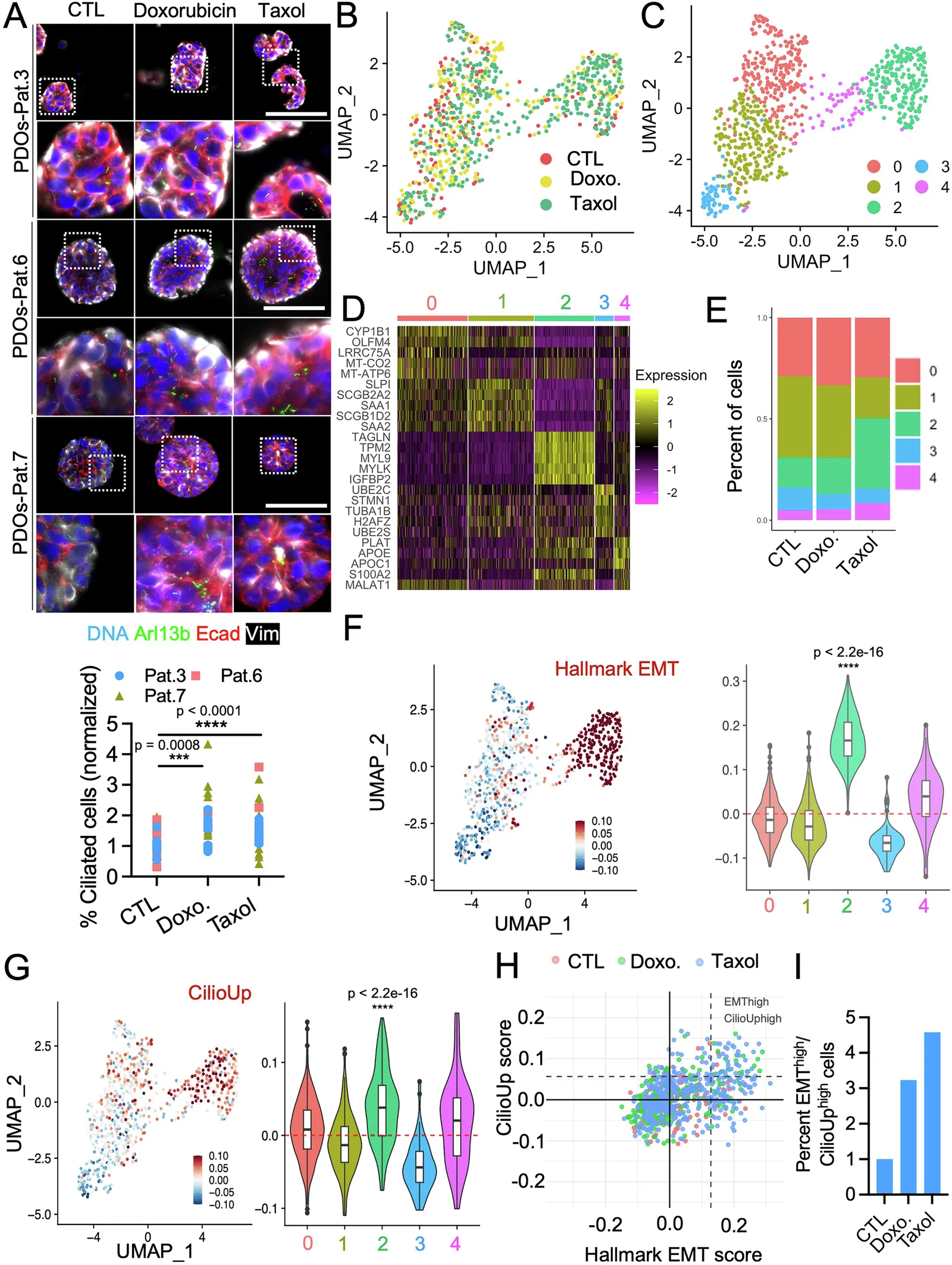
Quasi-mesenchymal ciliated cells mediate chemoresistance. (**A**) PDOs from three distinct patient (pat.) samples were stained for the indicated proteins. Scale bars: 100 μm (insets: 3×). The percentage of ciliated cells was quantified in optical sections in PDOs for each patient sample (*n* ≥33 PDOs/treatment condition from three distinct patient samples, mean ± s.e.m.; Student’s *t* test: ***P = 0.0008, ****P < 0.0001). (**B**) Gene expression in cancer cells of PDOs was analyzed by scRNAseq (*n* = 820 cells). UMAP plot integrating data from three samples (CTL = DMSO-treated, Doxo. = doxorubicin, Taxol). Each point represents a cell colored by sample of origin. (**C**) UMAP illustrating the different cell clusters identified by transcriptional heterogeneity. Each point represents a cell colored according to its cell cluster (0–4), with clustering performed at a resolution of 0.9. (**D**) Heatmap highlighting marker genes of each cluster. (**E**) Percentage of cells in each cluster per sample. (**F**, **G**) UMAP and violin plots illustrating the expression of EMT and ciliogenesis signatures in the distinct cell clusters. Kruskal–Wallis test: ****P < 2.2e-16. Box plots show the median (center line), the 25th and 75th percentiles (lower and upper bounds of the box), and whiskers extending up to 1.5 times the interquartile range from the box limits. Data points beyond this range are considered outliers and are shown individually. Cluster 0 *n* = 251, cluster 1 *n* = 234, cluster 2 *n* = 213, cluster 3 *n* = 66, cluster 4 *n* = 56. (**H**) Distribution of cells according to their expression of EMT and ciliogenesis programs. The expression thresholds (dashed line) used to define an EMT^high^/CilioUp^high^ population were calculated based on one standard deviation above the mean of expression of all cells. (**I**) The percentage of EMT^high^/CilioUp^high^ cells was determined for each sample and normalized to the control. Source data are available online for this figure.

To investigate the response of cancer cells residing in various EMT states to chemotherapy, we treated PDOs with doxorubicin or Taxol, two cytotoxic drugs that are in frequent use for clinical treatment of TNBC. We found that both chemotherapeutics reduced cancer cell viability in a dose-dependent manner (Fig. EV2A). Most importantly, a subset of the PDO cells displayed higher resistance to chemotherapy, and retained organoid-reconstituting capacity upon drug removal (Fig. EV2A,B). Using immunostaining, we found that these PDOs were significantly enriched for cells bearing primary cilia, compared to untreated controls (CTL, *P* = 0.0008: doxorubicin, *P* < 0.0001: Taxol, Fig. 2A). We also conducted scRNAseq analysis of cells dissociated from control or post-chemotherapy PDOs. Unsupervised clustering identified five distinct cell states (clusters 0–4, Fig. 2B–D; Dataset EV4). Cells of cluster 2 and 4 expressed high levels of *MYLK* and *TPM2* marker genes of the quasi-mesenchymal ciliated stem-like state (Figs. 2D and EV2C,D), and the highest levels of the EMT, ciliogenesis, and stem cell transcriptional programs (*P* < 2.2e-16, Figs. 2F,G and EV2E,F; Dataset EV5). These data indicate that these cells are in the quasi-mesenchymal ciliated stem-like state.

**Figure EV2:**

Impact of chemotherapy on PDOs and additional features of quasi-mesenchymal ciliated stem-like cells. (**A**) The dose-dependent impact of chemotherapy on cell viability in PDOs was tested for two distinct patient (pat.) samples (#3 and #7). Representative images of PDOs treated with DMSO (CTL) or with doxorubicin (doxo., 100 nM) and Taxol (10 nM) after 48 h of treatment are shown. The cell viability was measured at different concentrations and the IC50 is shown for each drug (mean ± s.e.m., representative analysis of 3 independent experiments). Scale bar: 100 μm. (**B**) The impact of drugs on the ability of chemoresistant cancer cells to reconstitute PDOs after drug removal at two intermediate drug concentrations is shown and measured at the indicated concentrations. Scale bar: 100 μm (*n* ≥213 PDOs/treatment condition, mean ± s.e.m.). (**C**–**F**) UMAP and violin plots illustrating the expression of *MYLK*, *TPM2*, mammary stem cell and pluripotency signatures in the distinct cell clusters from PDOs. Box plots show the median (center line), the 25th and 75th percentiles (lower and upper bounds of the box), and whiskers extending up to 1.5 times the interquartile range from the box limits. Data points beyond this range are considered outliers and are shown individually. Cluster 0 *n* = 251, cluster 1 *n* = 234, cluster 2 *n* = 213, cluster 3 *n* = 66, cluster 4 *n* = 56. (**G**, **H**) Expression levels of *MYLK* and *TPM2* was examined in cells from control and post-chemotherapy PDOs. The proportion of cells expressing high levels of *MYLK* and *TPM2* ( > 2) was determined for each sample and normalized to the control. *MYLK* > 2 CTL *n* = 12, Doxo. *n* = 42, Taxol *n* = 131; *TPM2* > 2 CTL *n* = 18, Doxo. *n* = 37, Taxol *n* = 128.

The percentage of cells from cluster 0 was similar between control and post-chemotherapy PDOs (Fig. 2E). In contrast, the post-chemotherapy PDOs showed reduction in the levels of cluster 1 and 3 cells and increases in the levels of cluster 2 and 4 cells (Fig. 2E), supporting the notion that quasi-mesenchymal ciliated stem-like cells are enriched in post-chemotherapy PDOs. In agreement with this finding, we detected that the proportion of cells expressing high levels of the quasi-mesenchymal ciliated stem-like cell markers MYLK and TPM2 are enriched in post-chemotherapy PDOs (Fig. EV2G,H). In addition, we examined the percentage of cells co-expressing high levels of EMT and ciliogenesis transcriptional programs in the distinct PDO samples. We found higher proportions of these cells in post-chemotherapy PDOs, compared to control PDOs (Fig. 2H,I). Altogether, our data establish that chemotherapy results in the selective survival of quasi-mesenchymal ciliated stem-like cells.

We wondered whether development of the quasi-mesenchymal ciliated stem-like cells in post-chemotherapy PDOs reflects selective survival of cells that acquired this state as a result of upregulation of EMT and ciliogenesis programs before treatment and/or as a response to the drugs. Using pseudotime analysis, we found a trajectory supporting the notion that cells of the quasi-mesenchymal ciliated stem-like cluster arise from cells of the non-quasi-mesenchymal ciliated stem-like clusters both in control and post-chemotherapy PDOs (Fig. EV3A–C). Distribution of single cells along the pseudotime trajectory significantly correlates with their progressive expression of EMT and ciliogenesis programs both in control and post-chemotherapy samples (Fig. EV3B,C). Interestingly, we found that the amplitude of the correlation is higher in the post-chemotherapy samples, compared to the control (Fig. EV3B,C). In addition, we found that some quasi-mesenchymal ciliated cells of cluster 2 which belong to post-chemotherapy PDOs express higher levels of EMT and ciliogenesis programs in comparison to the ones belonging to control PDOs (Fig. EV3B–D). Altogether, these data suggest that development of the quasi-mesenchymal ciliated stem-like cells in post-chemotherapy PDOs can both reflect selection of cells that acquired this state before treatment and of cells that acquired and progressed in this state as a response to the drugs.

**Figure EV3:**

Distribution of cells along the pseudotime trajectory and progressive expression of EMT and ciliogenesis programs. (**A**) UMAP combining data from 3 samples (PDOs from patient sample #3, CTL/Doxo./Taxol) colored by pseudotime value. Cells are ordered along an inferred trajectory; a black line connects all clusters. (**B**, **C**) Distribution of cells from distinct samples and clusters along the pseudotime trajectory and correlative analysis of the expression of EMT and CilioUp signatures. Wilcoxon test: Hallmark EMT score CTL *R* = 0.75 *P* < 2.2e-16, Doxo. *R* = 0.85 *P* < 2.2e-16, Taxol *R* = 0.86 *P* < 2.2e-16; CilioUp score CTL *R* = 0.42 *P* = 1.4e-07, Doxo. *R* = 0.56 *P* < 2.2e-16, Taxol *R* = 0.42 *P* < 2.2e-16. (**D**) Violin plots illustrating the expression of EMT and ciliogenesis transcriptional programs in individual cells of cluster 2 per treatment condition. CTL *n* = 21, Doxo. *n* = 48, Taxol *n* = 144.

### Primary cilia mediate chemoresistance of quasi-mesenchymal ciliated stem-like cells

**Figure 3:**
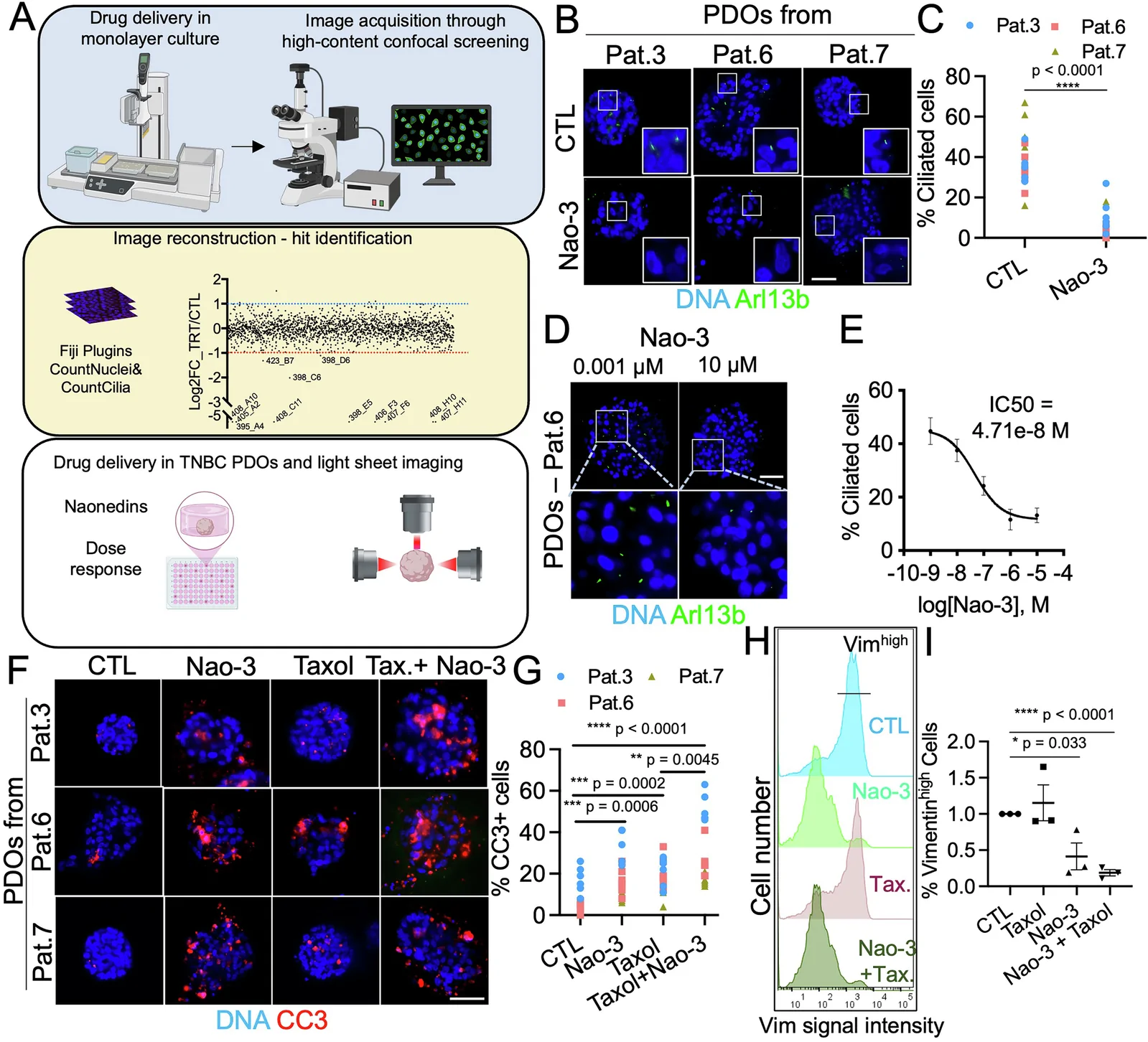
Naonedin-3 represses primary ciliogenesis and chemoresistance of quasi-mesenchymal ciliated stem-like cancer cells. (**A**) Schematic representation of the screening strategy for the identification of novel small-molecule inhibitors of primary ciliogenesis. (**B**) PDOs from three distinct patient (pat.) samples were treated with DMSO (CTL) or Naonedin-3 (Nao-3) and stained for Arl13b. Scale bar: 50 µm. (**C**) The percentage of ciliated cells was quantified. (*n* = 6 PDOs/patient sample/treatment condition; Student’s *t* test: ****P < 0.0001). (**D**) PDOs arising from patient (pat.) sample #6 were treated at the indicated concentration of Nao-3 and stained for the indicated markers. Representative images of PDOs are shown. Scale bar: 50 μm. (**E**) The percentage of ciliated cells was quantified in optical sections in PDOs and the IC_50_ is shown. (*n* ≥8 PDOs/concentration, mean ± s.e.m.). (**F**) PDOs were stained for the indicated proteins after the indicated treatments (Tax. = Taxol, Nao-3. = Naonedin-3). (**G**) The percentage of cleaved-caspase 3 (CC3)+ cells was measured in individual PDO for the distinct patient samples (*n* = 6 PDOs/patient sample/treatment condition; Student’s *t* test: CTL vs. Nao-3 ***P = 0.0006, CTL vs. Taxol ***P = 0.0002, CTL vs. Taxol + Nao-3 ****P < 0.0001, Taxol vs. Taxol + Nao-3 **P = 0.0045). Scale bar: 50 µm. (**H**, **I**) The percentage of Vim^high^ cells in PDOs after each treatment was determined by FACS using dissociated samples (*n* = 3, mean ± s.e.m.; Student’s *t* test: CTL vs. Nao-3 *P = 0.033, CTL vs. Nao-3 + Taxol ****P < 0.0001). Source data are available online for this figure.

**Figure EV4:**
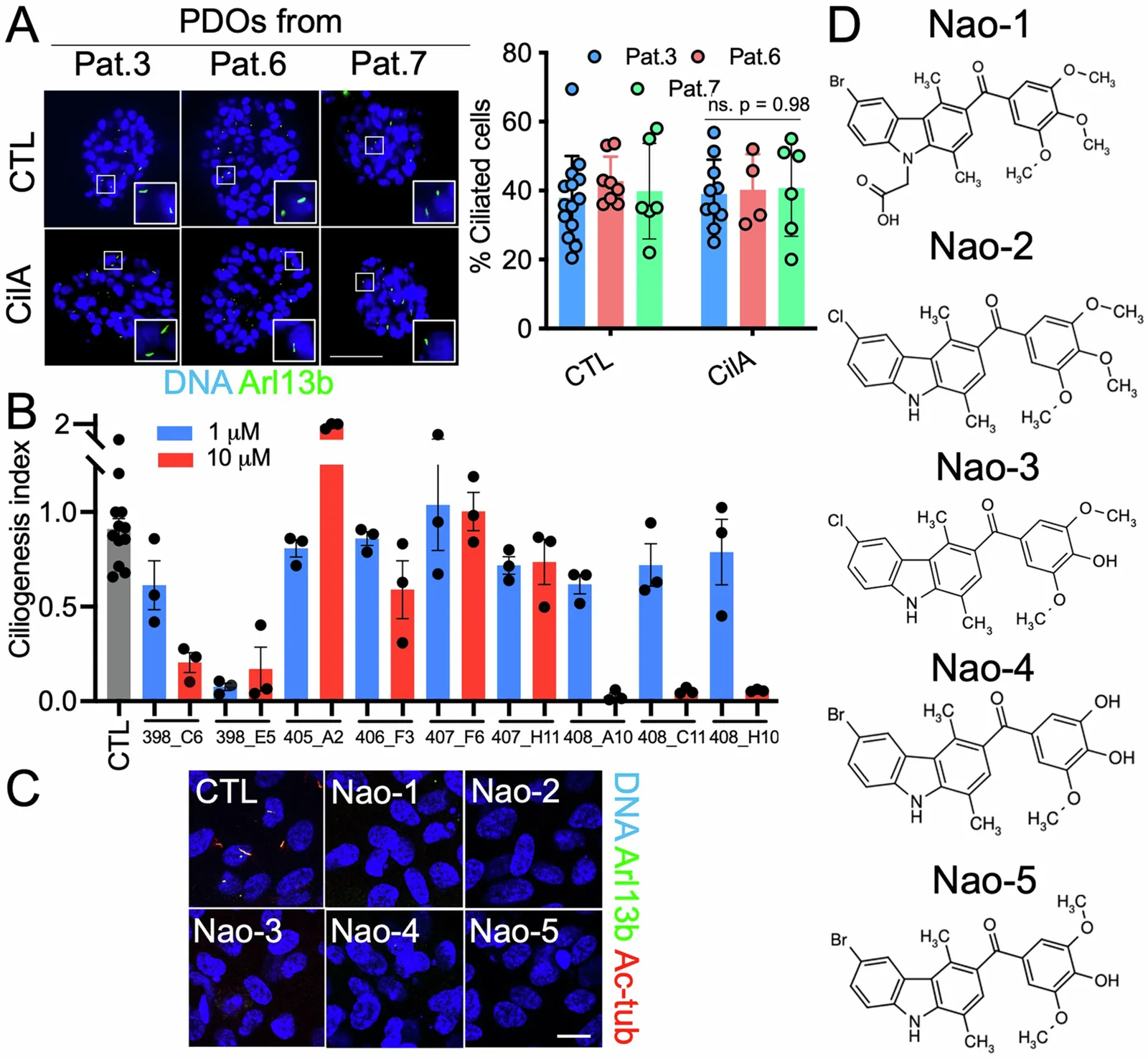
Identification of small-molecule inhibitors of primary ciliogenesis. (**A**) PDOs from distinct patient (pat.) samples were treated with DMSO (CTL) or Ciliobrevin A (CilA, 100 µM, 24 h) and stained for Arl13b. Scale bar: 50 µm. The percentage of ciliated cells was quantified in distinct PDOs for each patient sample (*n* ≥4 PDOs/patient sample/treatment condition, means ± s.e.m.; Student’s *t* test: CTL vs. CilA n.s. *P* = 0.98). (**B**) A secondary screen with small molecules identified as candidate ciliogenesis inhibitors in a primary screen (Fig. 3A) at two distinct concentrations (1 and 10 µM) was conducted. The ciliogenesis index was determined 24 h after each treatment. Results are relative to CTL, DMSO-treated cells (*n* = 3, mean ± s.e.m.). (**C**) The impact of the most potent inhibitors on ciliogenesis is shown (10 μM, Nao-1 = 398_C6; Nao-2 = 398-E5; Nao-3 = 408-A10; Nao-4 = 408_C11; Nao-5 = 408_H10). Scale bar: 15 µm. (**D**) Chemical structures of Naonedins.

We next wondered whether the primary cilia were actively promoting chemoresistance of quasi-mesenchymal stem-like cells in our PDOs versus simply being a hallmark of these cells. Human TNBC PDOs are difficult to maintain in long-term culture, precluding the use of genetic strategies to ablate primary cilia. Thus, as an alternative, we pursued a pharmacological approach. First, we tested the impact of ciliobrevin A (CilA), an inhibitor of the AAA+ ATPase motor dyneins that has been reported to inhibit ciliogenesis in monolayers of cells in culture (Firestone et al, 2012; Guen et al, 2017). Unfortunately, CilA treatment had no detectable effect on ciliogenesis in our PDOs (*P* = 0.98, Fig. EV4A), and no other specific small-molecule inhibitor repressing ciliogenesis in organoids had been reported. Given these findings, we decided to search for novel small molecules that inhibit primary ciliogenesis both in monolayer culture and in organoids using a two-step approach (Fig. 3A). We developed a microscopy-based high-content screening (HCS) assay for the identification of drugs that repress primary ciliogenesis in monolayer culture of human cells (RPE1), which are easily propagated and highly ciliated in vitro, and subsequently tested positive hits in three-dimensional culture conditions using our TNBC PDOs (Fig. 3A). We treated cells in monolayer culture with a library of 3271 compounds from the French National Chemical Library, fixed and stained for DNA and primary cilia markers (Arl13b and Acetylated tubulin), and scored the results via HCS (Fig. 3A). We established a computer code to enable automatic detection and segmentation of nuclei and cilia in the HCS images, allowing rapid assessment of drug impact on cell viability and ciliogenesis (Fig. 3A). This primary screen identified 12 candidate ciliogenesis inhibitors (Fig. 3A). We confirmed the capability of five drugs to repress primary ciliogenesis in monolayer culture (Fig. EV4B,C). Notably, five of these drugs belonged to the same chemical family, which we named Naonedin (Fig. EV4D). We then tested these drugs for their ability to repress primary ciliogenesis in PDOs, and found that Nao-3 significantly repressed cilium assembly in multiple patient samples analyzed (*P* < 0.0001, Fig. 3B,C), and in a dose-dependent manner (IC_50_ = 4.71e-8 M, Fig. 3D,E).

We then asked whether Nao-3 affected the chemoresistance phenotypes of our PDOs. For this, we treated patient-derived samples composed of cells in distinct hybrid E/M states with vehicle control, Nao-3 alone, Taxol alone or a combination of Nao-3 and Taxol, and quantified cell death by immunostaining for cleaved-caspase 3 after 72 h of treatment (Fig. 3F,G). We found that Nao-3 alone induced a modest but significant increase in the death of cancer cells that was associated with caspase activation (*P* = 0.0006, Fig. 3F,G). Most importantly, we observed significantly higher levels of cell death when Nao-3 and Taxol were combined (*P* < 0.0001, Fig. 3F,G). Altogether, our data show that we have successfully identified a novel family of small molecules that inhibit primary ciliogenesis, and effectively suppress the emergence of chemoresistance in patient-derived samples.

We further extended our analyses to determine whether Nao-3 specifically impairs the viability of the subset of quasi-mesenchymal cells in PDOs. We again used vehicle control, Nao-3 alone, Taxol alone or a combination of Nao-3 and Taxol to treat our PDOs composed of cells expressing Vim over a range of levels. We dissociated the PDOs post-treatment, stained for Vim and used fluorescence-activated cell sorting (FACS) to assess the proportion of Vim^high^ cells in each sample. In agreement with our imaging studies, FACS detected a population of Vim^high^ cells, as well as cells expressing lower levels of Vim, including below the detection limit by FACS for some cells (Fig. 3H). Importantly, we found that treatment with either Nao-3 alone, or Nao-3 plus Taxol caused a significant reduction in the levels of Vim^high^ cells, compared to either vehicle control or Taxol treatment (Nao-3: *P* ≤0.05, Nao-3+Taxol: *P* < 0.0001, Fig. 3H,I). Altogether, our findings support the notion that primary ciliogenesis promotes the development of the quasi-mesenchymal ciliated stem-like cells and their survival in response to chemotherapeutic treatment, and thus represent a vulnerability for TNBC.

### EMT-driven cell plasticity enables formation of primary cilia in quasi-mesenchymal cells to mediate chemoresistance

**Figure 4:**
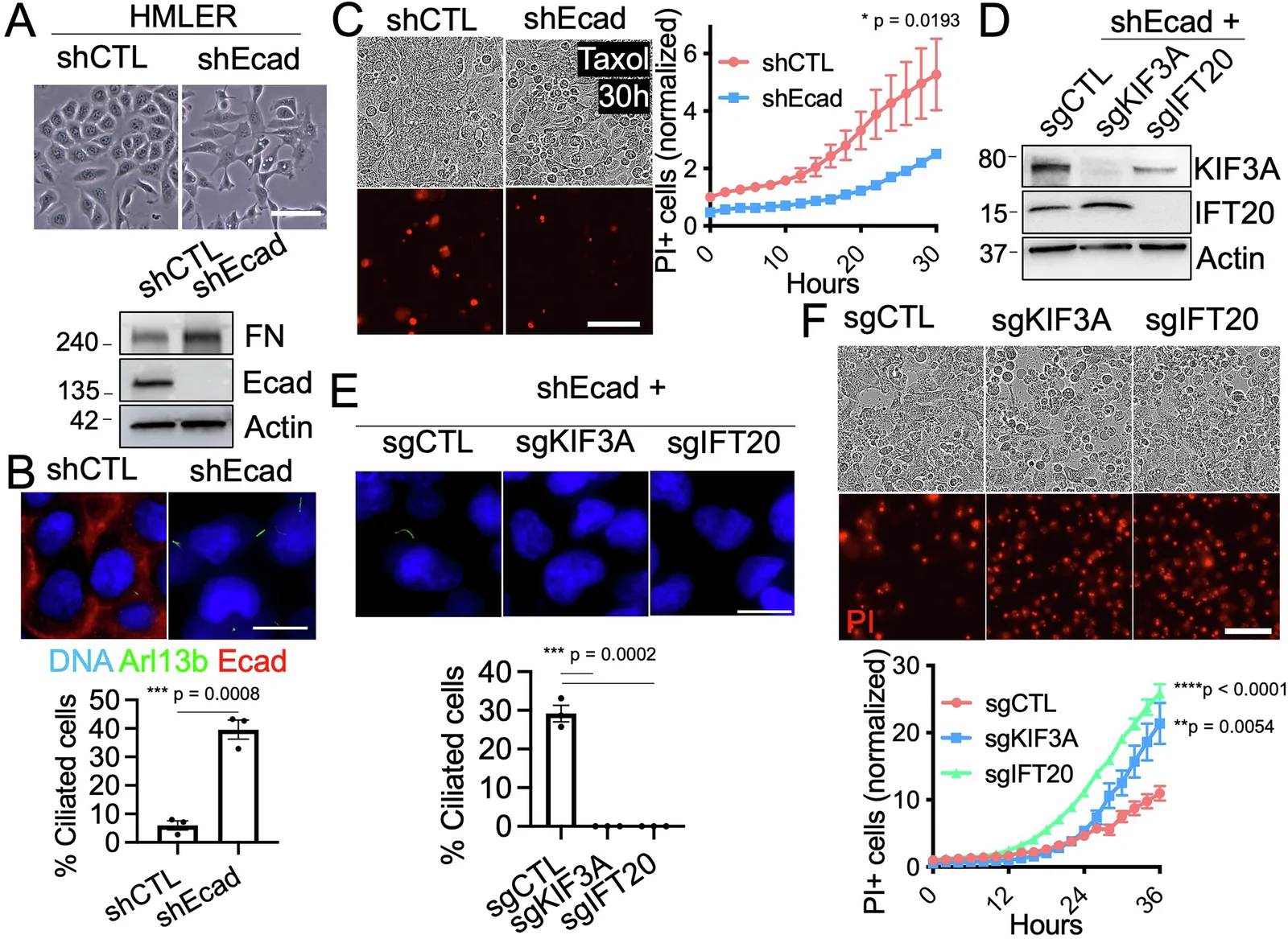
EMT-driven cell plasticity enables formation of primary cilia in quasi-mesenchymal cells to mediate chemoresistance. (**A**) Morphology and western blot analysis of EMT markers in E-like (shCTL) and M-like (shECAD) HMLER cells. Scale bar: 100 µm. (**B**) Cells were stained for the indicated proteins to determine the percentage of ciliated cells (*n* = 3, mean ± s.e.m.; Student’s *t* test: ***P = 0.0008). Scale bar: 15 µm. (**C**) The sensitivity of shCTL and shEcad HMLER cells to Taxol was determined by quantifying incorporation of propidium iodide (PI) in cancer cells in response to Taxol (1 µM) over time (30 h) (*n* = 3, mean ± s.e.m.; Student’s *t* test: shCTL vs. shEcad 30 h *P = 0.0193). Results are normalized to the HMLER shCTL. Scale bar: 100 µm. (**D**) *KIF3A* and *IFT20* knockouts were validated by western blot of extracts for the indicated HMLER variants. (**E**, **F**) The impact on ciliogenesis and on Taxol sensitivity was assessed as described in (**B**, **C**). Scale bars: 15 µm (**E**) and 100 µm (**F**). *n* = 3, mean ± s.e.m.; Student’s *t* test (**E**): ***P = 0.0002. (**F**) sgCTL vs. sgKIF3A **P = 0.0054, sgCTL vs. sgIFT20 ****P < 0.0001). Source data are available online for this figure.

To determine whether EMT-induced cellular plasticity enables the formation of primary cilia, and whether primary cilia promote chemoresistance of quasi-mesenchymal cells in other contexts, we used experimentally transformed human mammary epithelial cells (HMLER). The majority of HMLER tumor cells display epithelial characteristics when propagated in vitro, but EMT can be induced experimentally in these cells by expression of shEcad, enabling the cells to acquire a quasi-mesenchymal phenotype (Guen et al, 2017; Wilson et al, 2021). We have previously generated control epithelial (shCTL) and quasi-mesenchymal (shEcad) HMLER variants (Guen et al, 2017; Wilson et al, 2021). Thus, we studied the phenotypic responses of both variants. We confirmed that most of the shCTL cells display epithelial characteristics, while the shEcad cells acquire quasi-mesenchymal characteristics upon Ecad silencing (Fig. 4A). Of note, shEcad cells resided in a more advanced quasi-mesenchymal state along the EMT spectrum in comparison to the quasi-mesenchymal ciliated stem-like cells of PDOs. However, we demonstrated that quasi-mesenchymal HMLER shEcad cells also display increased levels of primary cilia, as by Arl13b immunostaining (*P* = 0.0008, Fig. 4B), enabling analysis of the role of primary cilia in cells that acquire a quasi-mesenchymal phenotype upon EMT in this model. We next compared the ability of the epithelial shCTL HMLER cells, versus the quasi-mesenchymal ciliated shEcad HMLER variants, to develop resistance to Taxol by monitoring cell death through propidium iodide staining (Fig. 4C). We found that the quasi-mesenchymal, primary cilia-bearing, HMLER cells were significantly more resistant to Taxol-induced cell death than the control, epithelial HMLER cells (*P* = 0.0193, Fig. 4C; Movie EV2). Thus, cellular plasticity enabling cells to transit from the epithelial to a quasi-mesenchymal state promotes primary ciliogenesis and chemoresistance.

To determine whether primary cilia are required for the chemoresistance of quasi-mesenchymal cells, we used HMLER shEcad variants in which we had knocked out *KIF3A* or *IFT20*, two genes that are essential for ciliogenesis. In both cases, the loss of KIF3A and IFT20 proteins and consequent inhibition of ciliogenesis (*P* = 0.0002, Fig. 4D,E) significantly increased the sensitivity of the HMLER shEcad variants to Taxol-induced death (sgKIF3A *P* = 0.0054 and sgIFT20 *P* < 0.0001, Fig. 4F; Movie EV3). Thus, inhibition of primary ciliogenesis, through either pharmacologic or genetic methods, serves to sensitize quasi-mesenchymal cancer cells to chemotherapy. Together, these data establish that EMT-driven cancer cell plasticity enables primary ciliogenesis and chemoresistance in quasi-mesenchymal cells, and that acquired chemotherapy resistance is dependent on primary ciliogenesis.

### Primary cilia mediate chemoresistance by repressing NFκB-dependent cell death in quasi-mesenchymal ciliated stem-like cells

**Figure 5:**
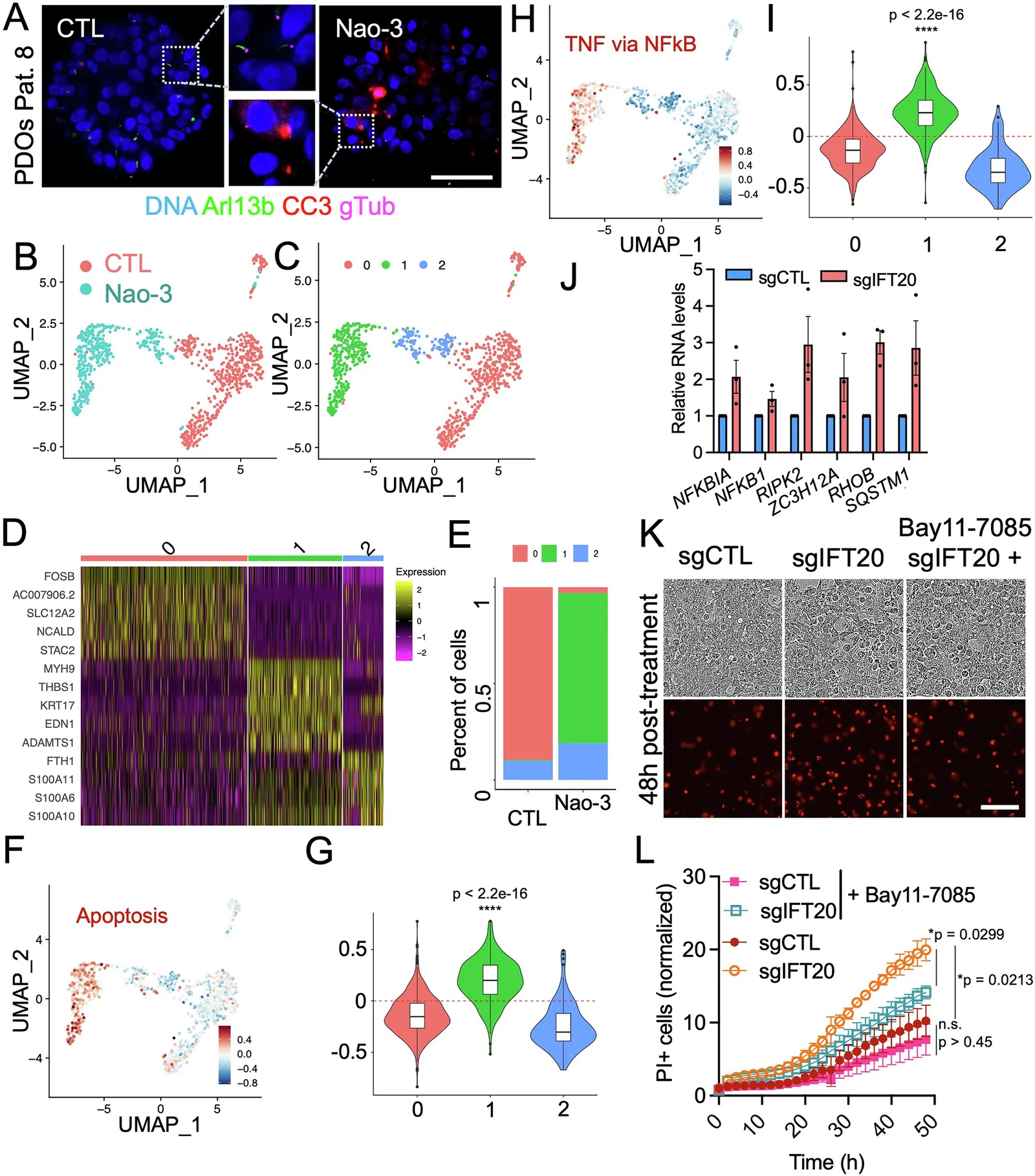
Primary cilia mediate chemoresistance by repressing NFκB-mediated cell death in quasi-mesenchymal ciliated cancer cells. (**A**) PDOs were stained for the indicated proteins after treatment with DMSO (CTL) or Naonedin-3 (Nao-3). Scale bar: 100 µm. (**B**) Gene expression in cancer cells of PDOs was analyzed by scRNAseq (*n* = 902 cells). UMAP plot integrating data from the distinct samples (CTL = DMSO-treated, Nao-3. = Naonedin-3). Each point represents a cell colored by the sample of origin. (**C**) UMAP illustrating the different cell clusters identified by transcriptional heterogeneity. Each point represents a cell colored according to its cell cluster (0–2), with clustering performed at a resolution of 0.1. (**D**) Heatmap highlighting marker genes of each cluster. (**E**) Percentage of cells in each cluster per sample. (**F**–**I**) UMAP and violin plots illustrating the expression of apoptosis and NFκB signatures in the distinct cell clusters. Kruskal–Wallis tests: ****P < 2.2e-16. Box plots show the median (center line), the 25th and 75th percentiles (lower and upper bounds of the box), and whiskers extending up to 1.5 times the interquartile range from the box limits. Data points beyond this range are considered outliers and are shown individually. Cluster 0 *n* = 499, cluster 1 *n* = 281, cluster 2 *n* = 122. (**J**) Relative levels of the indicated gene transcripts in HMLER shECAD sgCTL or sgIFT20 variants treated with Taxol were determined by real-time qPCR analysis. *n* = 3, mean ± s.e.m. (**K**, **L**) The sensitivity of HMLER shEcad sgCTL or sgIFT20 variants to Taxol alone or in combination with Bay11-7085 was determined by quantifying incorporation of propidium iodide (PI) in cancer cells over time (48 h). Scale bar: 100 µm. *n* = 3, mean ± s.e.m., results are normalized to the HMLER shEcad sgCTL after Taxol treatment. Student’s *t* test: sgCTL vs. sgIFT20 *P = 0.0213, sgIFT20 vs. sgIFT20 + Bay11-7085 *P = 0.0299. sgCTL vs. sgCTL + Bay11-7085 n.s. *P* > 0.45. Source data are available online for this figure.

**Figure EV5:**
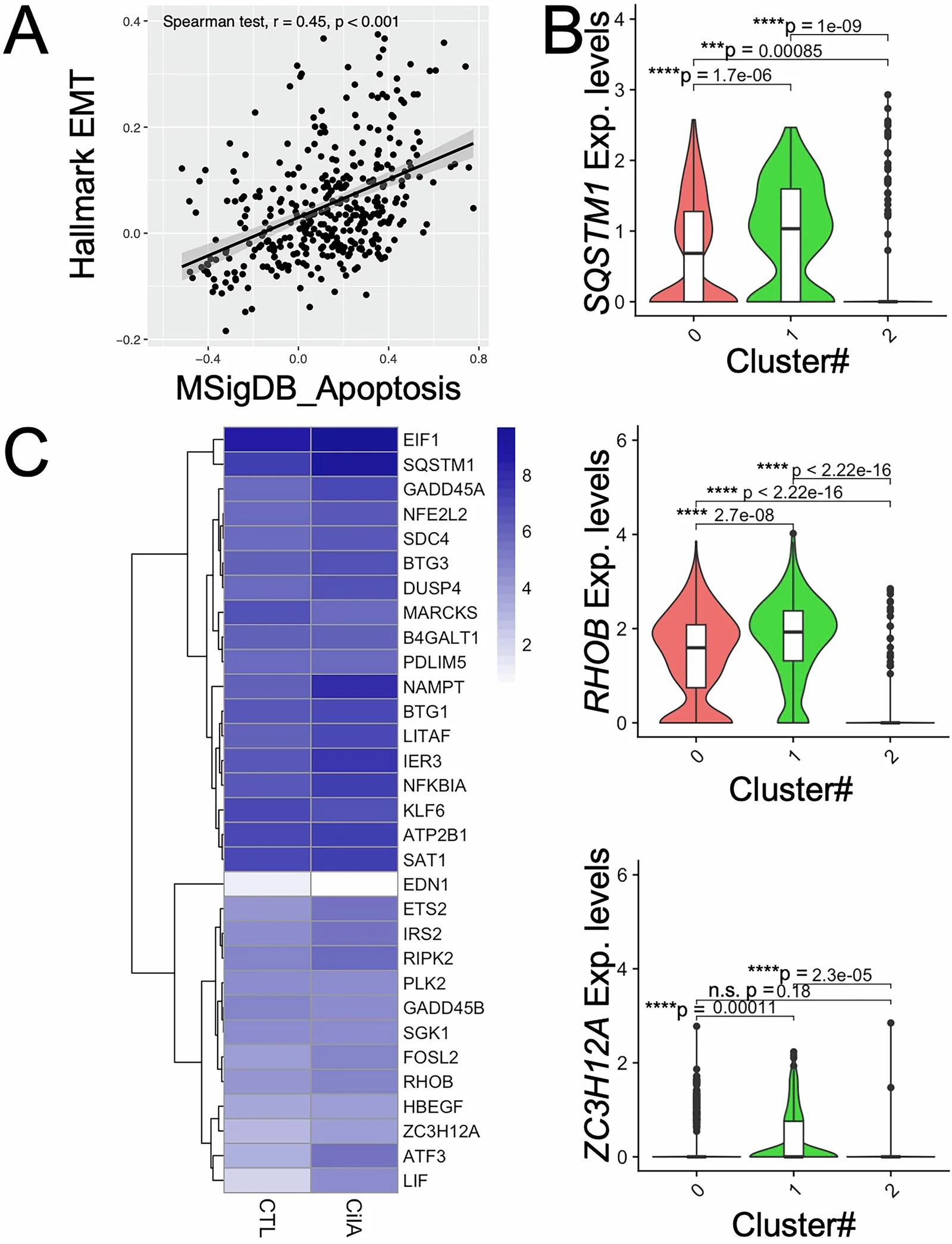
Mechanisms of EMT-cilium-dependent regulation of chemoresistance. (**A**) The correlation in the expression levels of EMT and apoptosis gene expression signatures in cancer cells from PDOs treated with Nao-3 was determined using a Spearman test, *r* = 0.45, ***P < 0.001. (**B**) Violin plots illustrating the expression levels of the indicated gene transcripts in cell clusters from DMSO-treated and Nao-3-treated PDOs. Kruskal–Wallis test: ns. *P* = 0.18; ***P = 0.00085; ****P < 0.0001. Box plots show the median (center line), the 25th and 75th percentiles (lower and upper bounds of the box), and whiskers extending up to 1.5 times the interquartile range from the box limits. Data points beyond this range are considered outliers and are shown individually. Cluster 0 *n* = 499, cluster 1 *n* = 281, cluster 2 *n* = 122. (**C**) Heatmap illustrating the expression levels of the indicated gene transcripts in HMLER shEcad cells treated with a vehicle control (CTL, DMSO) or Ciliobrevin A (CilA). GSE160549 dataset.

Having established that primary cilia are essential for the chemoresistance of the quasi-mesenchymal cells, we wanted to explore underlying mechanism(s). First, we compared the PDOs after short-term 24 h treatment with our ciliogenesis inhibitor Nao-3, versus the vehicle control, which represent the acute phase of drug response. We observed that Nao-3 repressed ciliogenesis, and induces caspase activation in this setting, by immunostaining PDOs for primary cilia (Arl13b and γTub) and cleaved-caspase 3 (CC3) markers (Fig. 5A). We then conducted scRNAseq analysis of dissociated cancer cells from control- or Nao-3-treated samples. Unsupervised clustering of cells from both samples revealed three distinct cell states in the analyzed PDOs (Cluster 0–2, Fig. 5B–D; Dataset EV6). We found that vehicle control PDOs were composed predominantly of cells belonging to cluster 0 and 2, whereas the Nao-3 treated PDOs were enriched for cells of cluster 1 and 2 cells (Fig. 5E). To understand the hallmarks of cluster 1 cells that arose upon Nao-3 exposure, we conducted unsupervised gene set enrichment analysis using the gene list defining this cluster. The most significantly enriched gene sets included signatures of cell death and EMT (Table EV1), supporting the notion that cells forming cluster 1 are enriched for quasi-mesenchymal cells primed to die by Nao-3. We notably found that cluster 1 showed significantly higher levels of a gene set associated with apoptosis (*P* < 2.2e-16, Fig. 5F,G; Table EV2). Additionally, we identified a significant correlation in the level of expression of apoptosis and EMT signatures in cancer cells from Nao-3-treated PDOs (*P* < 0.001, Fig. EV5A), corroborating our previous findings that Nao-3 impairs the viability of quasi-mesenchymal ciliated stem-like cells. Importantly, we also found activation of a transcriptional program regulated by NFκB among the top gene sets enriched in cluster 1 (*P* < 2.2e-16, Fig. 5H,I; Tables EV1 and 2), including genes coding for cell death inducers (e.g., *SQSTM1*, *RHOB*, *ZC3H12A*, Fig. EV5B) (Lee et al, 2021; Liu et al, 2001; Lu et al, 2016; Prendergast, 2001; Puissant et al, 2010). To determine whether cilia ablation is associated with activation of this subset of NFκB-regulated genes in a distinct context, we used our previously published RNA-seq dataset to compare the transcriptome of monolayer culture of quasi-mesenchymal HMLER shEcad cells treated with vehicle control or CilA, which had repressed ciliogenesis (Data ref: Wilson et al, 2021). Gratifyingly, we found that an NFκB signature, including our subset of NFκB-regulated genes of interest, is the most significantly enriched gene set in the list of upregulated genes upon CilA treatment in this distinct analysis (Table EV3; Fig. EV5C). We also compared the expression of a subset of NFκB-regulated genes, including genes coding for cell death-inducing proteins, in Taxol-treated quasi-mesenchymal ciliated HMLER cells (sgCTL) versus non-ciliated variants (sgIFT20), using real-time qPCR. These target genes were all detected at higher levels in sgIFT0 relative to sgCTL cells (Fig. 5J). Collectively, these findings demonstrate that either pharmacological or genetic ablation of primary cilia in quasi-mesenchymal cells induces expression of NFκB-regulated genes coding for cell death inducers.

We then used our HMLER genetic variants to determine whether NFκB signaling serves to induce the death of Taxol-treated quasi-mesenchymal cells in response to primary cilia ablation. Specifically, we compared the response of sgCTL ciliated HMLER and non-ciliated sgIFT20 variants to Taxol alone or a combination of Taxol and the NFκB signaling inhibitor Bay11-7085. We found that Bay11-7085 had no significant effect on the level of cell death in sgCTL cells, but it caused a significant reduction in the level of cell death triggered by genetic ablation of primary cilia in the sgIFT20 variants (*P* = 0.0299, Fig. 5K,L). Altogether, our data establish that primary cilia promote therapeutic resistance of quasi-mesenchymal cells, at least in part, by repressing NFκB-induced cell death.

## Discussion

The functional contribution of EMT-induced cell states to human cancer therapeutic resistance was poorly understood. Here, using patient-derived TNBC samples, we identify that a subset of quasi-mesenchymal cells that acquire a stem-like ciliated state mediate chemoresistance. We further establish that EMT-induced primary cilia promote chemoresistance in this context by repressing NFκB-regulated cell death. Our previously reported data established that an EMT/cilium axis promote TNBC tumorigenesis in a mouse carcinoma model (Guen et al, 2017; Wilson et al, 2021). The present data now reveal the critical role of EMT/ciliary signaling in human cancer resistance to therapy using patient-derived samples. Importantly, we developed a family of novel small-molecule drugs, termed naonedins, which repress primary ciliogenesis and suppress chemoresistance. Collectively, these findings establish a novel aspect of the cell biology of EMT-driven tumor resistance to therapy and a druggable vulnerability of a subset of hybrid E/M cells that drive human tumor therapeutic resistance.

We used PDOs to conduct our research. These have recently emerged as attractive patient-derived tumor models for studying the mechanisms of human tumor therapeutic response ex vivo (Bhatia et al, 2022; Dekkers et al, 2021; Sachs et al, 2018). Our data show solid evidence which demonstrate that PDOs represent appropriate tumor avatars for studying therapeutic resistance that is driven by phenotypic heterogeneity and plasticity of cancer cells. Our TNBC PDOs faithfully recapitulate EMT-associated inter- and intratumor phenotypic heterogeneity of cancer cells. Notably, they are comprised of cells residing in multiple quasi-mesenchymal states, enabling investigation on the role of these distinct intermediate EMT states in response to therapy. We demonstrate that a subset of late hybrid E/M cells, bear primary cilia, display a stem-like phenotype, and operate as therapy-resistant cells in the analyzed TNBC samples. These data illustrate the value of PDOs for functional studies on the causes and consequences of EMT activation in tumors using patient samples, and for the development of new pharmacological strategies targeting these plasticity programs to counteract therapeutic resistance in human cancers.

We also establish that primary cilia act as key determinant of EMT-driven cancer therapeutic resistance. Published studies using cell lines in vitro have suggested a role for primary cilia in resistance to therapy in lung, pancreatic, rhabdoid and glioblastoma models (Chao et al, 2022; Jenks et al, 2018; Kim et al, 2022; Wei et al, 2022). The mechanisms responsible for ciliogenesis induction and the cilium-mediated signaling events driving therapeutic resistance remained unclear. Our data, using patient-derived TNBC samples, demonstrate a critical role of EMT programs in the induction of ciliogenesis and reveal that primary cilia repress NFκB-regulated cell death upon therapy, in the subset of neoplastic cells that bear these organelles, resulting in their enrichment post-treatment. Importantly, these surviving cells are able to reseed PDOs, attesting to their stemness and arguing that they are responsible for driving therapeutic failure in patients. These data reveal new insights into the mechanisms of ciliogenesis and cilium-mediated tumor therapeutic resistance. We further demonstrate that primary cilia represent a druggable vulnerability in human cancer samples.

There is an emerging interest in targeting ciliogenesis for disease treatment, but a lack of inhibitors that efficiently and selectively achieve this goal. We established a phenotypic drug screening assay and developed new pharmacological inhibitors of ciliogenesis that effectively repress primary ciliogenesis in patient-derived samples. Interestingly, the five compounds we identified all belong to the same drug family, which we named Naonedins. The precise mechanism(s) by which these repress ciliogenesis remain to be determined, but we note that they share similarities with carbazoles, which have been reported to bind tubulin (Peronne et al, 2020). Thus, we speculate that Naonedins might act, at least in part, by disrupting ciliary and non-ciliary microtubule dynamics. Small molecules that inhibit cilium-dependent Hedgehog signaling have been developed and used for the treatment of basal cell carcinoma and medulloblastoma (Sharpe et al, 2015). However, targeting Hedgehog signaling alone, using such drugs, does not appear to be sufficient to cure these cancers. Malignant cells have been shown to acquire resistance to these inhibitors through both genetic and non-genetic mechanisms, enabling them to bypass cilium-dependent Hedgehog signaling and causing therapeutic failure (Biehs et al, 2018; Kuonen et al, 2019; Zhao et al, 2017). Naonedins could be an alternative of interest for the treatment of these tumors. Our data also suggest that interfering with NFκB signaling downstream of primary cilia could be an alternative to deplete the subset of ciliated cancer cells driving therapeutic failure in cancers. Importantly, we believe that targeting cilia and/or ciliary signaling applied in combination with other therapies is important for cancers, including for human TNBC.

Human TNBCs represent the most aggressive subtype of breast carcinomas that are associated with an unmet medical need (Grinda et al, 2021). The recent development of new therapeutics including antibody-drug conjugates enabled important progress for the treatment of these malignancies (Bardia et al, 2021). However, most advanced tumors remain associated with therapeutic resistance and cancer-related death (Bardia et al, 2021). Our work emphasizes the importance of considering and targeting phenotypic plasticity programs and related signaling circuitries to improve cancer treatment.

## Methods

**Table:** Reagents and tools table

Reagent/resource	Reference or source	Identifier or catalog number
**Experimental models**		
Patient-derived tissues (human)	Centre Eugène Marquis and Institut de Cancérologie du Grand Ouest	2012-A00682-41
RPE1	Guen et al, 2016	
HMLER	Guen et al, 2017	
**Recombinant DNA**		
pSPAX2	Wilson et al, 2021	
pMD2G	Wilson et al, 2021	
lentiCRISPR_sgCTL	Wilson et al, 2021	
lentiCRISPR_sgKiF3a	Wilson et al, 2021	
lentiCRISPR_sgIFT20	Wilson et al, 2021	
**Antibodies**		
Mouse anti-Arl13b (1:200)	NeuroMab	Cat#73-287
Rabbit antiAcetylated-a-tubulin (1:1000)	Cell Signaling Technology	Cat#5335
Rabbit anti-E-Cadherin (1:200 IF; 1:1000 WB)	Cell Signaling Technology	Cat#3195
Mouse anti-a-Tubulin (1:500)	Sigma-Aldrich	Cat#T9026
Rabbit anti-Cleaved Caspase 3 (1:200)	Cell Signaling Technology	Cat#9661
Mouse anti-Vimentin (1:500)	Dako	Cat#M0725
Goat anti-Mouse IgG2a 488 (1:1000)	Thermo-Fisher	Cat#A21131
Goat anti-Mouse 647 (1:1000)	Thermo-Fisher	Cat#A21236
Goat anti-Mouse IgG1 (1:1000)	Thermo-Fisher	Cat#21240
Goat anti-Rabbit 546 (1:500)	Thermo-Fisher	Cat#A11035
Rabbit anti-KIF3a (1:1000)	Proteintech	Cat#13930-1-AP
Rabbit anti-IFT20 (1:100)	Proteintech	Cat#13615-1-AP
Mouse anti-fibronectin (1:11,000)	BD Biosciences	Cat#610077
Mouse anti-actin	Millipore	Cat#MAB1501
HRP-Goat anti-mouse (1:5000)	Jackson ImmunoResearch	Cat#115-035-006
HRP-Goat anti-rabbit (1:5000)	Jackson ImmunoResearch	Cat#111-035-006
Alexa Fluor 555 anti-vimentin (1:1000)	abcam	Cat# ab203428
Anti-rabbit HRP	Roche	Cat#760-4311
Anti-mouse HRP	Roche	Cat#760-4310
Discovery Rhodamine Kit	Roche	Cat#760-233
Discovery Cy5 kit	Roche	Cat#760-238
Discovery FAM kit	Roche	Cat#760-243
**Oligonucleotides and other sequence-based reagents**		
GAPDH forward 5′-TGCACCACCAACTGCTTAGC-3′	Sigma	
GAPDH forward 5′-GGCATGGACTGTGGTCATGAG-3′	Sigma	
NFKBIA forward 5′-GGCCTTCCTCAACTTCCAGA-3′	Sigma	
NFKBIA reverse 5′-ATCACAGCCAGCTCCCAGAA-3′	Sigma	
NFKB1 reverse 5′-TTGGGTCCAGCAGTTACAGT-3′	Sigma	
RIPK2 forward 5′-GGGCCAGTATCAAGCACGAT-3′	Sigma	
RIPK2 reverse 5′-CGTGCTCGGTGAGGTATATCA-3′	Sigma	
ZC3H12A forward 5′-TGGTTTCCAACGACACATACC-3′	Sigma	
ZC3H12A reverse 5′-GTGAGTGGCTTCTTACGCAG-3′	Sigma	
RHOB forward 5′-CAAGAAGCTGGTGGTGGTG-3′	Sigma	
RHOB reverse 5′-TCGGCCACATAGTTCTCGAA-3′	Sigma	
SQSTM1 forward 5′-GTTGCCTTTTCCAGTGACGA-3′	Sigma	
SQSTM1 reverse 5′-TCGCAGATCACATTGGGGT-3’	Sigma	
**Chemicals, enzymes, and other reagents**		
DMEM/F12	Thermo-Fisher	11320033
Glutamax	Thermo-Fisher	35050061
HEPES	Thermo-Fisher	15630056
Human Noggi recombinant protein	Thermo-Fisher	120-10C-100UG
B-27	Thermo-Fisher	17504044
Nicotinamide	Sigma	n0636-100g
N-acetyl-L-cystein	Sigma	A9165
Primocin	Invivogen	ant-pm-05
Y27-632	AbMole	M1817
Heregulin	Peprotech	AF-100-03
FGF-7	Peprotech	AF-100-19
FGF-10	Peprotech	AF-100-26
A83-01	Sigma	SML0788-5MG
EGF	Peprotech	AF-100-15
SB202190	Sigma	S7067-5MG
BME	Bio-Techne	3533-010-02
Formaldehyde	Thermo-Fisher	11586711
Triton X-100	VWR	437002A
Normal Serum Block	Biolegend	927503
Tween-20	Thermo-Fisher	10113103
Hoechst	Invitrogen	H3570
RNA Plus Nucleospin	Macherey-Nagel	740990.250
Maxima first-strand cDNA synthesis kit	Thermo-Fisher	K1642
DMEM/F12 Glutamax	Thermo-Fisher	11559726
Fetal Bovine Serum (FBS)	Eurobio	CVFSVF00
Insulin	Sigma	91077 C
Hydrocortison	Sigma	H0888-5G
MEGM	Lonza	CC-3150
Naonedins	This study	
3D CellTiter-Glo	Promega	G7570
Paclitaxel	Sigma	T7191
Propidium Iodide	Miltenyi Biotech	130-093-233
TrypLe Express	Thermo-Fisher	12605036
Trypsin-EDTA 1X	Thermo-Fisher	25300054
EurobioGreen qPCR Mix	Eurobio	GAEMMX02L
Chromium Next GEM Chip G Single Cell Kit	10X Genomics	1000120
Dual Index Kit TT Set A	10X Genomics	1000215
Chromium™ Next GEM Single Cell 3’ Kit v3.1	10X Genomics	1000268
NovaSeq 6000 S2 Reagent Kit v1.5 (200 cycles)	Illumina	20028315
NovaSeq 6000 S1 Reagent Kit v1.5 (100 cycles)	Illumina	20028319
PhiX Control v3	Illumina	FC-110-3001
NEBNext Library Quant Kit for Illumina	NEB	E7630 L
**Software**		
ImageJ v1.54	https://imagej.nih.gov/ij/index.html	
Imaris		
Ghaphpad rism v8.4.2	https://www.graphpad.com/	
FlowJo v10.10	https://www.flowjo.com/	
**Other**		
Lightsheet Z1 microscope	Zeiss	
QTOWER3G	Analytik Jena	
IncuCyte Live Cell Analysis System	Essen Bioscience	
FACS Canto II cytometer	BD Biosciences	
Chromium Controller & Next GEM Accessory Kit	10X Genomics	
NovaSeq 6000 System	Illumina	

### Human samples

Patient-derived tissues were collected from breast cancer patients (females, 30–85 years old) that were diagnosed at the Centre Eugène Marquis and at the Institut de Cancérologie du Grand Ouest (THEREX Clinical Research protocol: biological collection. ID-RCB NUMBER: 2012-A00682-41). Informed consent was obtained from all subjects. The experiments conformed to the principles set out in the WMA Declaration of Helsinki and the Department of Health and Human Services Belmont Report. None received therapy prior to surgery except one patient (used to generate PDOs from patient sample 8). PDBs and PDOs derive from different tumors. Tissues were collected by a pathologist after resection by a surgeon. Some fragments were paraffin-embedded, other fragments were dissociated within 2 h after surgical resection (tumors). Briefly, breast tumor pieces were cut into small fragments (<2 mm^3^), which were then dissociated enzymatically and mechanically as described previously (Wilson et al, 2021). Viable cells were then cryopreserved before organoid culture or directly used for organoid growth.

### Patient-derived organoid culture and treatments

PDO culture was performed using a protocol adapted from Sachs and colleagues (Sachs et al, 2018). Briefly, cells were seeded in advanced DMEM/F12 (Thermo-Fisher) supplemented with glutamax, HEPES, 10% R-spondin 1, 10% noggin, 2% B-27, nicotinamide (10 mM), N-acetyl-l-cysteine (500 mM), primocin (100 µg/mL), Y-27632 (5 µM), heregulin β1 (5 nM), FGF-7 (5 ng/mL), FGF-10 (5 ng/mL), A83-01 (0.5 µM), EGF (5 ng/mL), SB202190 (1 µM) containing 5% matrigel. 200,000 cells were seeded per well in 24-well ultralow attachment plates (Corning). PDOs were collected at distinct passages (between 0 and 7) because of the difference in the organoid growth dynamics between distinct patient samples. The phenotypic appearance of PDOs over successive passages within each patient sample remained similar for each patient sample.

### Immunofluorescence and image analysis

PDOs and cells were fixed and stained according to our published protocols (Duclos et al, 2021; Dupuy et al, 2022; Wilson et al, 2021). The following primary antibodies were used: Arl13b (NeuroMab 73-287, 1:200), acetylated tubulin (Cell Signaling Technology 5335; 1:1000), E-cadherin (Cell Signaling 3195, 1:200), α-tubulin (Sigma-Aldrich T9026; 1:500), cleaved-caspase 3 (Cell Signaling 9661; 1:200) and vimentin (Dako M0725; 1:500). Secondary antibodies were anti-mouse IgG2A 488 (Thermo-Fisher A21131; 1:1000), anti-mouse 647 (Thermo-Fisher A21236, 1:1000), anti-mouse IgG1 647 (Thermo-Fisher A21240; 1:1000), anti-rabbit 546 (Thermo-Fisher A11035, 1:500), anti-rabbit HRP (Roche, 760-4311) and anti-mouse HRP (Roche, 760-4310). DISCOVERY Rhodamine, Cy5 and FAM kits (Roche, 760-233, 760-238, 760-243) were used. Mounted coverslips with cells were examined using 60X objective and a wide-field Zeiss microscope. Organoids were embedded in low-melting point agarose and analyzed using a Lightsheet Z1 Zeiss microscope. Z-stacks were deconvolved and analyzed with ImageJ and Imaris.

### Single-cell RNA sequencing

Single-cell RNA sequencing was conducted using the Chromium Single-Cell 3’ v3.1 kit from 10X Genomics, according to the manufacturer’s protocol. Libraries were sequenced on the NovaSeq 6000 platform (Paired-end, 28 bp Read1, 90pb Read2). Raw BCL files were demultiplexed and mapped to the reference genome (refdata-cellranger-GRCh38-3.0.0) using the Cell Ranger Software Suite (v.6.1.2). The raw data were extracted from 10X format files using the *Read10X* function from the Seurat package (v4.4.0) in R v.4.1.1. We applied filtering to the feature-barcode gene expression matrix to preserve only cells of high quality based on several metrics, including the number of unique molecular identifiers (UMI), the number of genes detected and the percentage of read mapped to mitochondrial genes. Threshold were set three median absolute deviations (MAD) using the *isOutlier* function from the scater R package (v.1.22.0) to identify and flag cells with UMI counts and genes detected per cell bellow and above this specified threshold. In addition, cells surpassing a threshold of three MAD for the percentage of mitochondrial reads were considered as non-viable and removed from the analysis. Doublets were identified using the scDblFinder R package (v.1.6.0), where the scDlbFinder score was calculated for each cell and the scDblFinder threshold was applied for doublet identification. Detailed quality control metrics are provided in Table EV4.

### Single-cell RNA sequencing data analysis

After data preprocessing, normalization was performed using the *NormalizeData* function, with default parameters, which implements a logarithmic normalization to the gene expression data. Data were centered and scaled using *ScaleData* function. During scaling step, regression of S and G2M phase scores was performed to minimized the influence of cell cycle effects in downstream analysis. We used the top 2000 highly variable genes from the normalized expression matrix, to perform the principal component analysis (PCA, *runPCA* Seurat function), and applied Louvain graph-based clustering on the 30 first principal components. The resulting data were projected onto an Uniform Manifold Approximation and Projection (UMAP) using the *runUMAP* function. We applied the FindNeighbords and *FindClusters* functions to identify distinct clusters. For each defined cluster, a gene markers analysis was conducted using the *FindAllMarkers* function with the parameters set as follows: only.pos = TRUE, min.pct=T, logfc.threshold = 0.25. The top five markers genes per cluster were selected on the average log2fold change and a heatmap was generated using the *DoHeatmap* function. The score signatures was calculated using the *AddModuleScore* function. For statistical tests comparing signature values between clusters, we utilize the *stat_compare_means* function from ggpubr R package (v.0.6.0).

Copy number variation analysis was performed using the InferCNV R package (v.1.16.0 https://github.com/broadinstitute/infercnv). The analysis was executed using the *run* function with this following parameter settings: denoise=TRUE, HMM = TRUE, leiden_resolution=1, leiden_method = ”simple”, leiden_function = ”modularity”, analysis_mode = ”cell”. Reference cells were selected from the N-1105-epi sample, using a publicly available dataset (Pal et al, 2021) 10.15252/embj.2020107333.

Pseudotime analysis was conducted using the R package slingshot (v.2.0.0) to perform single-cell trajectory analysis, utilizing the principal curves algorithm. The analysis was conducted using the following input: cluster labels derived from the resolution of 0.9 and reduced dimensions obtained from PCA, as described previously. UMAP coordinates were used and no specific starting point was selected, default parameters were used for the analysis. ComplexHeatmap (v.2.15.4), ggplot2 (v.3.4.4), dittoSeq (v.1.4.4) R packages were used for graphical representation.

### Real-time qPCR

Total RNA was isolated using the RNA Plus Nucleospin (Macherey-Nagel), and cDNAs were generated with random primers and Maxima first-strand cDNA synthesis kit (Thermo Scientific). Real-time qPCR reactions were performed with a QTOWER3G (Analytik Jena) using EurobioGreen qPCR Mix (Eurobio) and the following gene-specific primers. GAPDH: forward 5′- TGCACCACCAACTGCTTAGC -3′, reverse 5′-GGCATGGACTGTGGTCATGAG-3′; NFKBIA: forward 5′- GGCCTTCCTCAACTTCCAGA-3′, reverse 5′-ATCACAGCCAGCTCCCAGAA -3′; NFKB1: forward 5′-GGACTACCTGGTGCCTCTAG-3′, reverse 5′- TTGGGTCCAGCAGTTACAGT-3′; RIPK2: forward 5′-GGGCCAGTATCAAGCACGAT -3′, reverse 5′-CGTGCTCGGTGAGGTATATCA-3′; ZC3H12A: forward 5′- TGGTTTCCAACGACACATACC-3′, reverse 5′-GTGAGTGGCTTCTTACGCAG -3′; RHOB: forward 5′-CAAGAAGCTGGTGGTGGTG-3′, reverse 5′- TCGGCCACATAGTTCTCGAA-3′; SQSTM1: forward 5′- GTTGCCTTTTCCAGTGACGA-3′, reverse 5′-TCGCAGATCACATTGGGGT-3′. Expression levels were normalized to GAPDH.

### 2D cell culture and treatments

HMLER cells were cultured in 1:1 mixture ofin Dulbecco’s modified Eagle medium (DMEM/F12) supplemented with glutamax, 10% FBS, 0.01 mg/mL insulin, 0.48 µg/mL hydrocortisone, and complete mammary epithelial cell growth medium (MEGM) supplemented with bovine pituitary hormone (Lonza). For all ciliogenesis assays, cells were grown until high confluence and serum-starved. To repress ciliogenesis, inhibitors were added to DMEM/F12 medium without serum for 24 h. All cell lines were authenticated and tested for Mycoplasma regularly through PCR-based methods.

### Drug screening

RPE1 cells were cultured in DMEM/F12 supplemented with glutamax, 10% FBS, and penicillin/streptomycin. Cells were grown until high confluence and serum-starved in DMEM/F12 for 24 h. Drugs diluted in DMEM/F12 (10 μM) were added to the cells for 24 h. Cells were fixed and stained as discussed above. Ciliogenesis was analyzed using a custom-made Fiji plugin.

### Viability analyses

Viability of cancer cells in PDOs was measured through a 3D CellTiter-Glo assay according to the manufacturer’s instructions. Viability of HMLER cells was assessed in a 96-well flat-bottom plate (zell-kontakt). The cells were plated and then treated 24 h later with 0.1 µM and 1 µM Taxol or 0.1% DMSO in the presence of propidium iodide (Miltenyi Biotec). The plate was incubated in an IncuCyte Live Cell Analysis System (Essen Bioscience). Phase-contrast and fluorescence images of cells were acquired every 2 h for 30-40 h using the IncuCyte Zoom automated imaging system. Live cell analysis, Incucyte software was used for data analysis.

### FACS analyses

PDOs were digested with TrypLe Express (Thermo-Fisher) 30 min at 37 °C. The dissociated cells were fixed in cold 80% methanol for 5 min and were permeabilized with 0.1% Triton X-100 for 15 min on ice. Cells were stained with an anti-vimentin-alexa Fluor 555 (Abcam; ab203428; 1:1000) antibody for 30 min on ice and washed. Staining of cells was analyzed using a FACS Canto II cytometer (BD Biosciences). The data were processed using FlowJo.

### Western blot experiments

These experiments were conducted using standard procedures as described previously (Wilson et al, 2021). Western blots were performed using primary antibodies against KIF3A (Proteintech 13930-1-AP; 1:1000), IFT20 (Proteintech 13615-1-AP; 1:100), E-cadherin (Cell Signaling 3195; 1:1000), fibronectin (BD Biosciences 610077; 1:11,000) and actin (Millipore MAB1501; 1:2000) and secondary antibodies horseradish peroxidase-coupled anti-mouse (Jackson ImmunoResearch 115-035-006; 1:5000) or anti-rabbit (Jackson ImmunoResearch 111-035-006; 1:5000).

### Data and statistical analyses

Data were generated from the analysis of randomly selected triple-negative breast cancer patient samples among the list of samples available at clinical cancer centers. Selection of field of views in bioimaging studies of patient samples and of cell lines were collected randomly based on nuclei staining before the analysis of other criteria. All samples analyzed were included in the presented data. Blinding of sample names was used to reduce unconscious bias whenever possible. R and Prism were used to analyze data, draw graphs, and perform statistical analyses. We employed the Wilcoxon test to compare two groups and the Kruskal–Wallis test to compare multiple groups in transcriptomic studies. We used the Spearman test for correlative in expression analysis. We employed unpaired two-sided Student’s *t* test to compare two groups for the in bioimaging studies. Statistical significance levels are indicated as follows: not significant ns *P* > 0.05; *P ≤0.05; **P ≤0.01; ***P ≤0.001; ****P ≤0.0001. Minimal *P* values for Student’s *t* test are defined as *P* < 0.0001 and for Kruskal–Wallis test as *P* < 2.2e-16. Representative results of three or more independent experiments are shown. Power analysis was conducted for all statistical studies.

## Supplementary information

Table EV1

Table EV2

Table EV3

Table EV4

Peer Review File

Dataset EV1

Dataset EV2

Dataset EV3

Dataset EV4

Dataset EV5

Dataset EV6

Movie EV1

Movie EV2

Movie EV3

Source data Fig. 1

Source data Fig. 2

Source data Fig. 3

Source data Fig. 4

Source data Fig. 5

Expanded View Figures

## Data Availability

Transcriptomics data have been deposited in the GEO under accession code GSE298840. Data are also available from the Dataset Gene Expression Omnibus (GEO) accession number GSE160549 (Wilson et al 2021). The source data of this paper are collected in the following database record: biostudies:S-SCDT-10_1038-S44321-025-00289-1.
