# Supplementary figures and images for: EMT-ciliary signaling in quasi-mesenchymal-stem-like cells drives therapeutic resistance and is a druggable vulnerability in triple-negative breast cancer
Source: EMBO Mol Med. 2025 Aug 26;17(10):2536–61. doi: 10.1038/s44321-025-00289-1 (PMC12514032; doi:10.1038/s44321-025-00289-1)

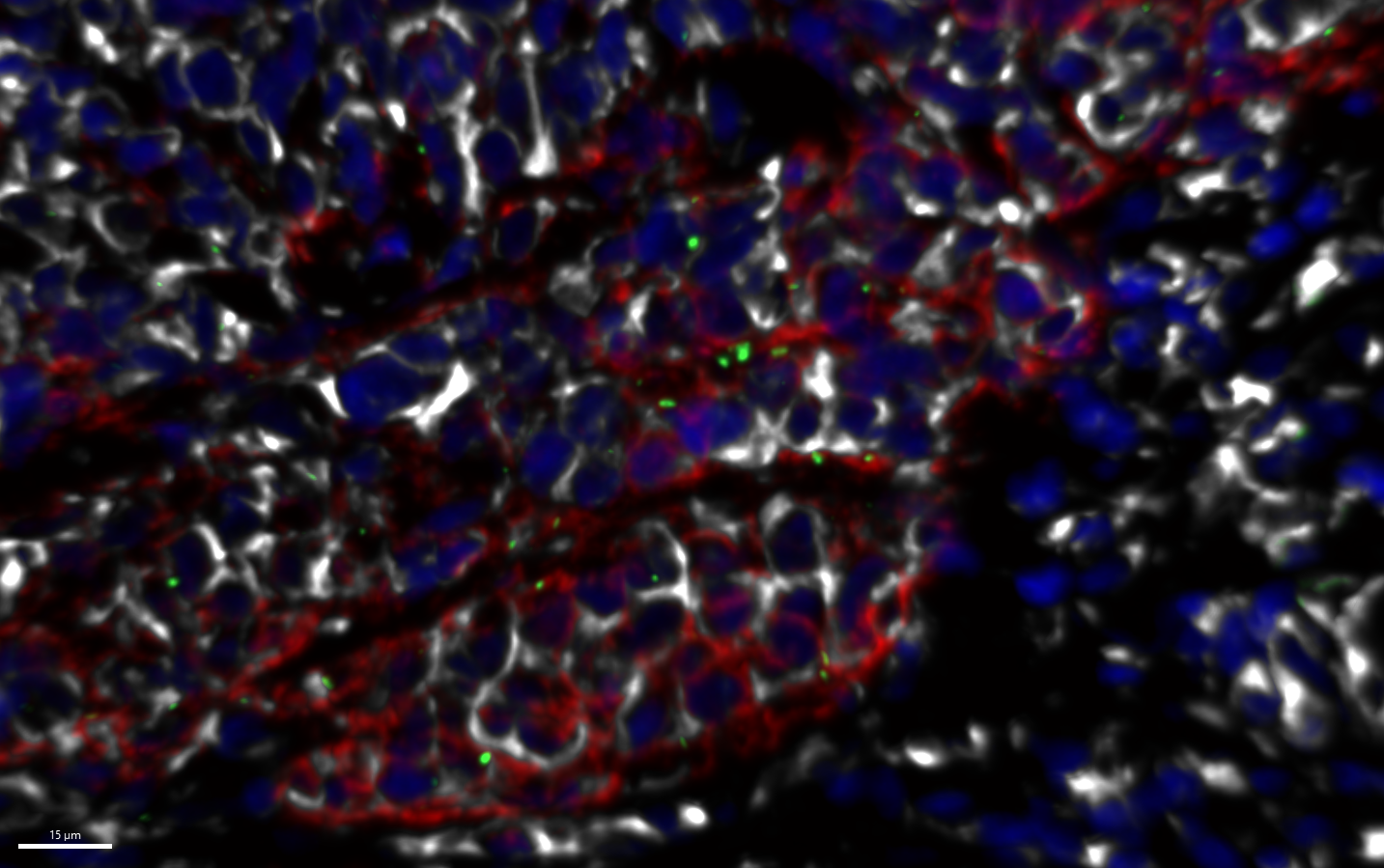

Supplement: Supplementary file 11 — Dataset EV6 [file 44321_2025_289_MOESM11_ESM.zip › EMM-2025-21514_SourceData_Figure 1/1A/PDBs_Pat#13_ROI1_merge.tif]

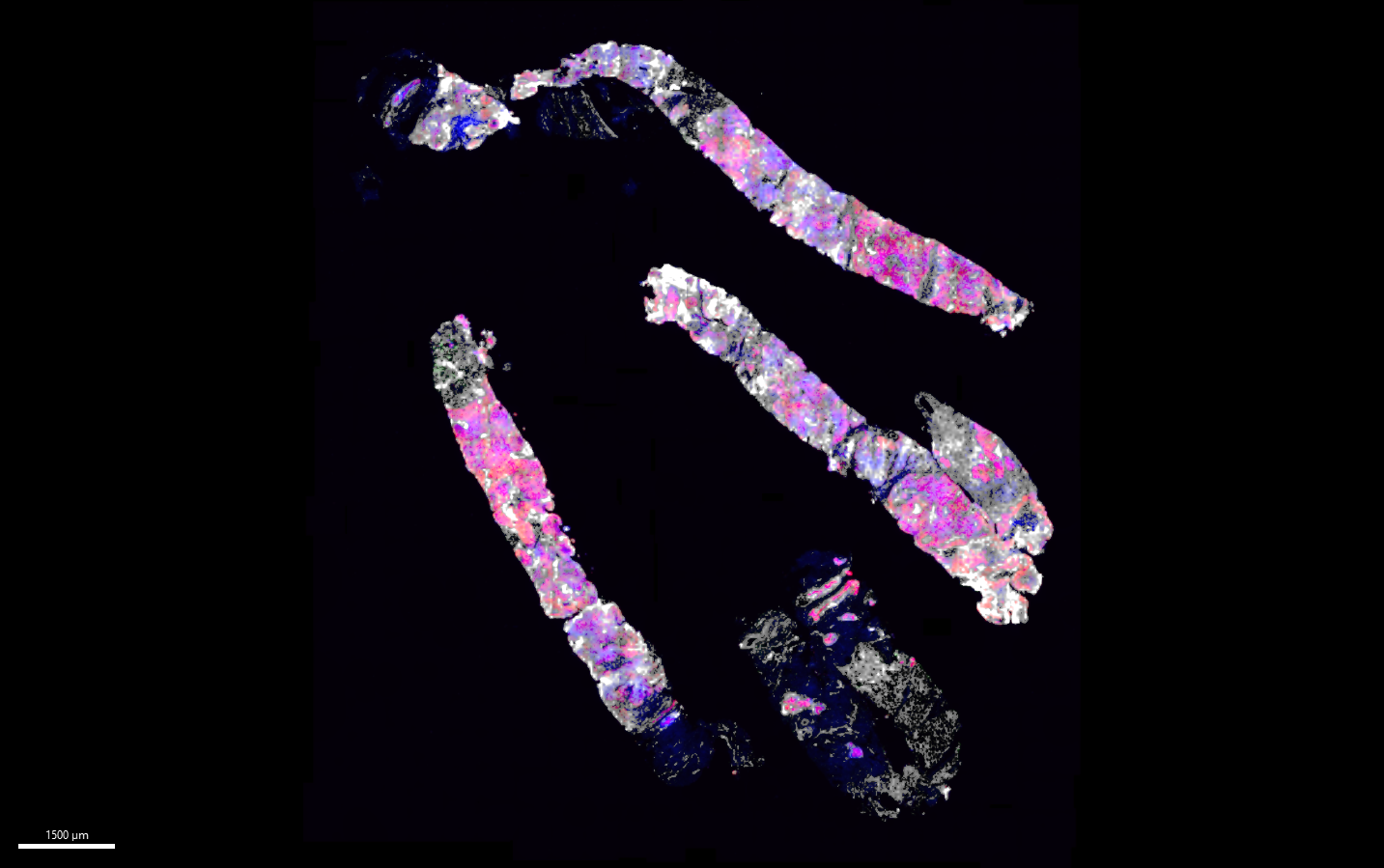

Supplement: Supplementary file 11 — Dataset EV6 [file 44321_2025_289_MOESM11_ESM.zip › EMM-2025-21514_SourceData_Figure 1/1A/PDBs_Pat#13.tif]

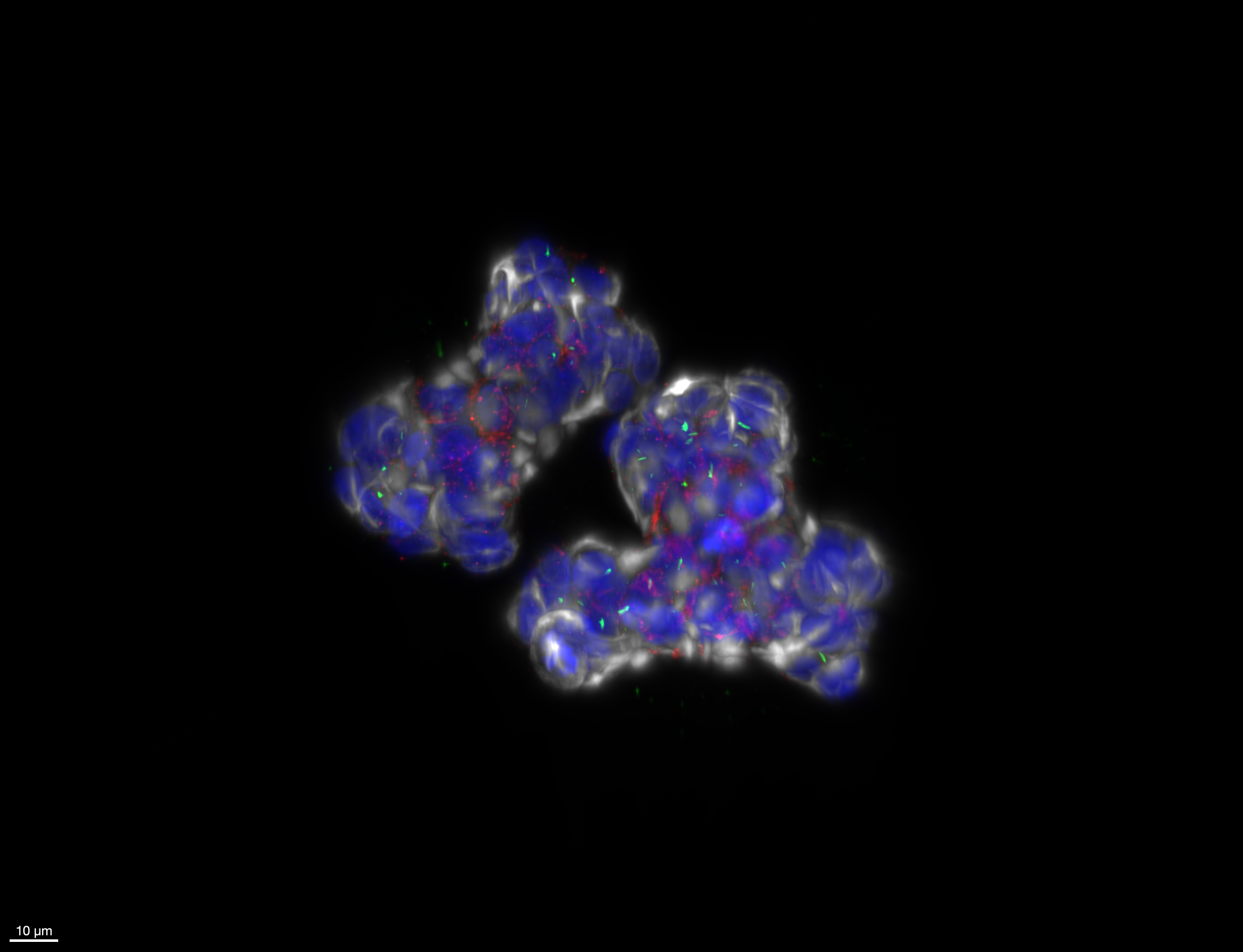

Supplement: Supplementary file 11 — Dataset EV6 [file 44321_2025_289_MOESM11_ESM.zip › EMM-2025-21514_SourceData_Figure 1/1I/Patient#3_PDOs_3D.tif]

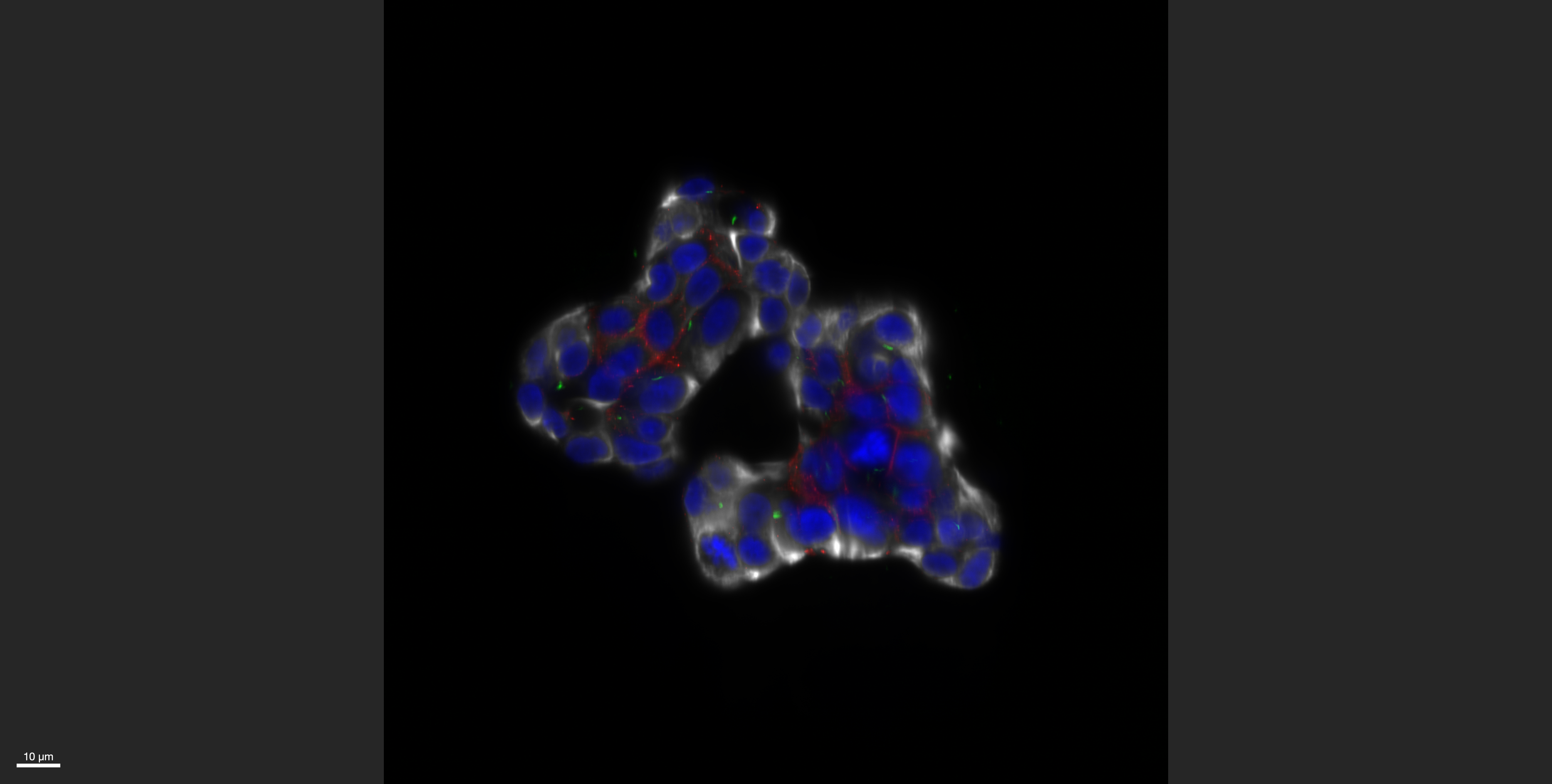

Supplement: Supplementary file 11 — Dataset EV6 [file 44321_2025_289_MOESM11_ESM.zip › EMM-2025-21514_SourceData_Figure 1/1I/Patient#3_PDOs_int.sec..tif]

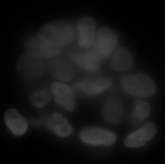

Supplement: Supplementary file 11 — Dataset EV6 [file 44321_2025_289_MOESM11_ESM.zip › EMM-2025-21514_SourceData_Figure 1/1E/Patient#5_PDOs_ROI1.tif]

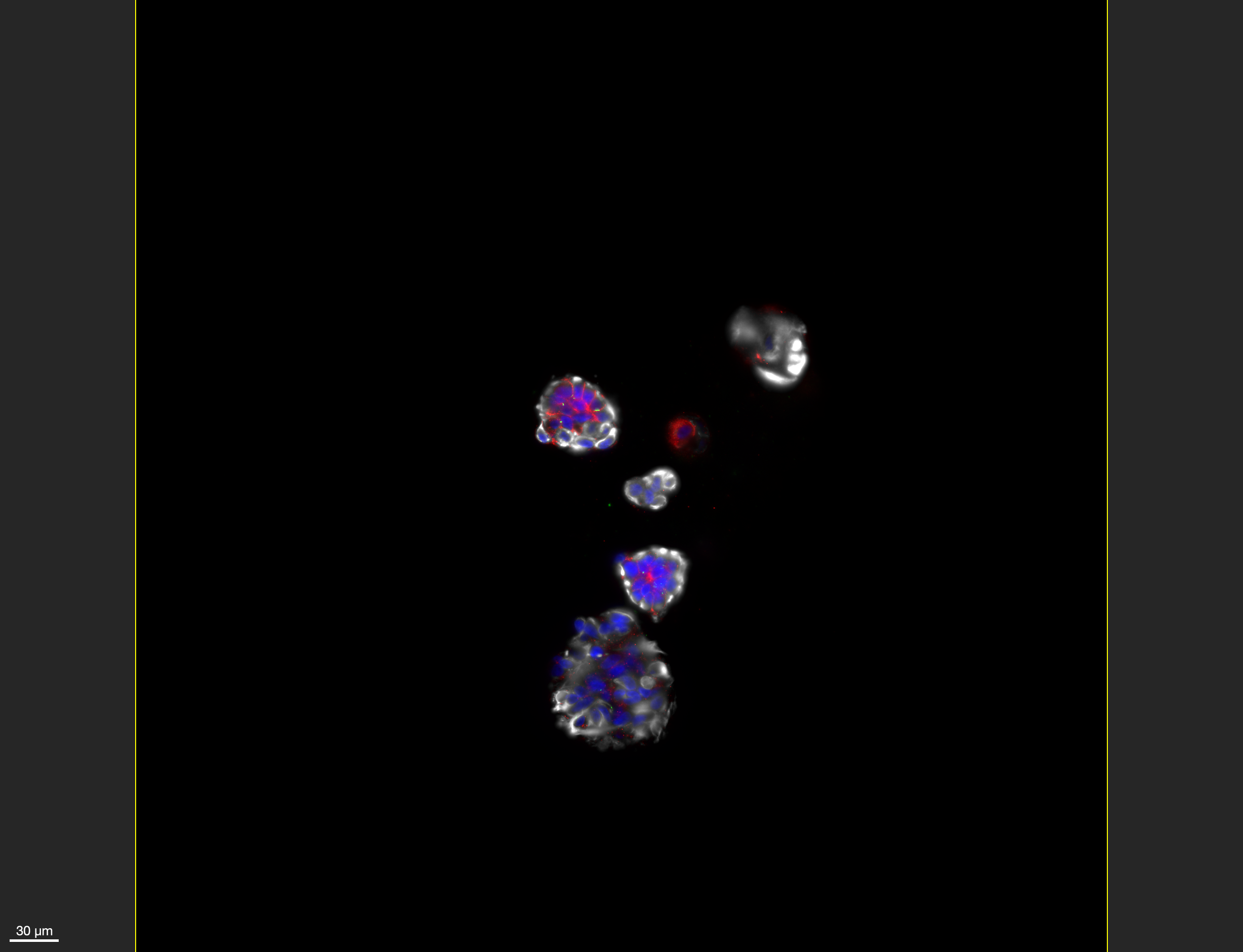

Supplement: Supplementary file 11 — Dataset EV6 [file 44321_2025_289_MOESM11_ESM.zip › EMM-2025-21514_SourceData_Figure 1/1E/Patient#5_PDOs.tif]

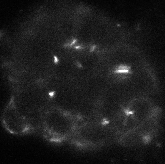

Supplement: Supplementary file 11 — Dataset EV6 [file 44321_2025_289_MOESM11_ESM.zip › EMM-2025-21514_SourceData_Figure 1/1E/Patient#5_PDOs_Arl13b.tif]

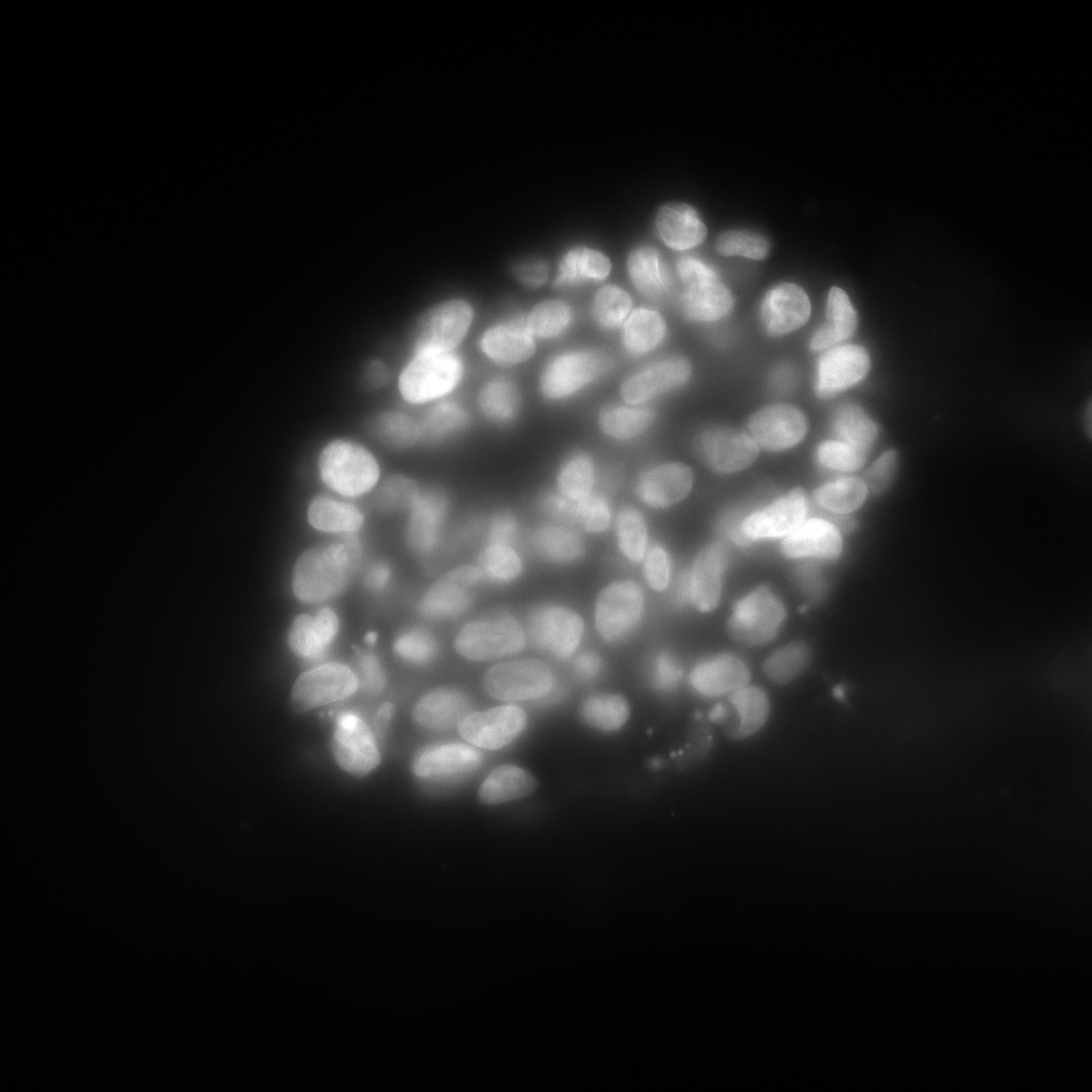

Supplement: Supplementary file 12 — Movie EV1 [file 44321_2025_289_MOESM12_ESM.zip › EMM-2025-21514_SourceData_Figure 2/2A/PDOs-Pat.6_Doxo.tif]

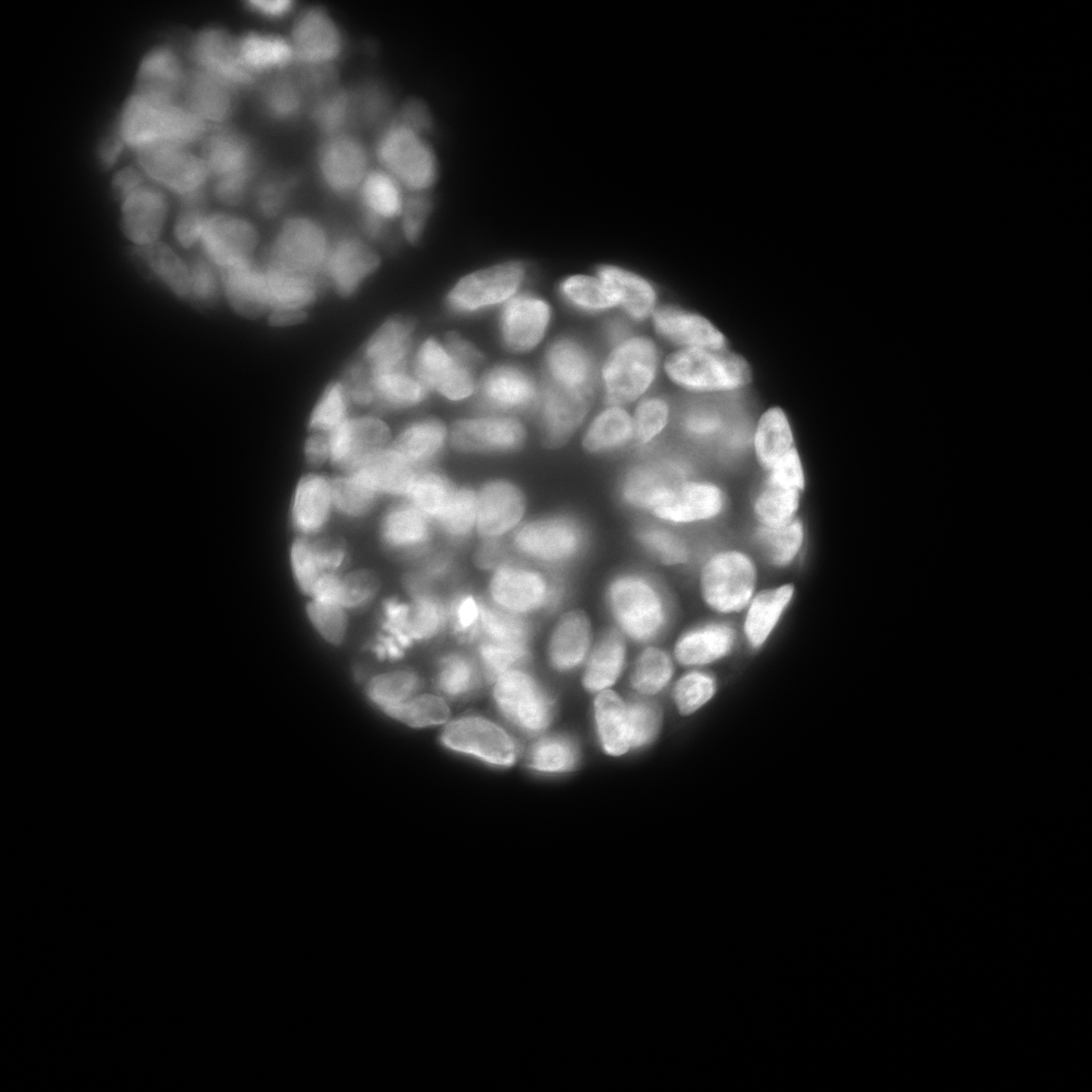

Supplement: Supplementary file 12 — Movie EV1 [file 44321_2025_289_MOESM12_ESM.zip › EMM-2025-21514_SourceData_Figure 2/2A/PDOs-Pat.7_Doxo.tif]

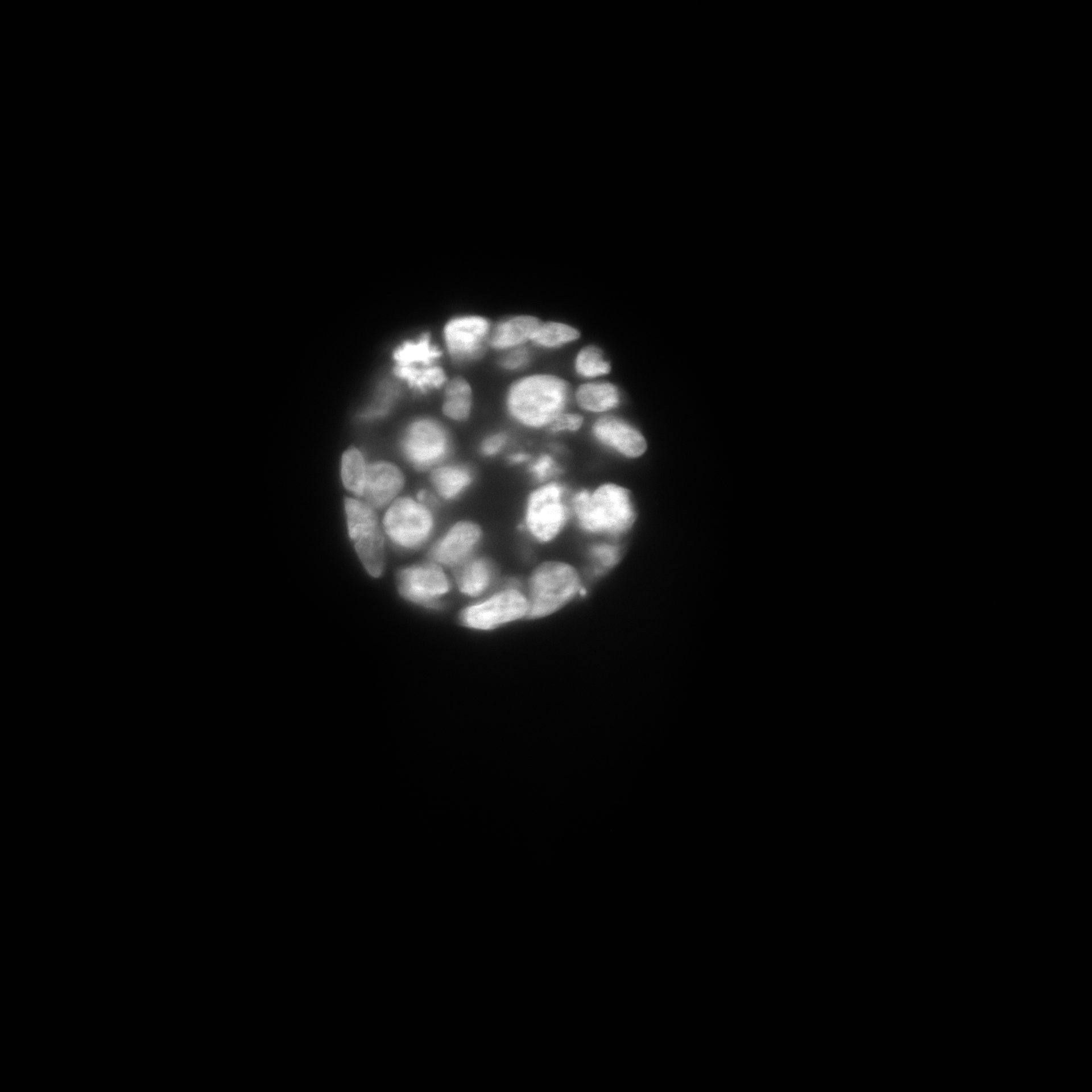

Supplement: Supplementary file 12 — Movie EV1 [file 44321_2025_289_MOESM12_ESM.zip › EMM-2025-21514_SourceData_Figure 2/2A/PDOs-Pat.7_Taxol.tif]

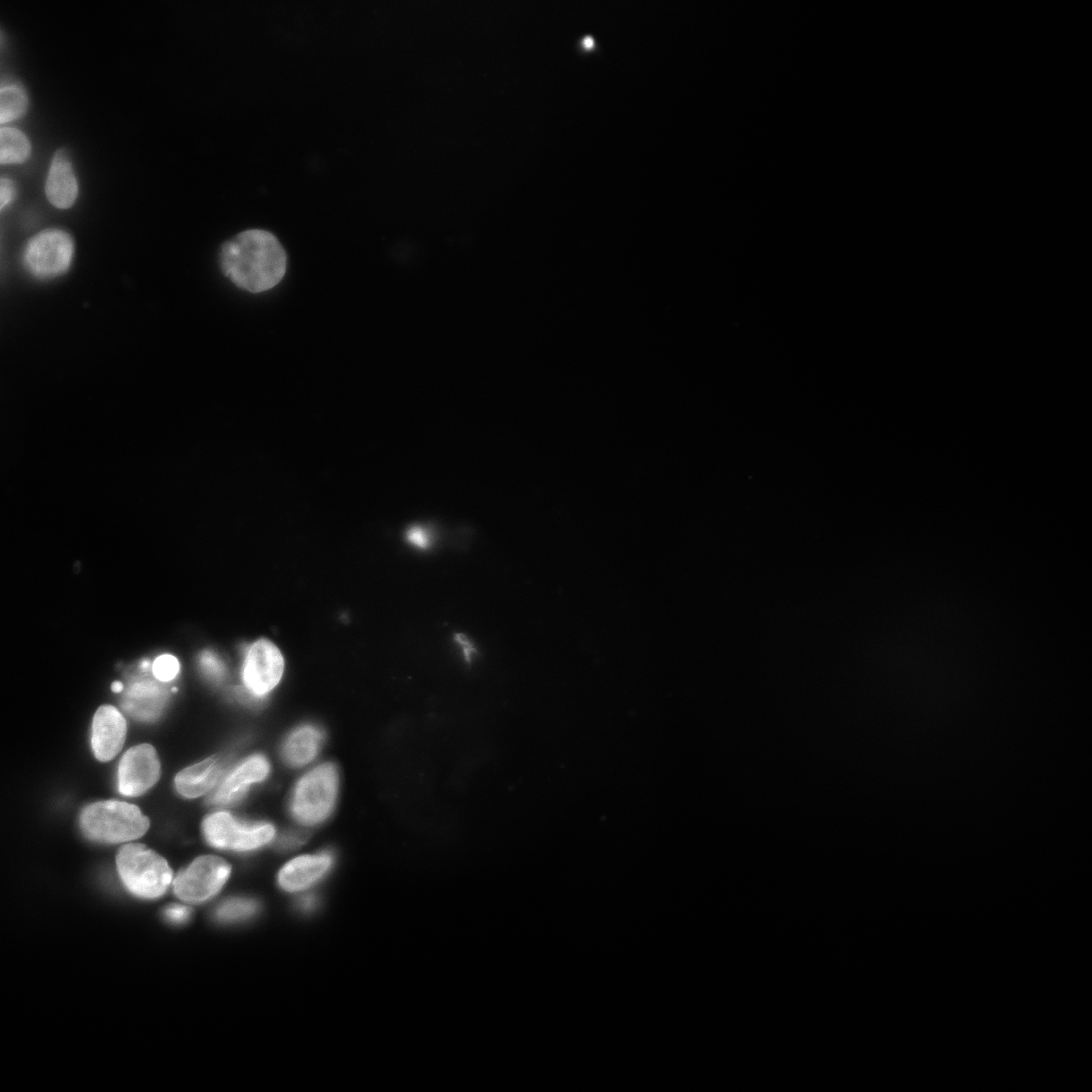

Supplement: Supplementary file 12 — Movie EV1 [file 44321_2025_289_MOESM12_ESM.zip › EMM-2025-21514_SourceData_Figure 2/2A/PDOs-Pat.3_CTL.tif]

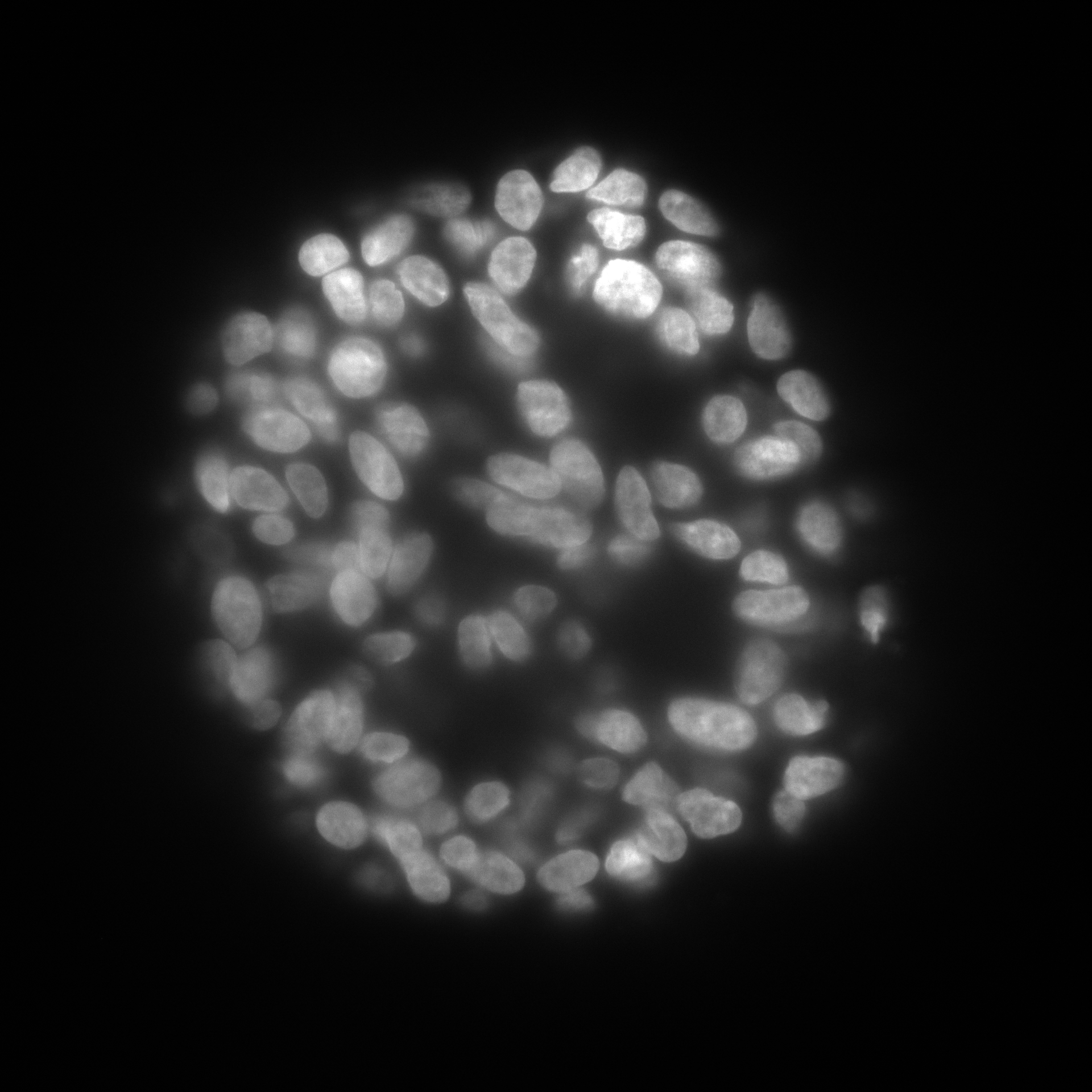

Supplement: Supplementary file 12 — Movie EV1 [file 44321_2025_289_MOESM12_ESM.zip › EMM-2025-21514_SourceData_Figure 2/2A/PDOs-Pat.6_Taxol.tif]

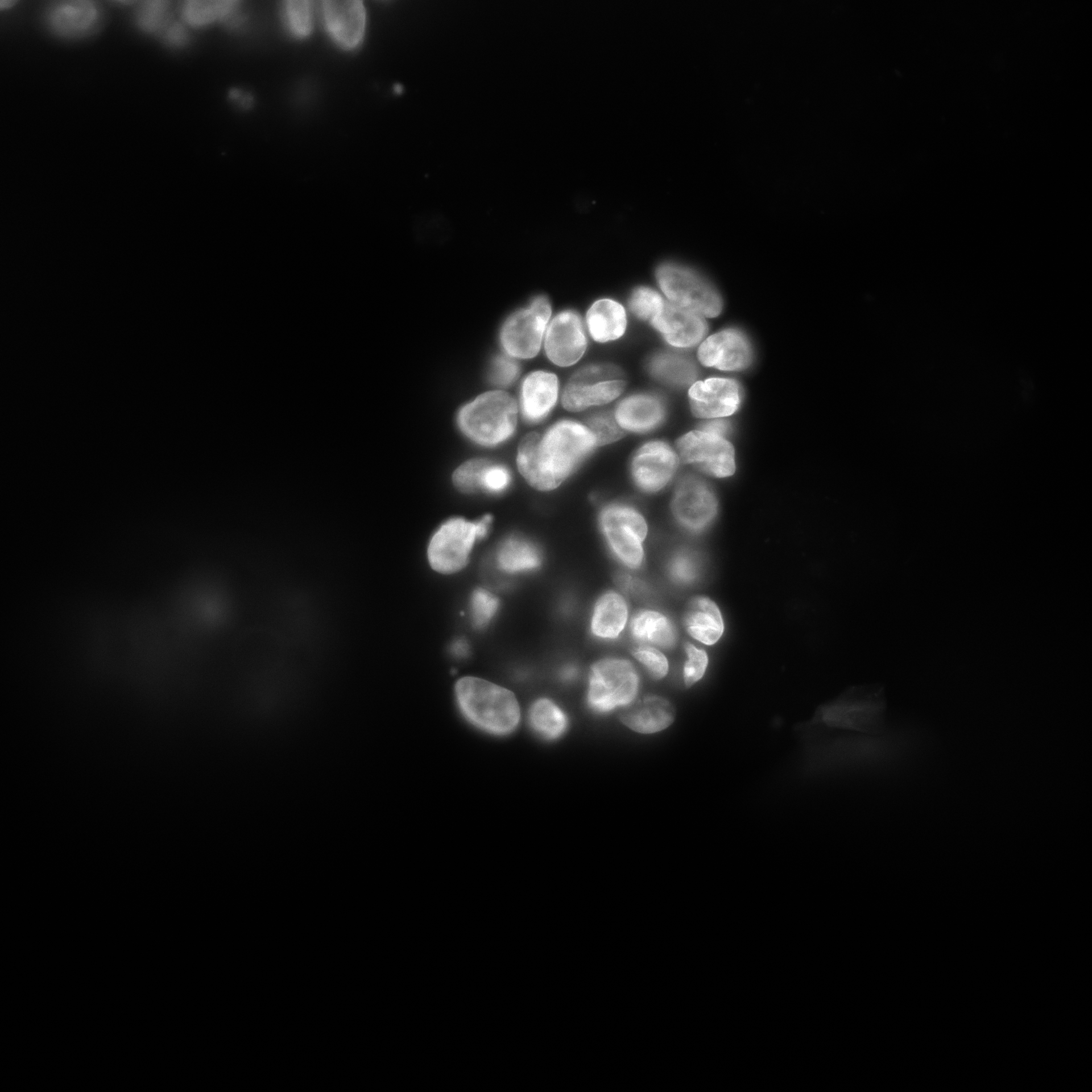

Supplement: Supplementary file 12 — Movie EV1 [file 44321_2025_289_MOESM12_ESM.zip › EMM-2025-21514_SourceData_Figure 2/2A/PDOs-Pat.3_Taxol.tif]

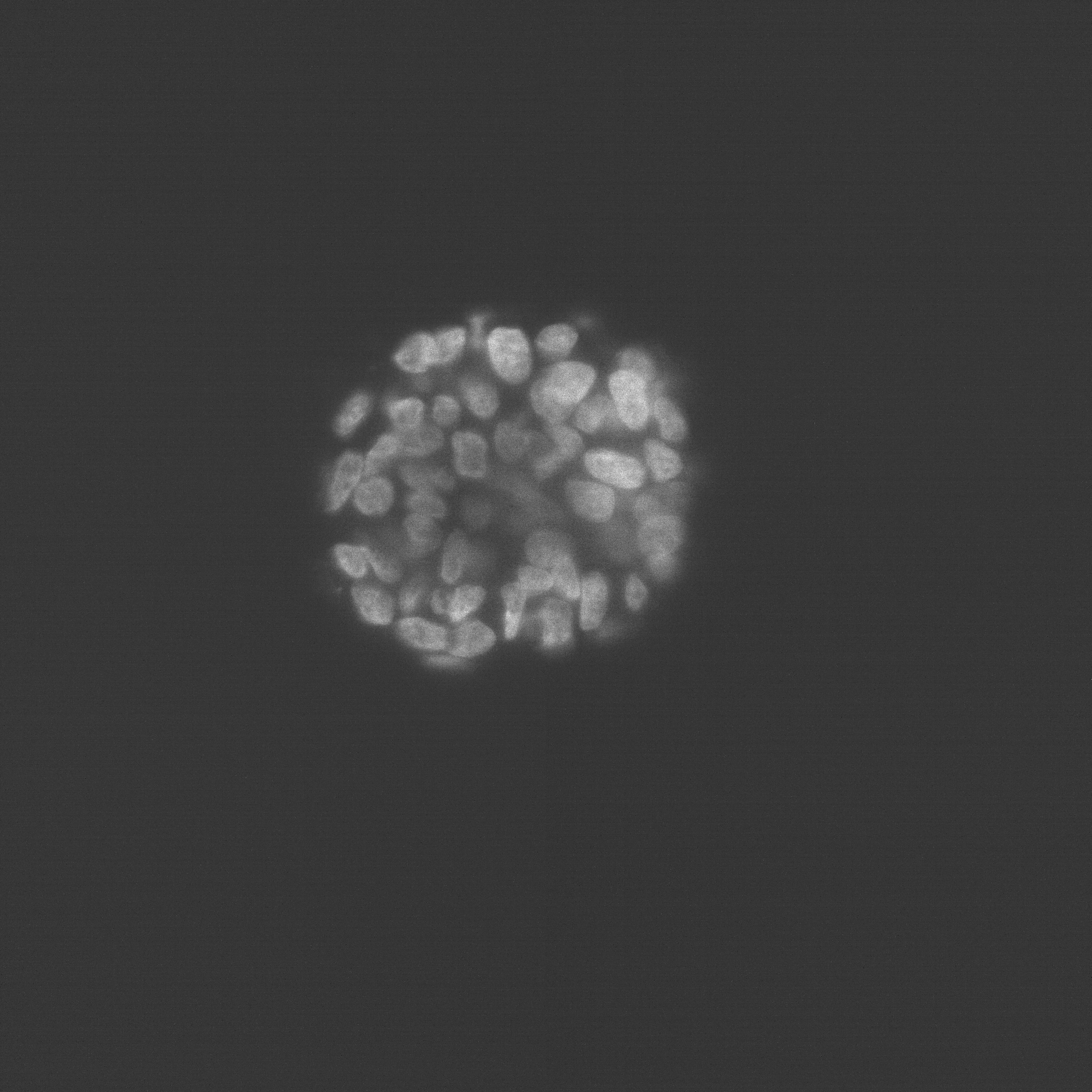

Supplement: Supplementary file 12 — Movie EV1 [file 44321_2025_289_MOESM12_ESM.zip › EMM-2025-21514_SourceData_Figure 2/2A/PDOs-Pat.7_CTL.tif]

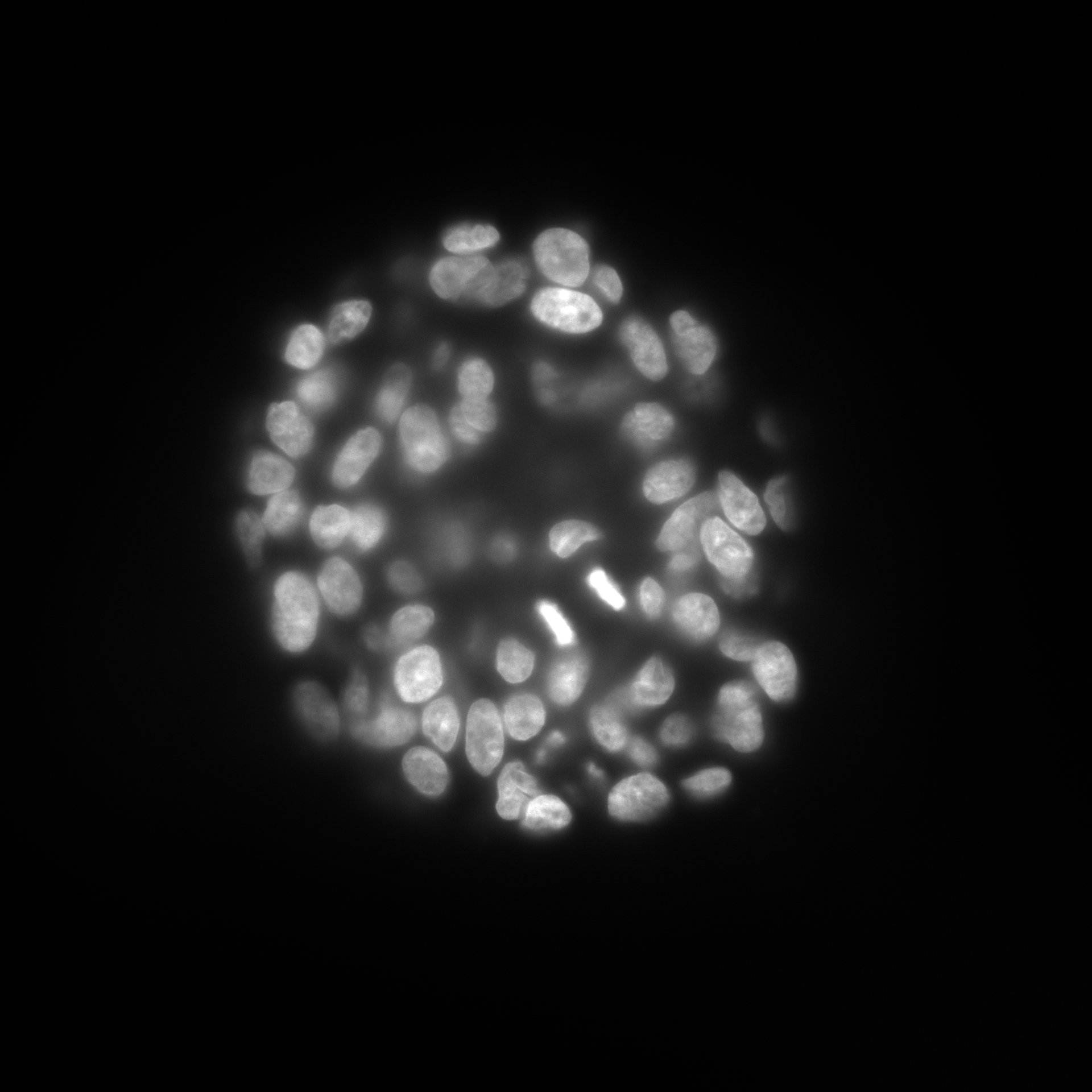

Supplement: Supplementary file 12 — Movie EV1 [file 44321_2025_289_MOESM12_ESM.zip › EMM-2025-21514_SourceData_Figure 2/2A/PDOs-Pat.6_CTL.tif]

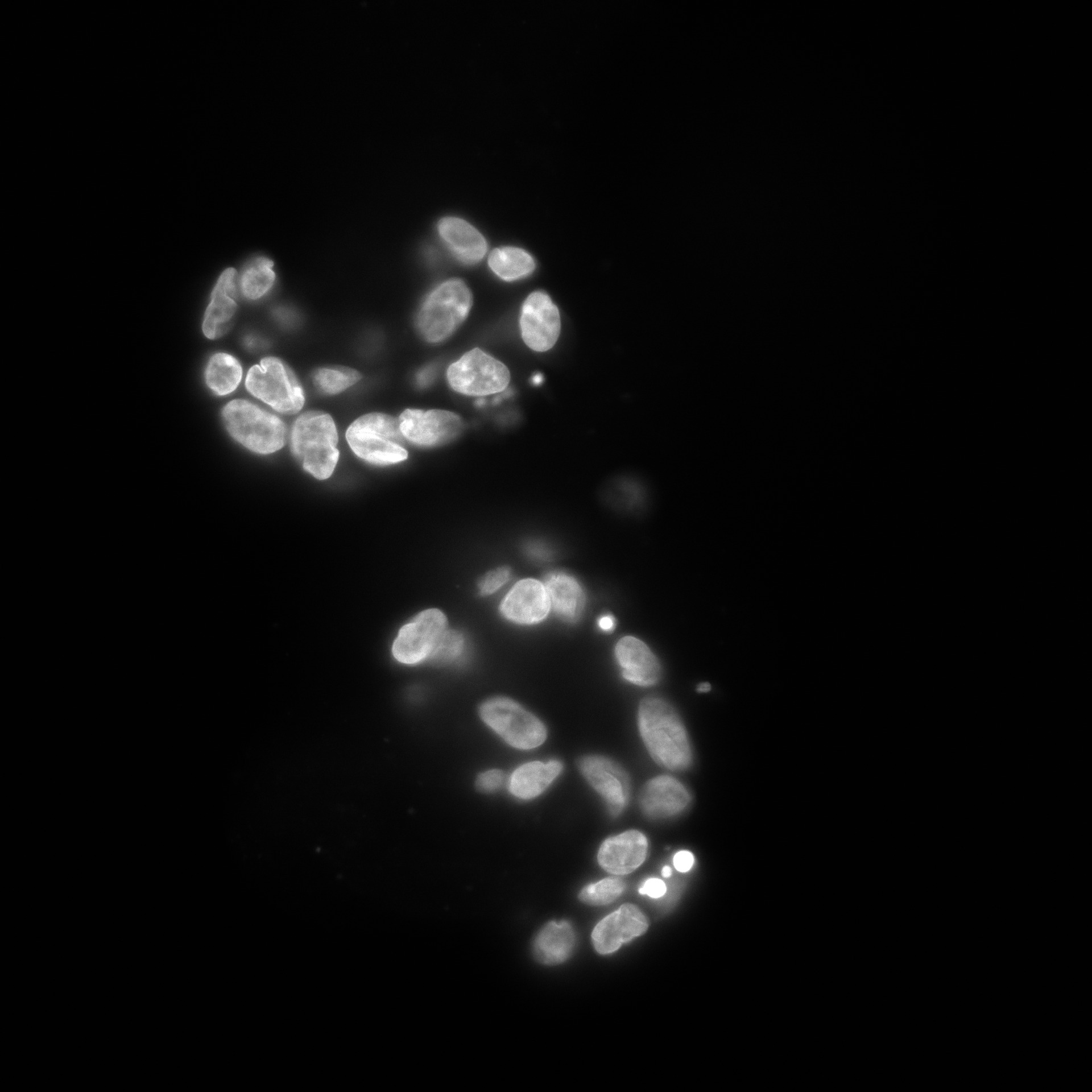

Supplement: Supplementary file 12 — Movie EV1 [file 44321_2025_289_MOESM12_ESM.zip › EMM-2025-21514_SourceData_Figure 2/2A/PDOs-Pat.3_Doxo.tif]

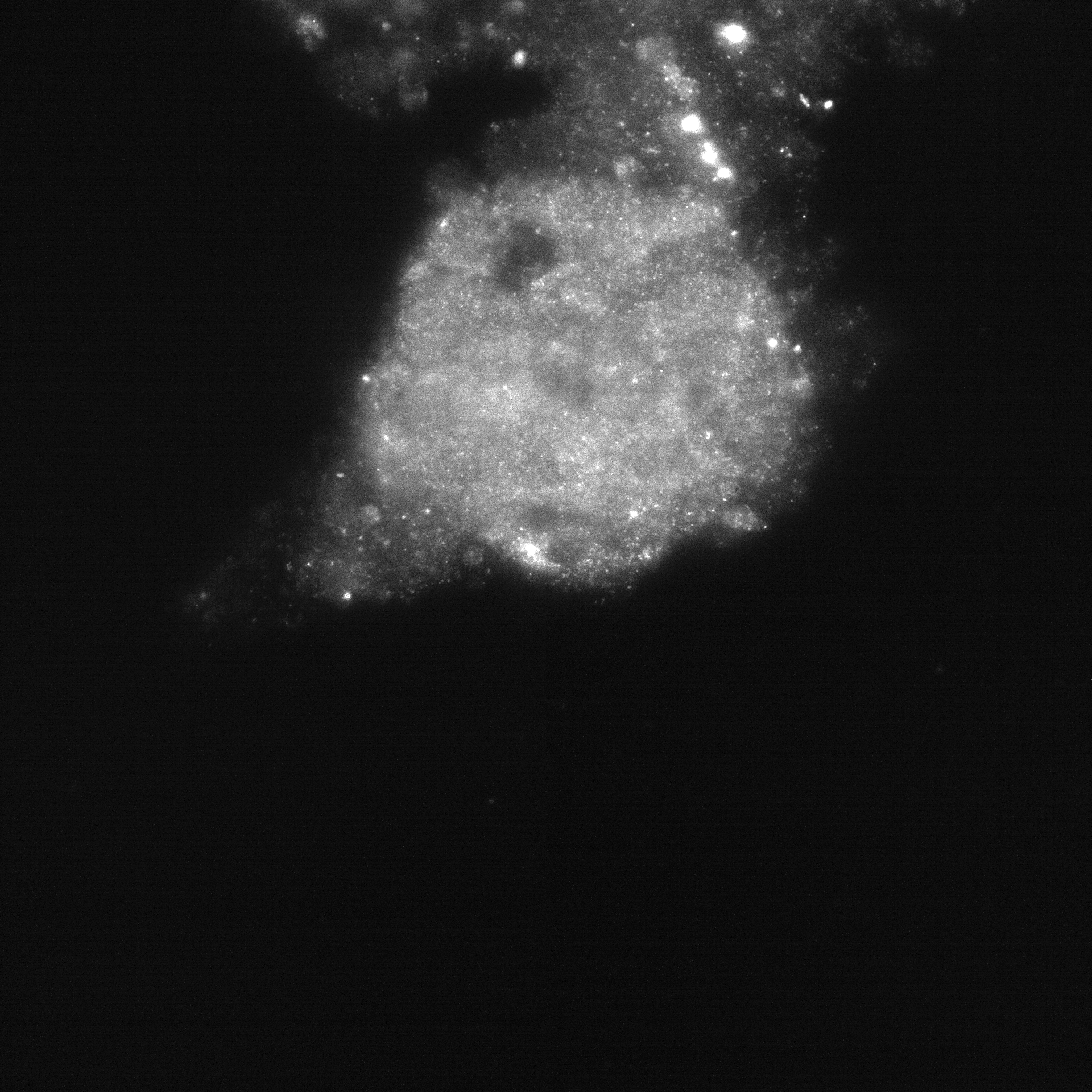

Supplement: Supplementary file 13 — Movie EV2 [file 44321_2025_289_MOESM13_ESM.zip › EMM-2025-21514_SourceData_Figure 3/3B/PDOs-Pat.3_Nao-3.tif]

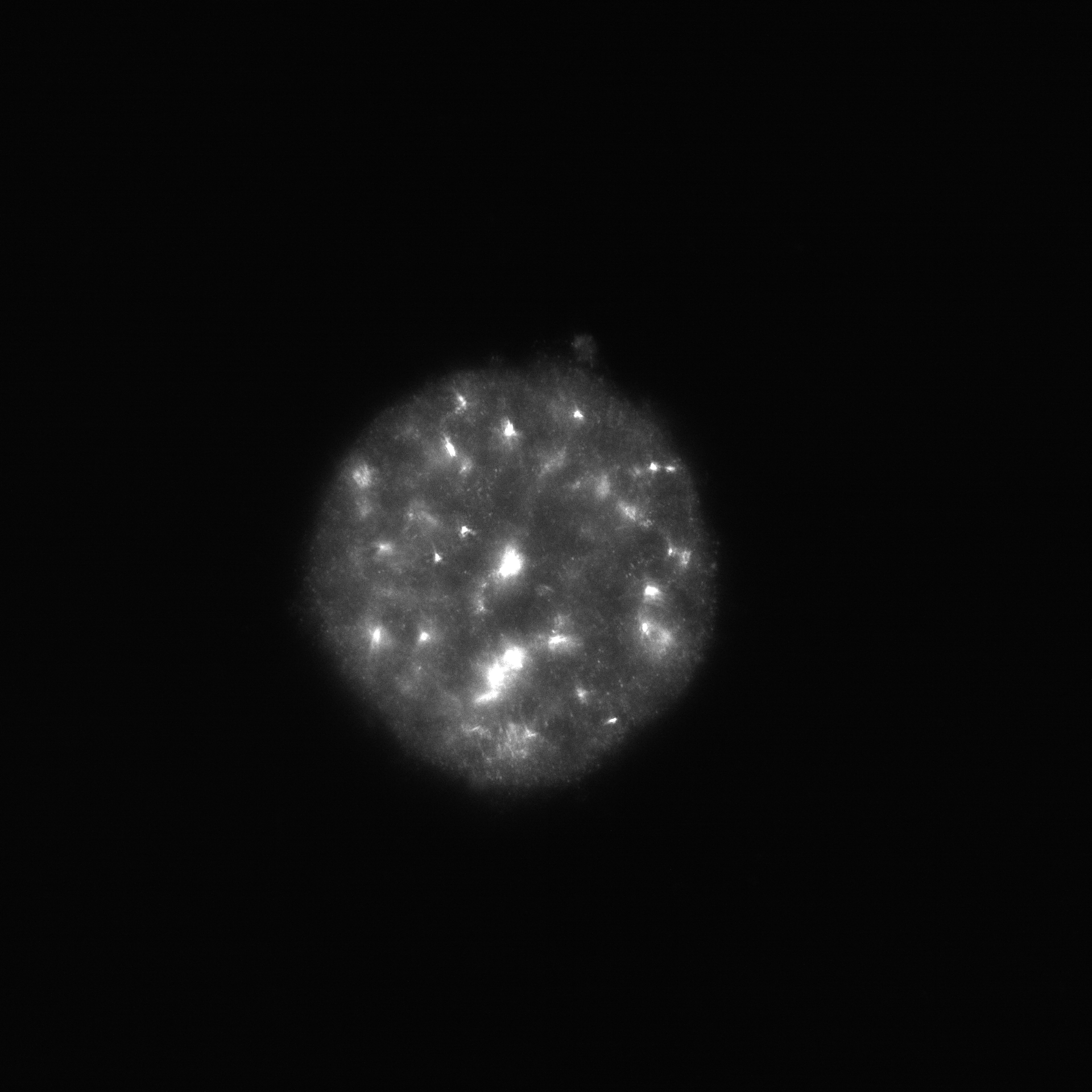

Supplement: Supplementary file 13 — Movie EV2 [file 44321_2025_289_MOESM13_ESM.zip › EMM-2025-21514_SourceData_Figure 3/3B/PDOs-Pat.3_CTL.tif]

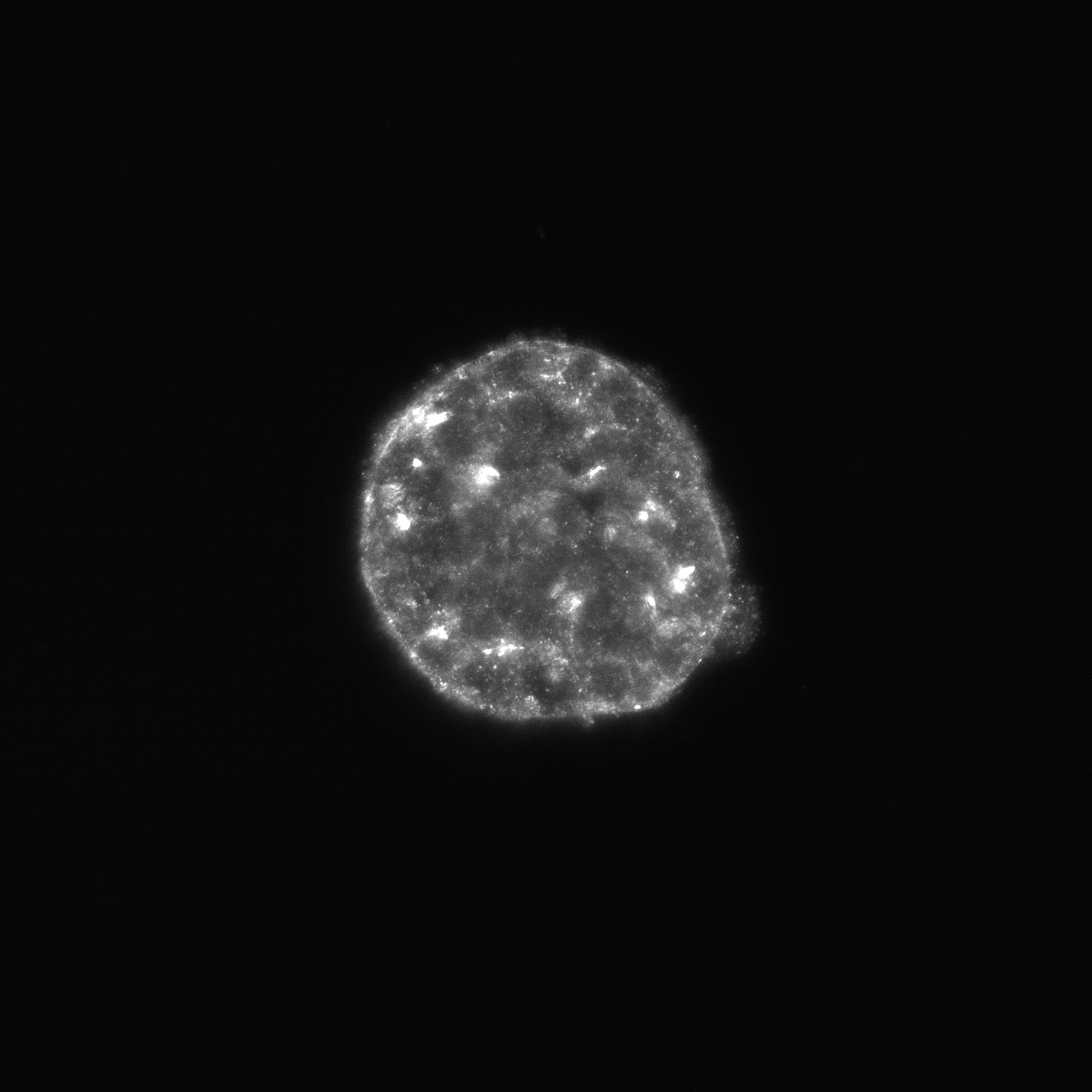

Supplement: Supplementary file 13 — Movie EV2 [file 44321_2025_289_MOESM13_ESM.zip › EMM-2025-21514_SourceData_Figure 3/3B/PDOs-Pat.7_CTL.tif]

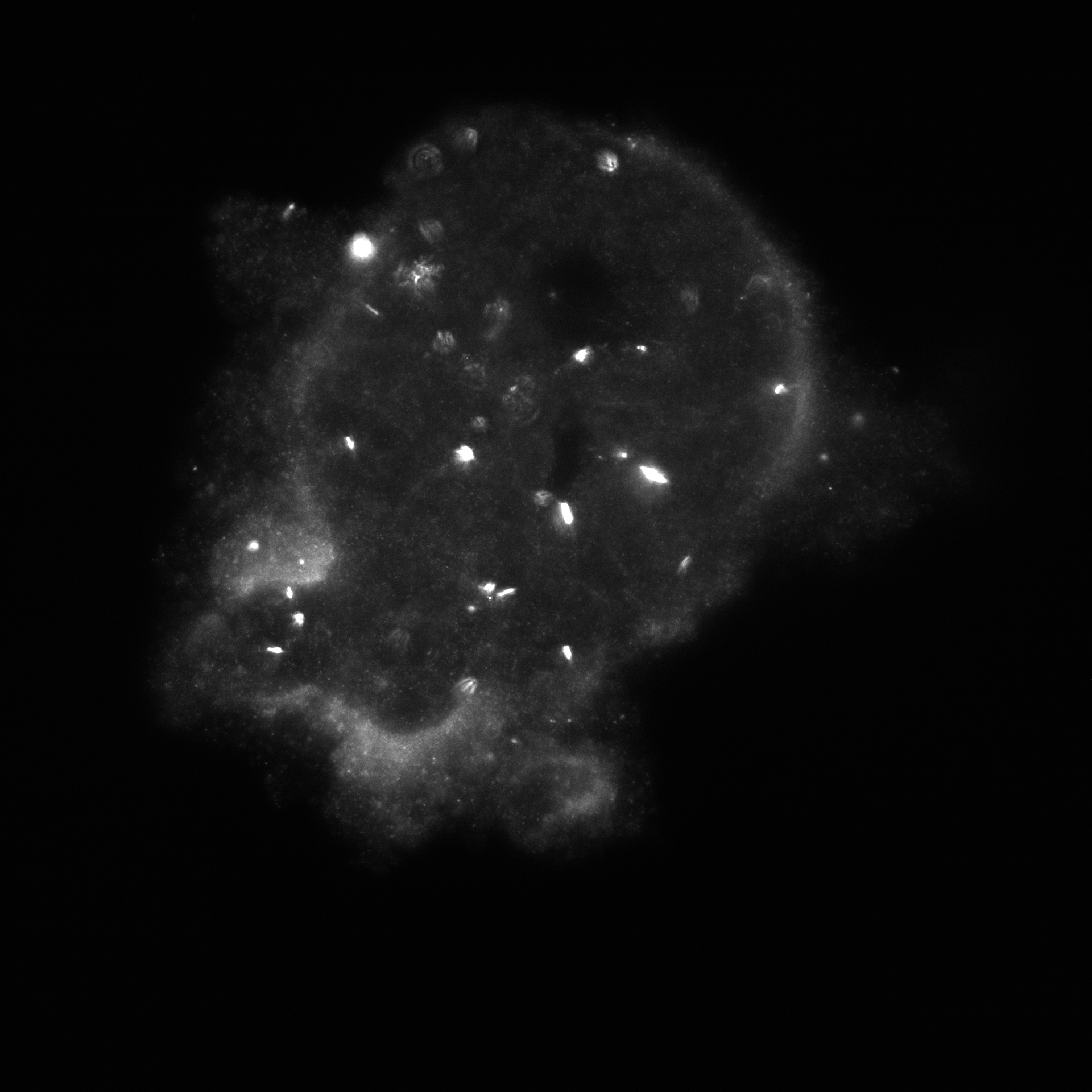

Supplement: Supplementary file 13 — Movie EV2 [file 44321_2025_289_MOESM13_ESM.zip › EMM-2025-21514_SourceData_Figure 3/3B/PDOs-Pat.6_CTL.tif]

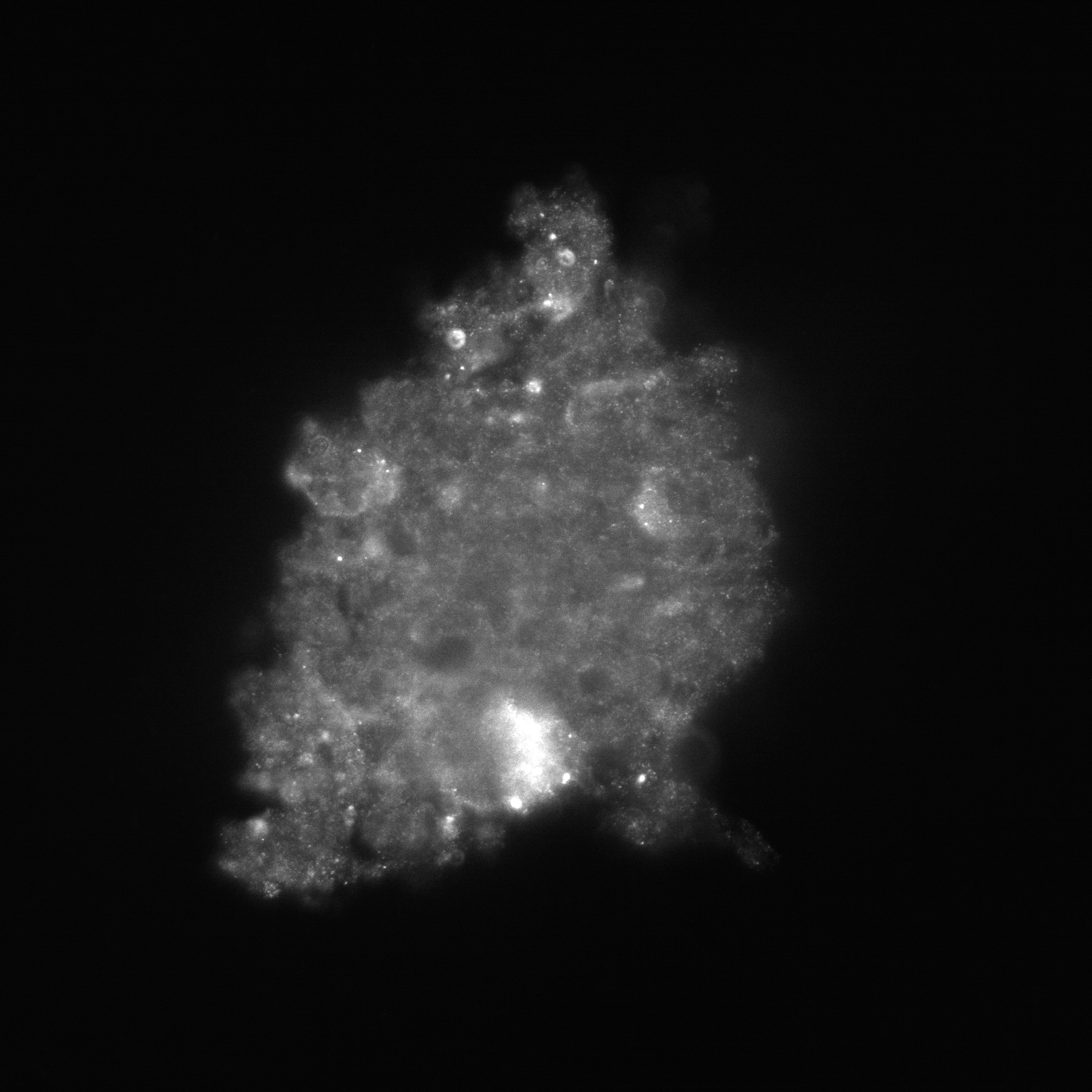

Supplement: Supplementary file 13 — Movie EV2 [file 44321_2025_289_MOESM13_ESM.zip › EMM-2025-21514_SourceData_Figure 3/3B/PDOs-Pat.7_Nao-3.tif]

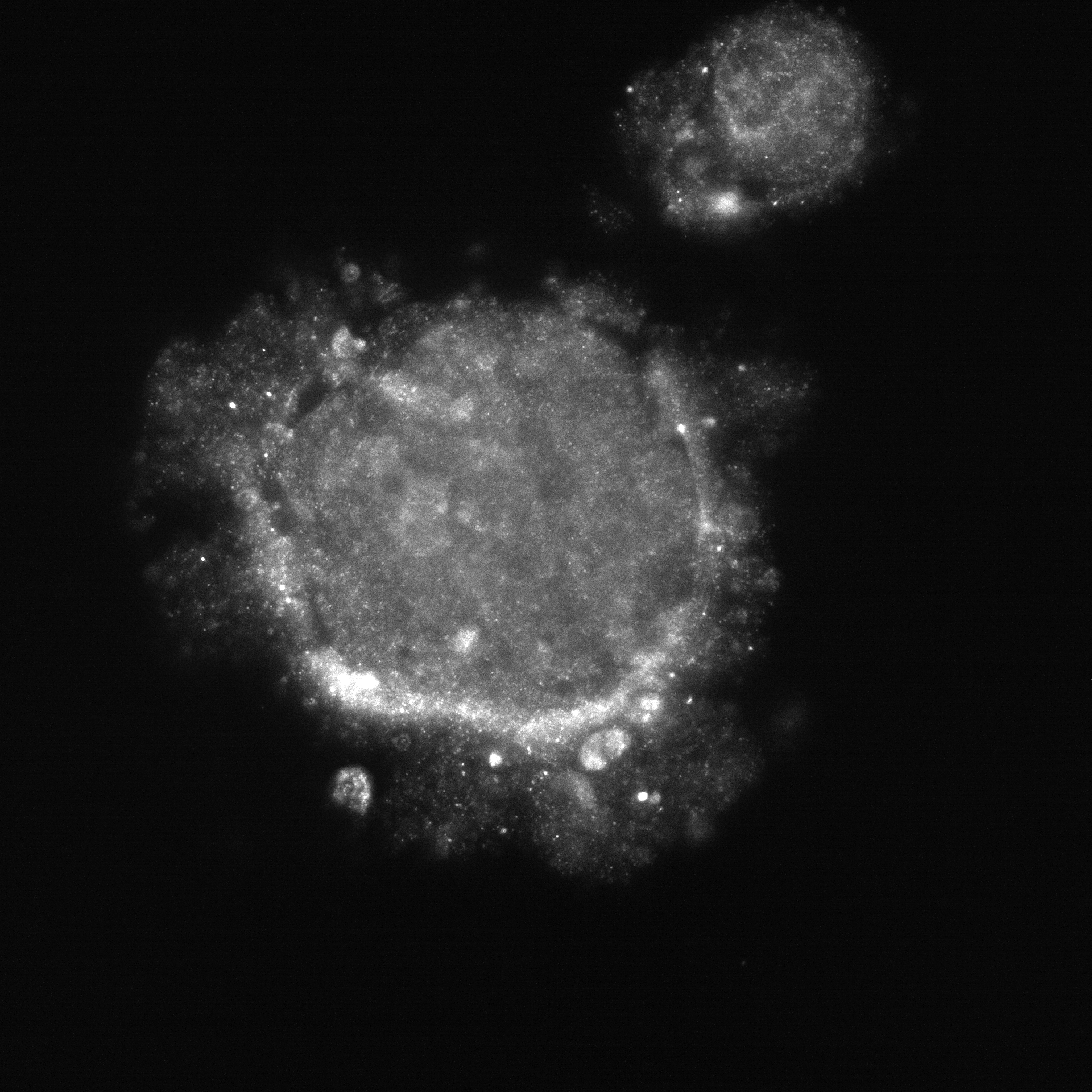

Supplement: Supplementary file 13 — Movie EV2 [file 44321_2025_289_MOESM13_ESM.zip › EMM-2025-21514_SourceData_Figure 3/3B/PDOs-Pat.6_Nao-3.tif]

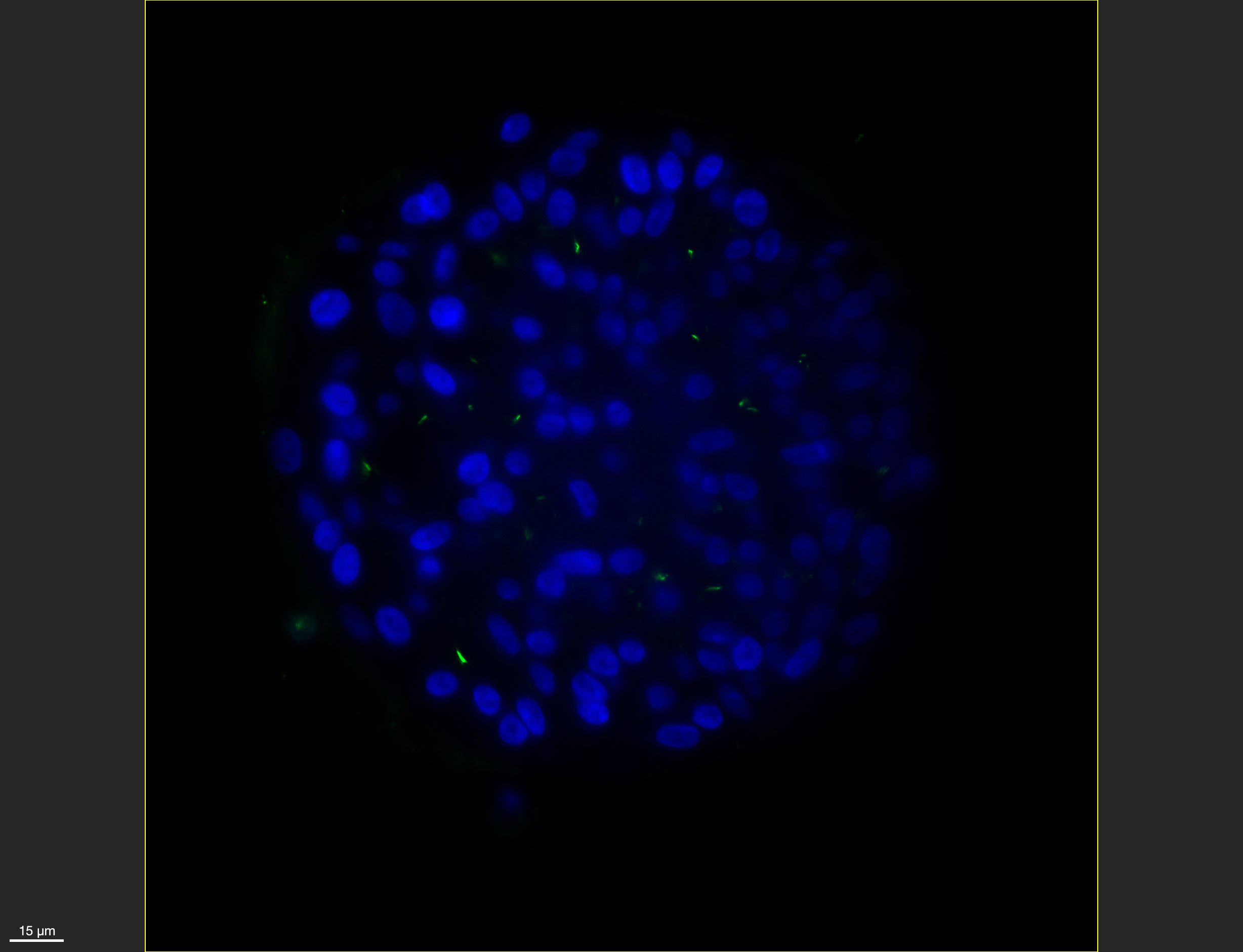

Supplement: Supplementary file 13 — Movie EV2 [file 44321_2025_289_MOESM13_ESM.zip › EMM-2025-21514_SourceData_Figure 3/3D/PDOs-Pat.6_0.001uM.tif]

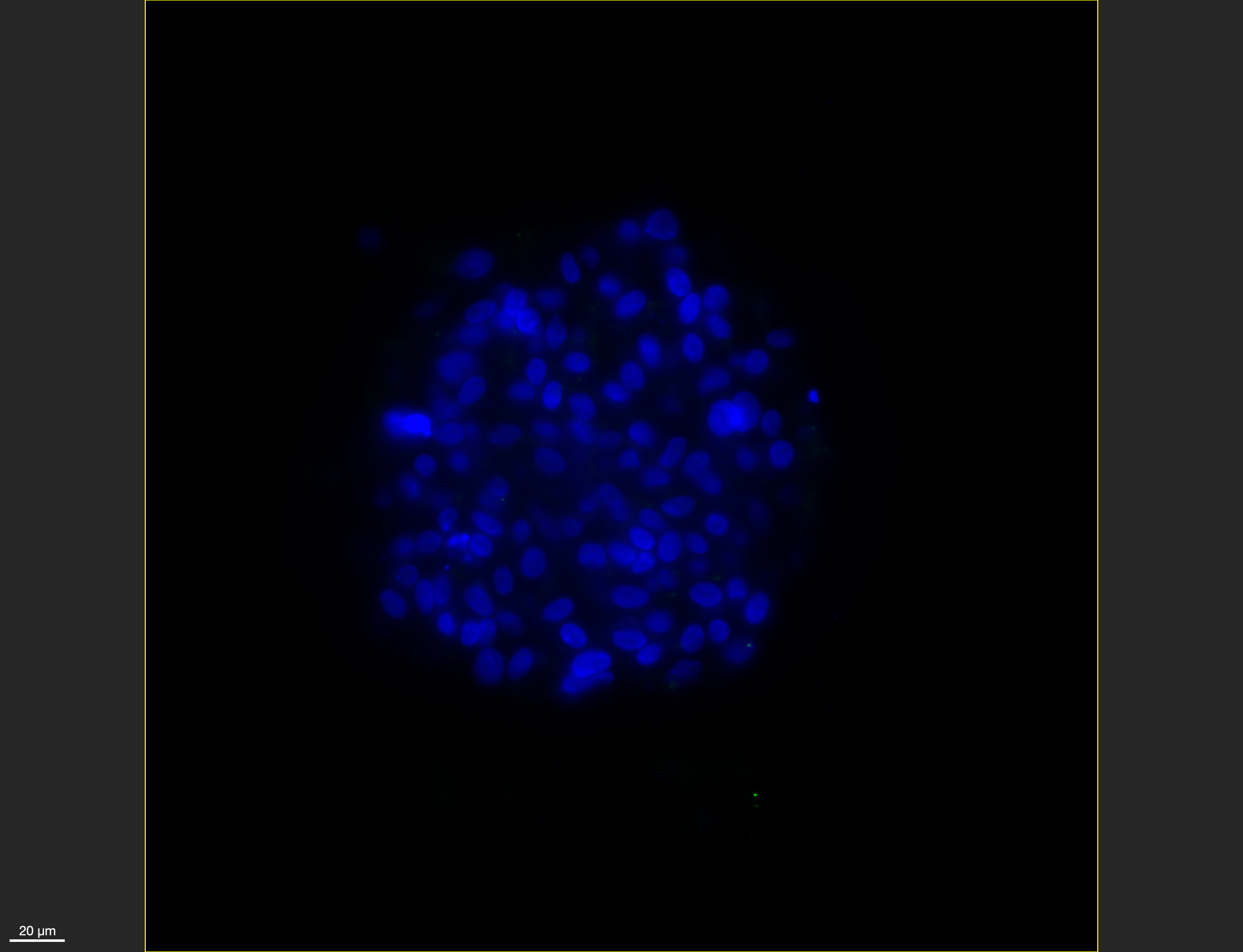

Supplement: Supplementary file 13 — Movie EV2 [file 44321_2025_289_MOESM13_ESM.zip › EMM-2025-21514_SourceData_Figure 3/3D/PDOs-Pat.6_10uM.tif]

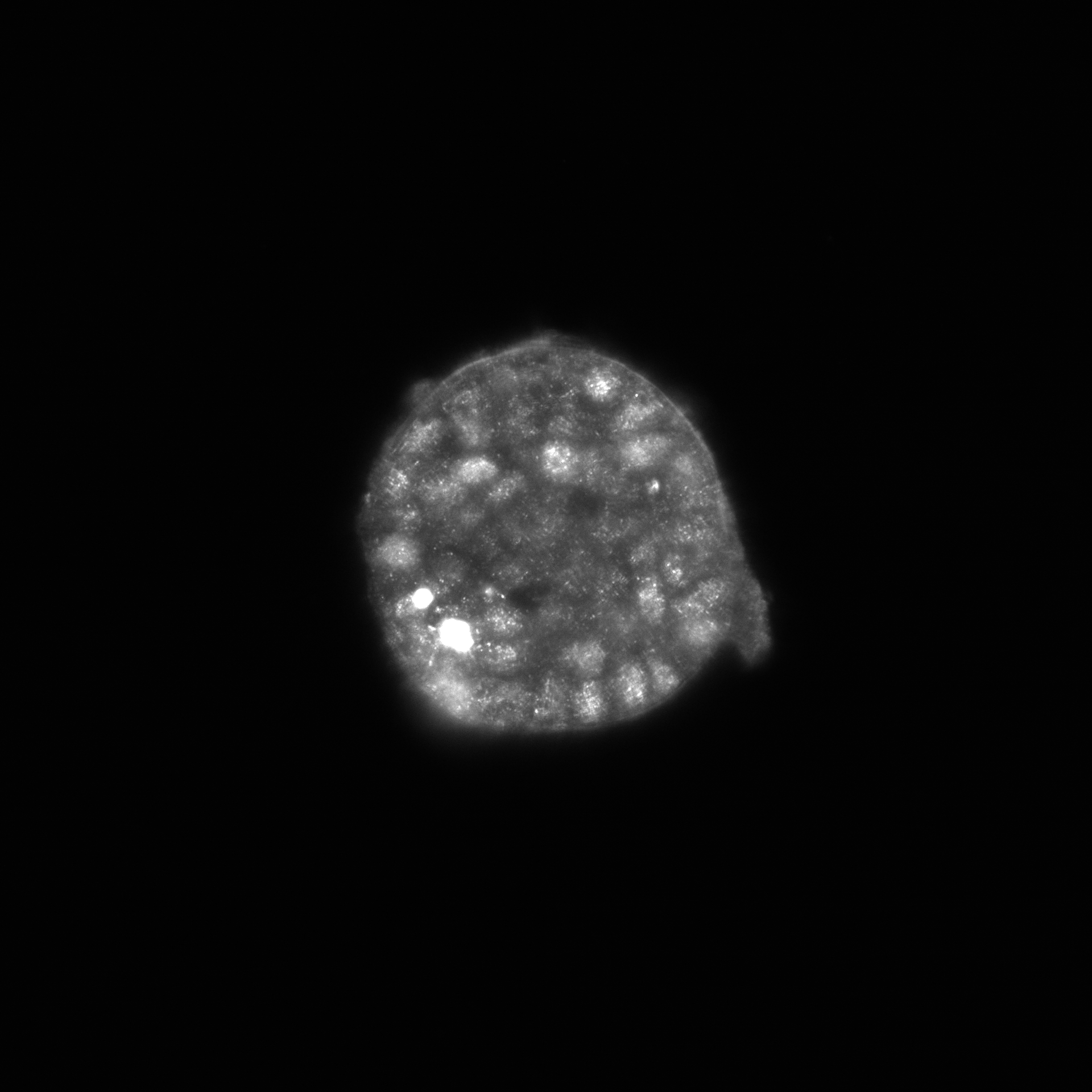

Supplement: Supplementary file 13 — Movie EV2 [file 44321_2025_289_MOESM13_ESM.zip › EMM-2025-21514_SourceData_Figure 3/3F/PDO_Pat.7_CTL.tif]

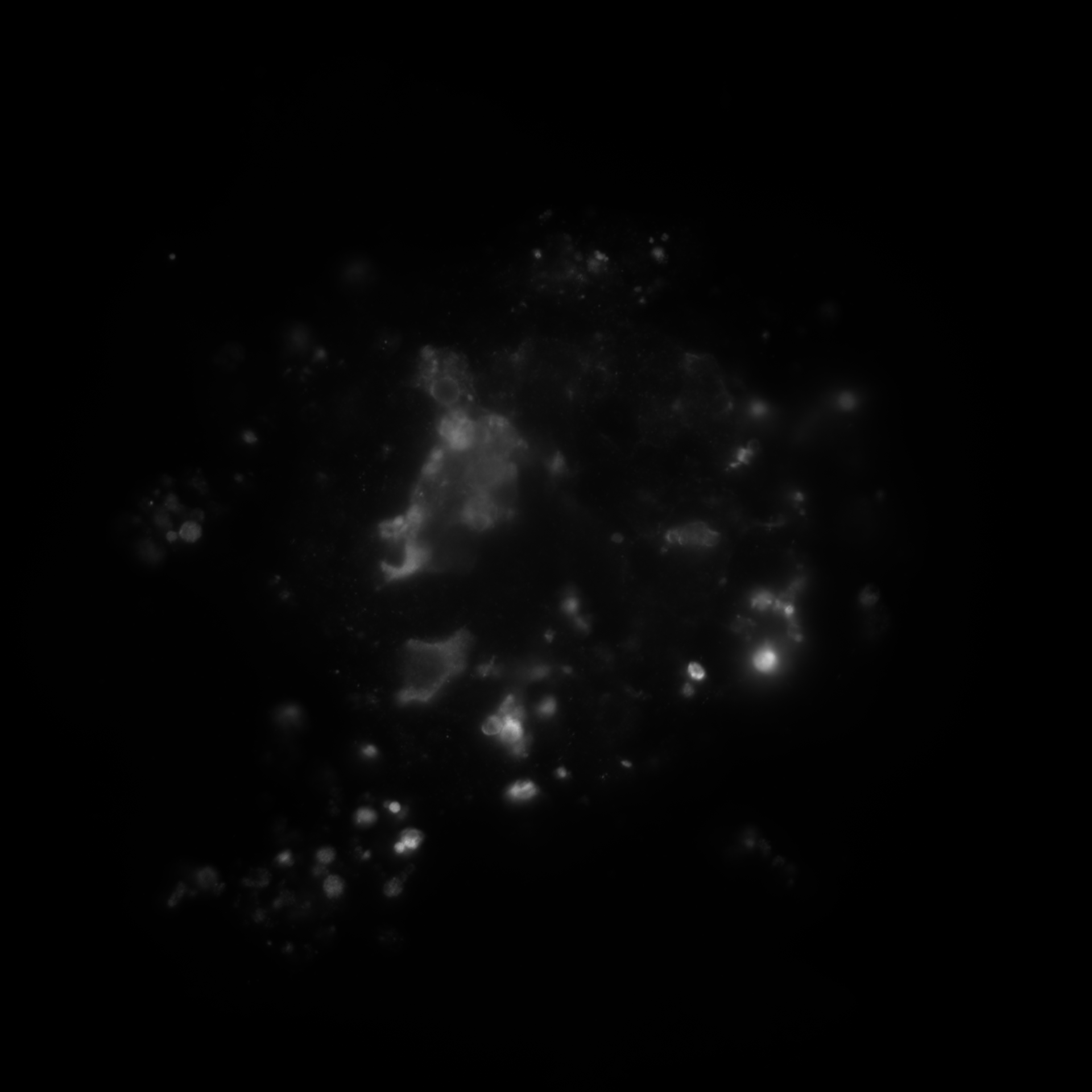

Supplement: Supplementary file 13 — Movie EV2 [file 44321_2025_289_MOESM13_ESM.zip › EMM-2025-21514_SourceData_Figure 3/3F/PDO_Pat.6_Taxol+Nao-3.tif]

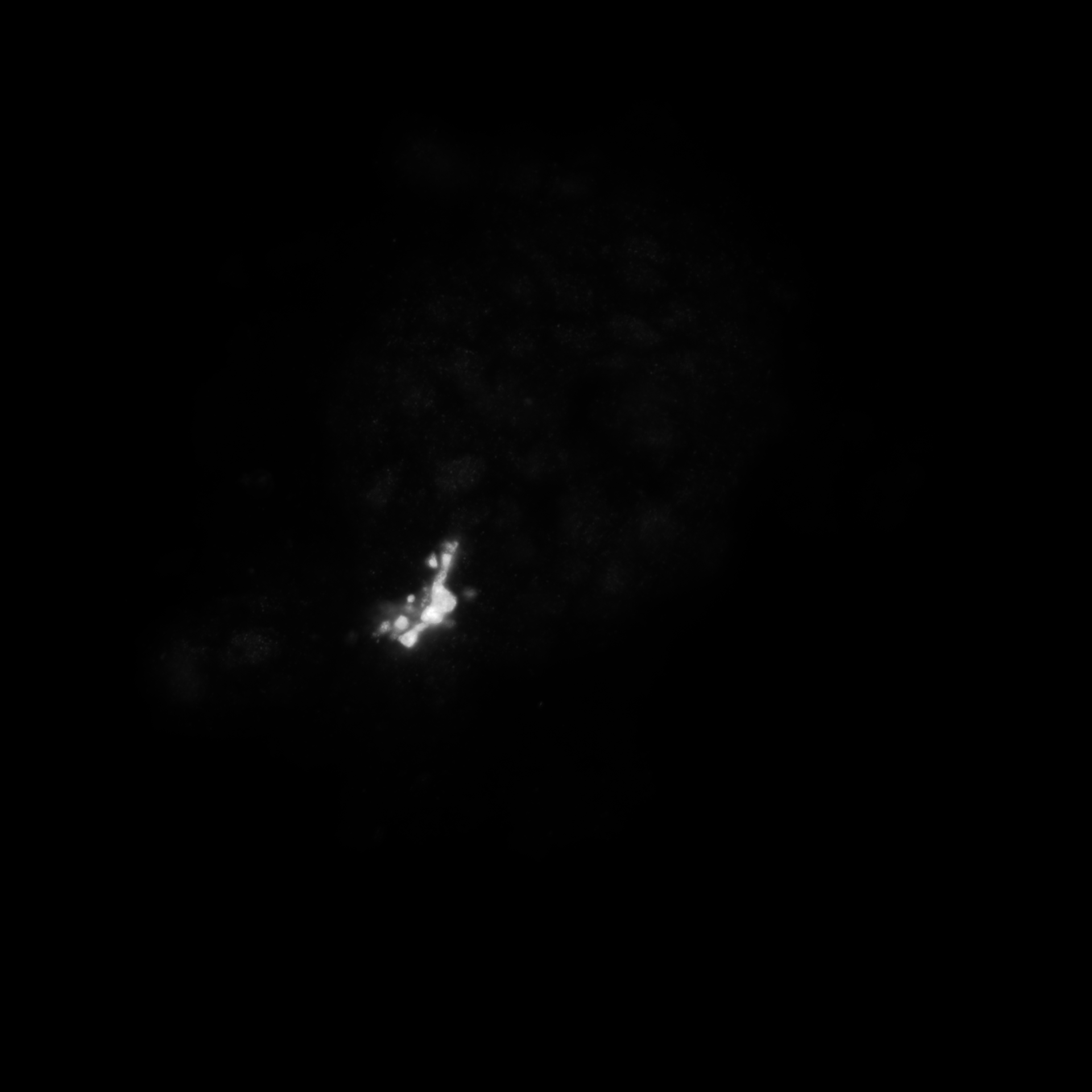

Supplement: Supplementary file 13 — Movie EV2 [file 44321_2025_289_MOESM13_ESM.zip › EMM-2025-21514_SourceData_Figure 3/3F/PDO_Pat.6_CTL.tif]

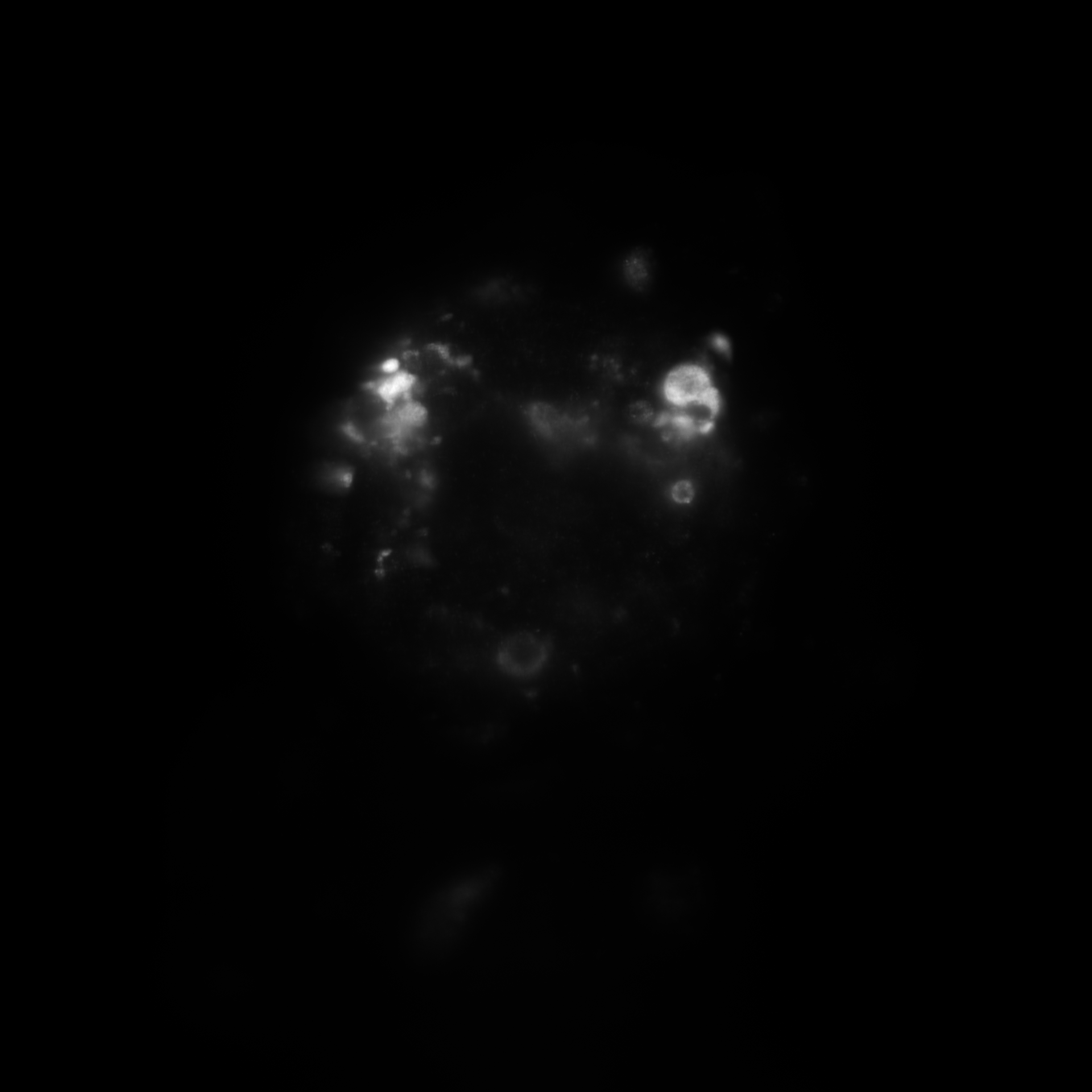

Supplement: Supplementary file 13 — Movie EV2 [file 44321_2025_289_MOESM13_ESM.zip › EMM-2025-21514_SourceData_Figure 3/3F/PDO_Pat.6_Taxol.tif]

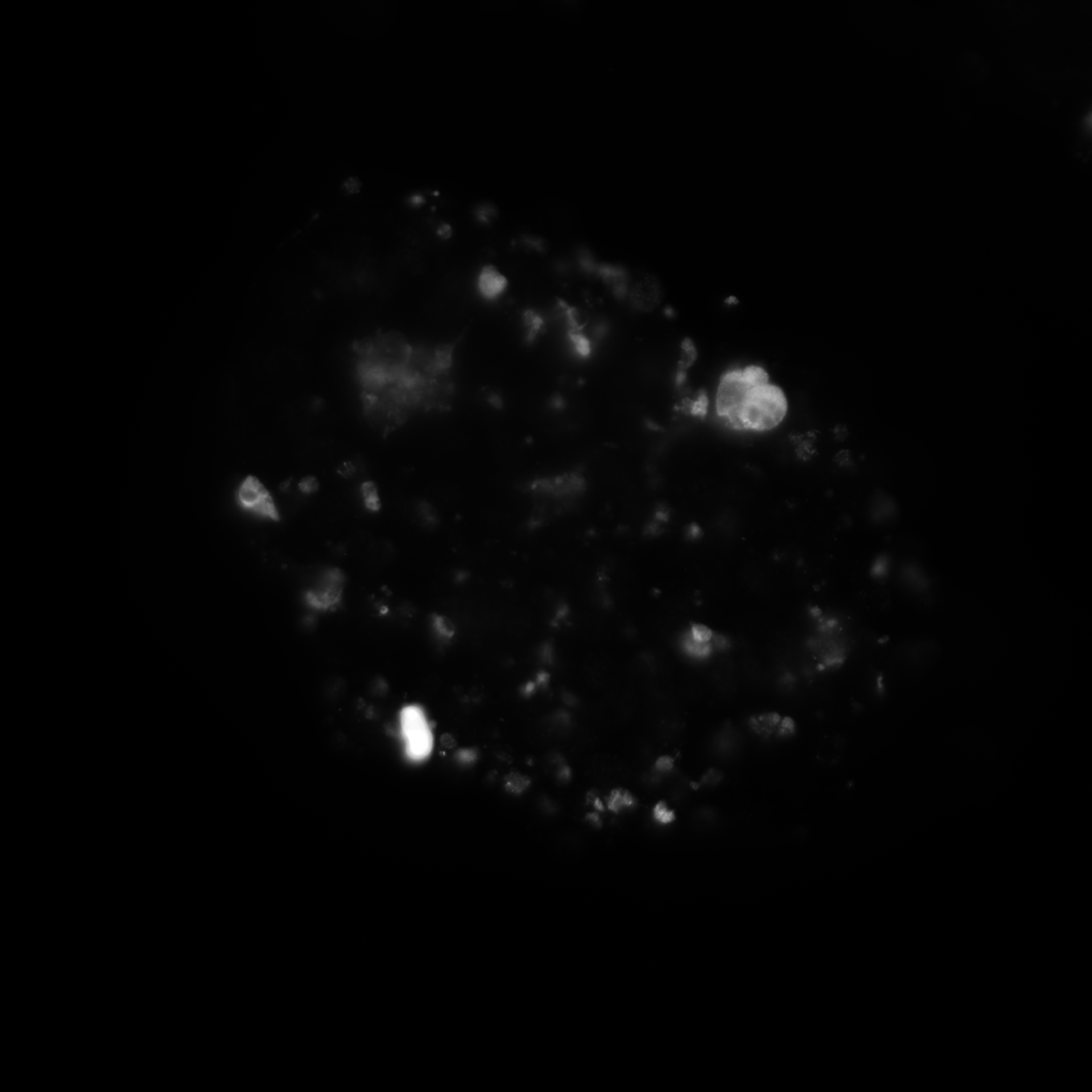

Supplement: Supplementary file 13 — Movie EV2 [file 44321_2025_289_MOESM13_ESM.zip › EMM-2025-21514_SourceData_Figure 3/3F/PDO_Pat.7_Taxol+Nao-3.tif]

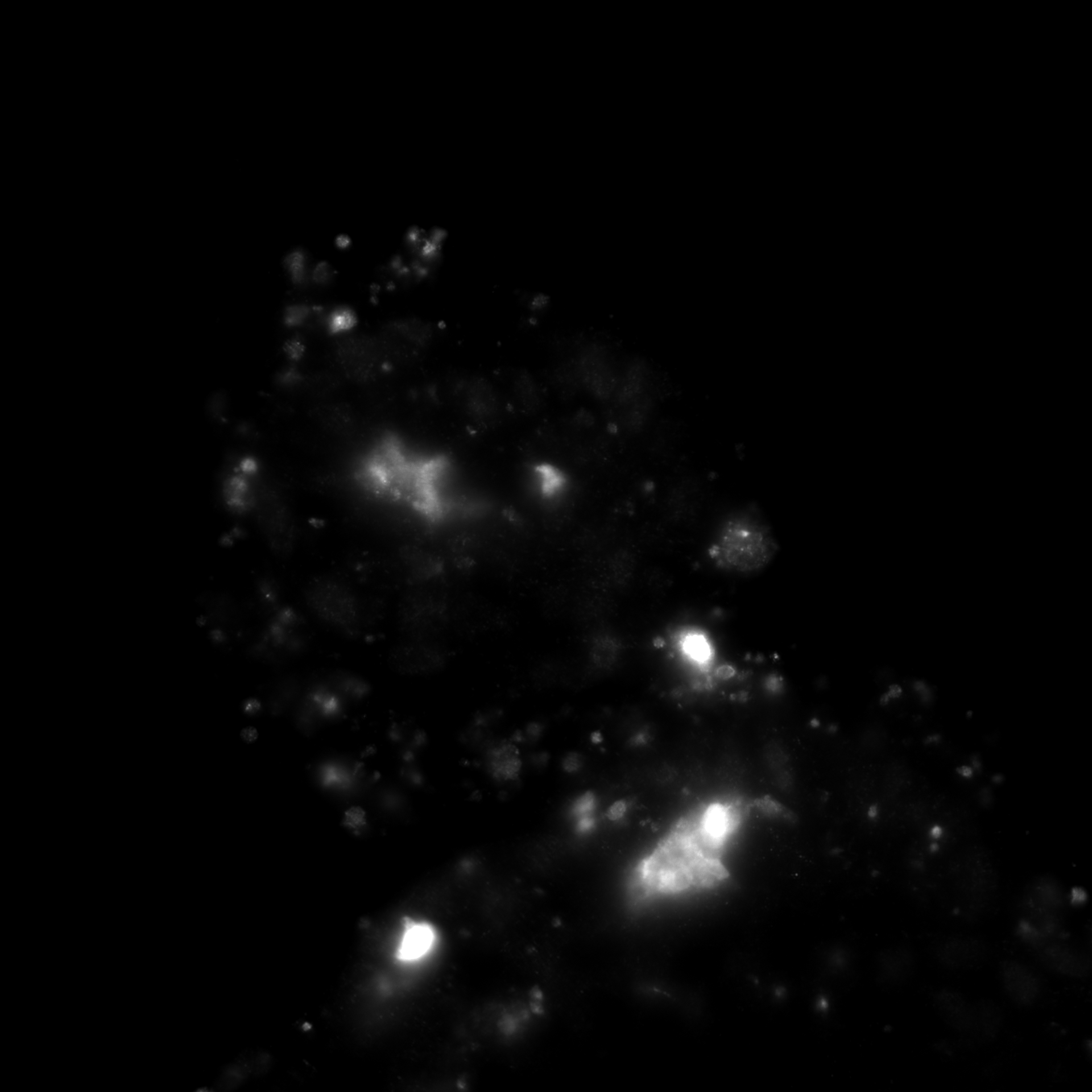

Supplement: Supplementary file 13 — Movie EV2 [file 44321_2025_289_MOESM13_ESM.zip › EMM-2025-21514_SourceData_Figure 3/3F/PDO_Pat.3_Nao-3.tif]

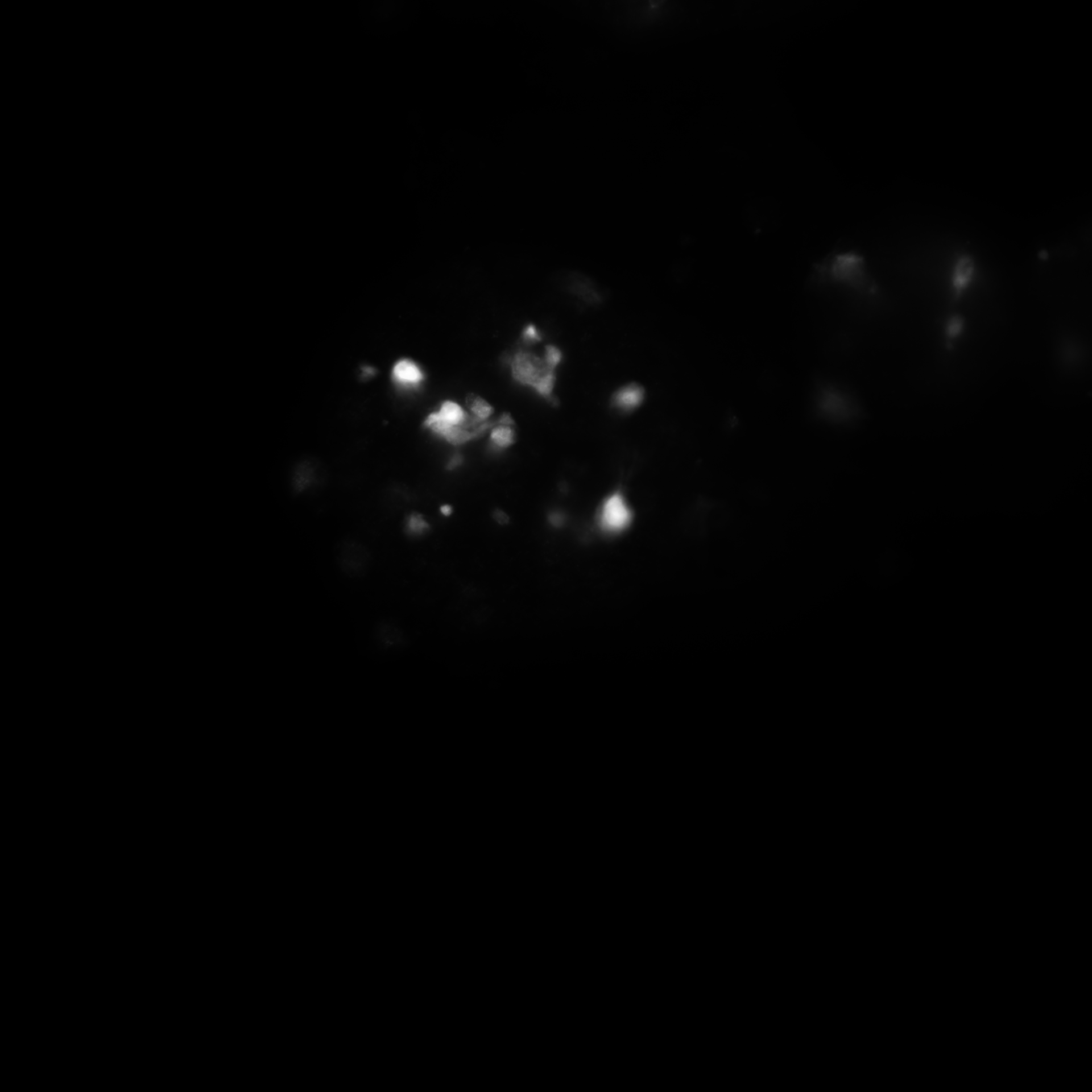

Supplement: Supplementary file 13 — Movie EV2 [file 44321_2025_289_MOESM13_ESM.zip › EMM-2025-21514_SourceData_Figure 3/3F/PDO_Pat.7_Taxol.tif]

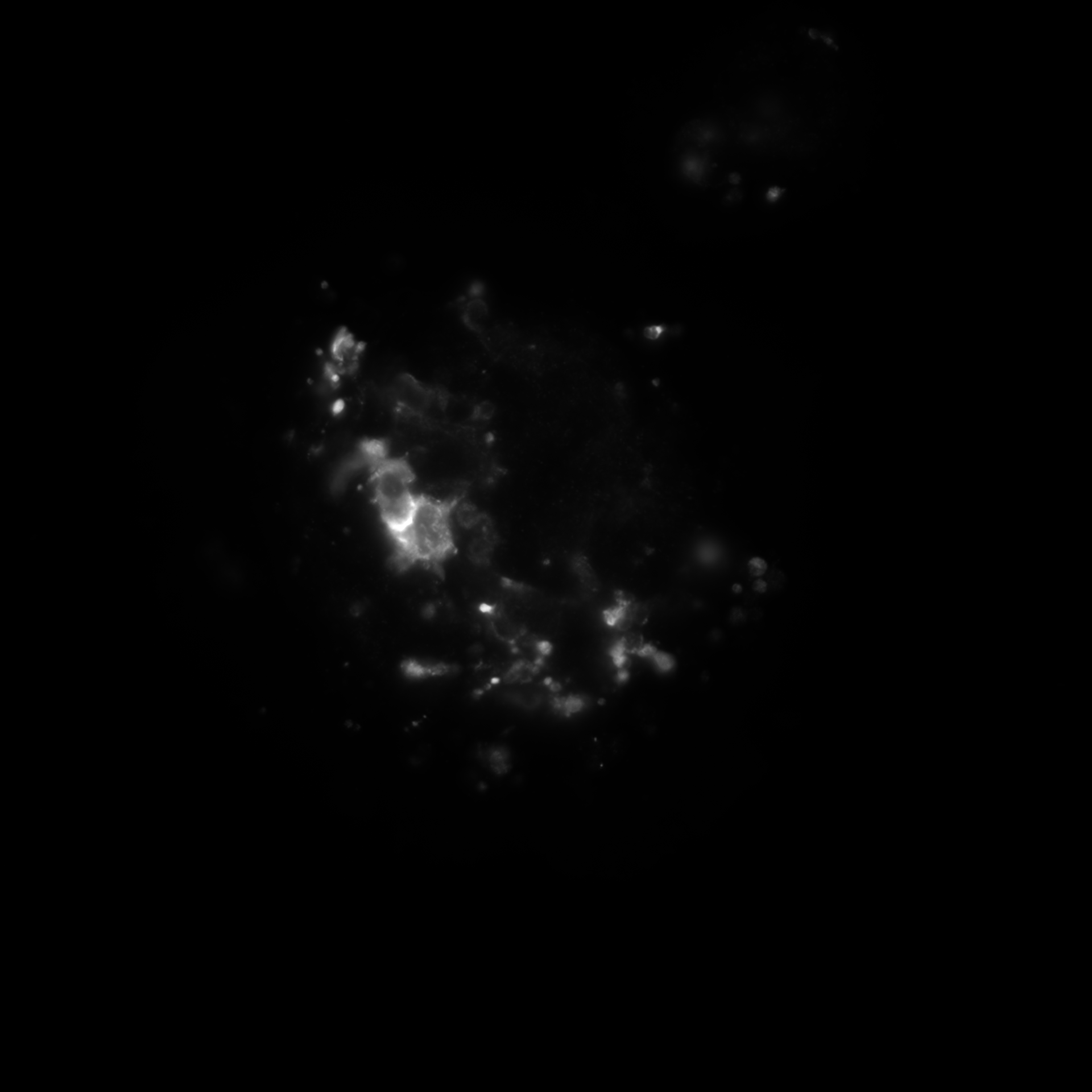

Supplement: Supplementary file 13 — Movie EV2 [file 44321_2025_289_MOESM13_ESM.zip › EMM-2025-21514_SourceData_Figure 3/3F/PDO_Pat.6_Nao-3.tif]

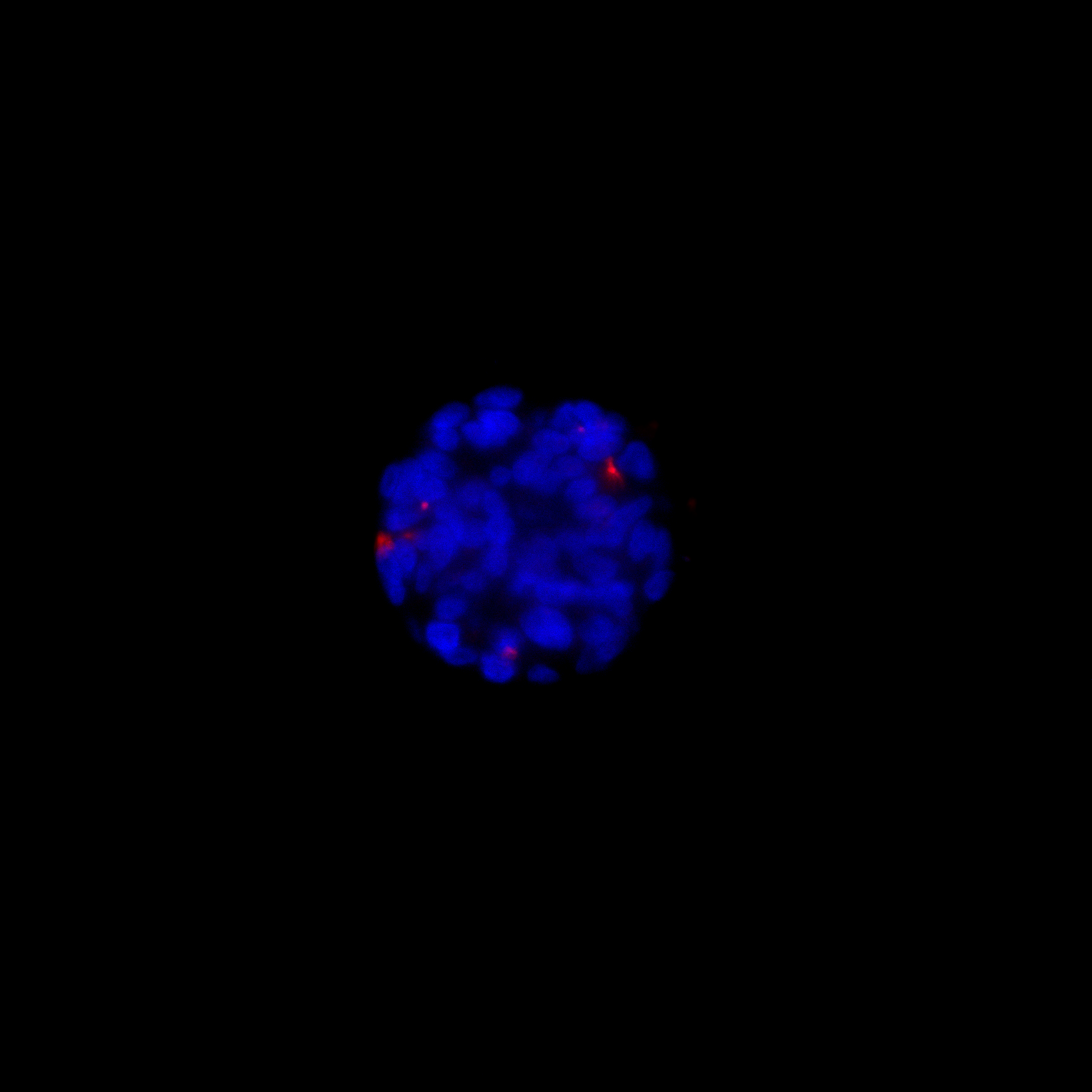

Supplement: Supplementary file 13 — Movie EV2 [file 44321_2025_289_MOESM13_ESM.zip › EMM-2025-21514_SourceData_Figure 3/3F/PDO_Pat.3_CTL.tif]

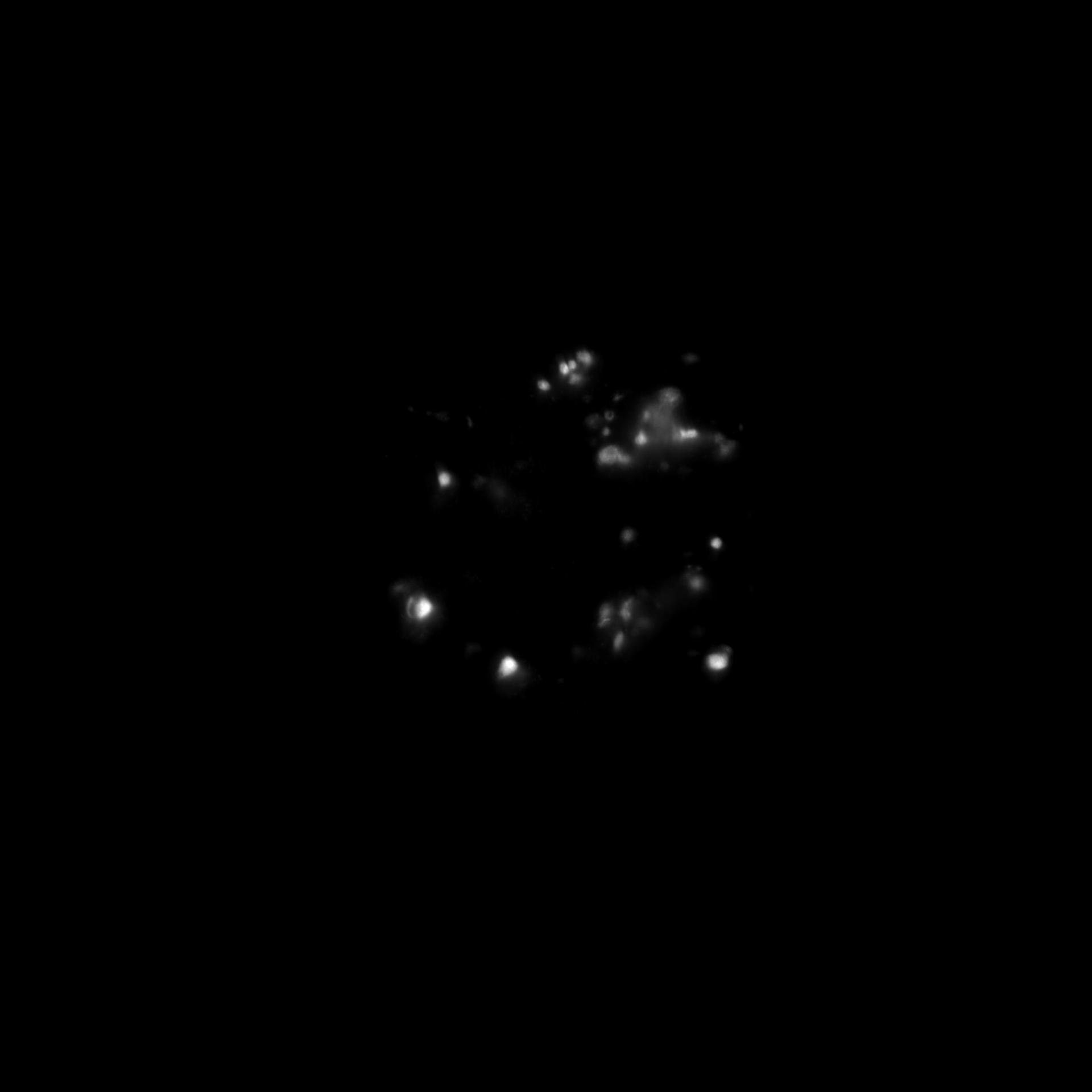

Supplement: Supplementary file 13 — Movie EV2 [file 44321_2025_289_MOESM13_ESM.zip › EMM-2025-21514_SourceData_Figure 3/3F/PDO_Pat.3_Taxol.tif]

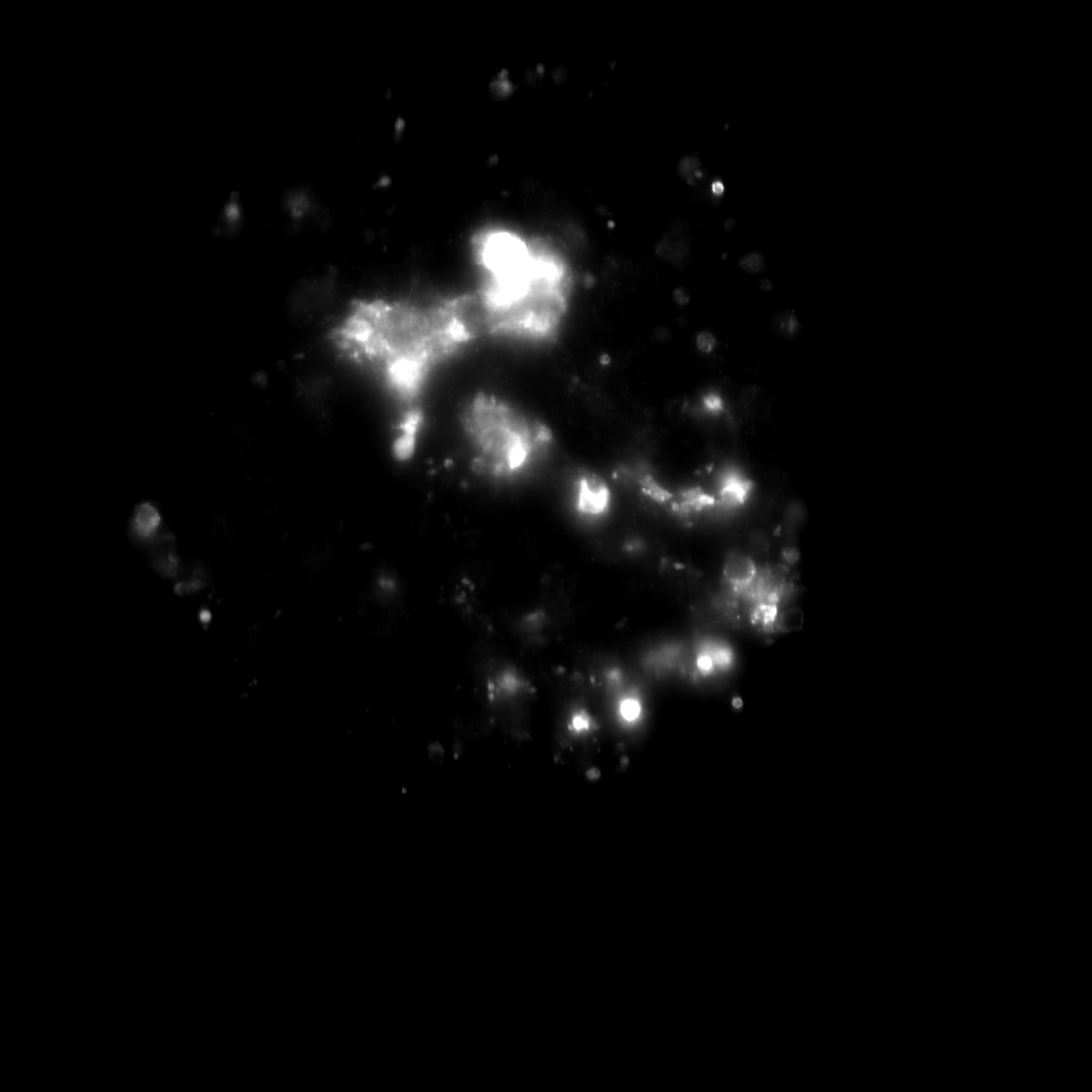

Supplement: Supplementary file 13 — Movie EV2 [file 44321_2025_289_MOESM13_ESM.zip › EMM-2025-21514_SourceData_Figure 3/3F/PDO_Pat.3_Tax+Nao-3.tif]

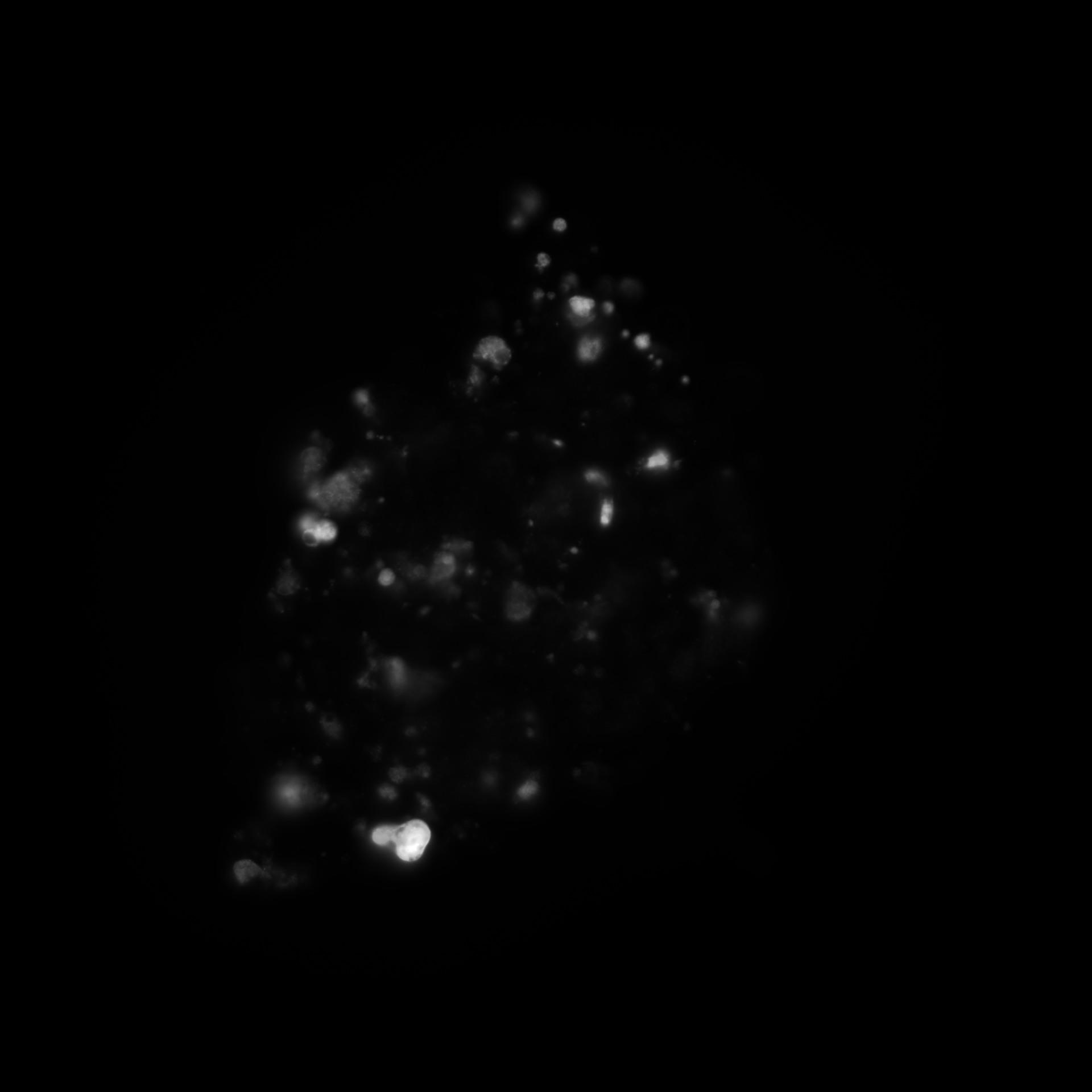

Supplement: Supplementary file 13 — Movie EV2 [file 44321_2025_289_MOESM13_ESM.zip › EMM-2025-21514_SourceData_Figure 3/3F/PDO_Pat.7_Nao-3.tif]

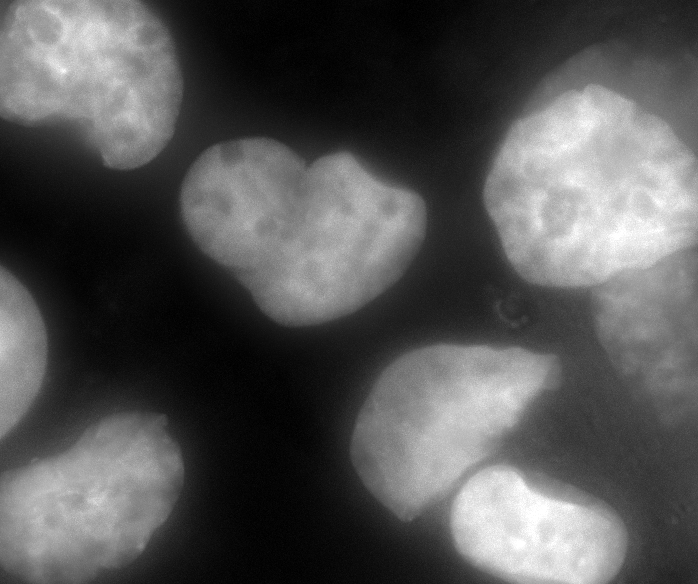

Supplement: Supplementary file 14 — Movie EV3 [file 44321_2025_289_MOESM14_ESM.zip › EMM-2025-21514_SourceData_Figure 4/4E/sgIFT20.tif]

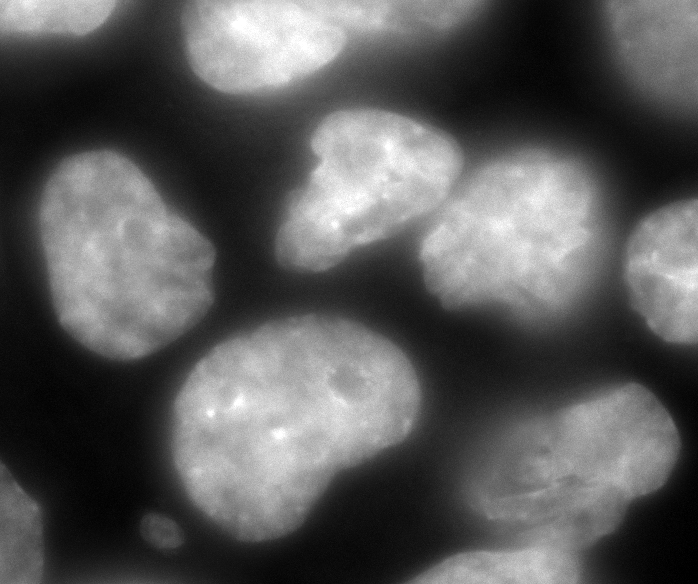

Supplement: Supplementary file 14 — Movie EV3 [file 44321_2025_289_MOESM14_ESM.zip › EMM-2025-21514_SourceData_Figure 4/4E/sgKif3a.tif]

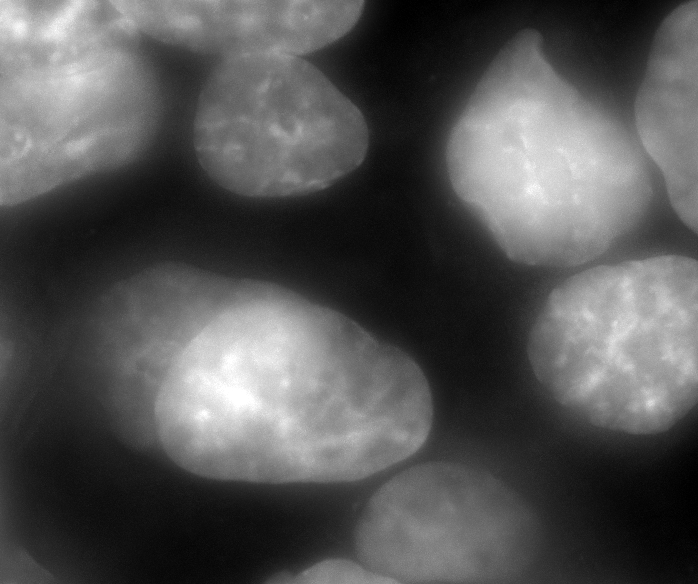

Supplement: Supplementary file 14 — Movie EV3 [file 44321_2025_289_MOESM14_ESM.zip › EMM-2025-21514_SourceData_Figure 4/4E/sgCTL.tif]

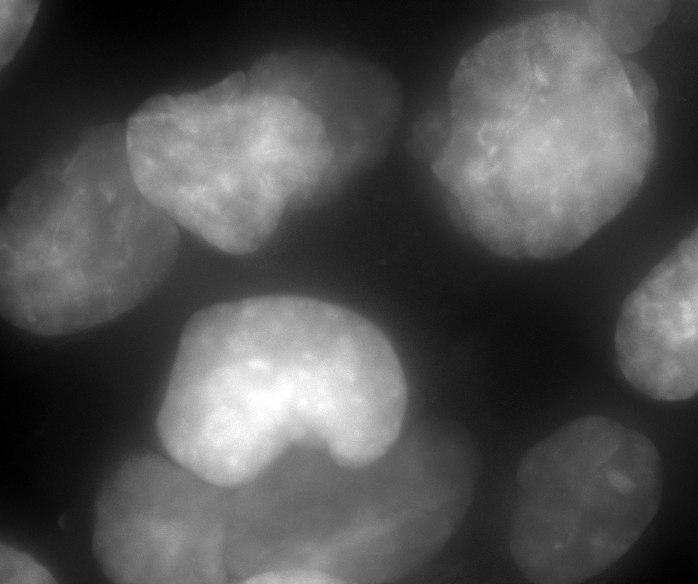

Supplement: Supplementary file 14 — Movie EV3 [file 44321_2025_289_MOESM14_ESM.zip › EMM-2025-21514_SourceData_Figure 4/4B/shEcad.tif]

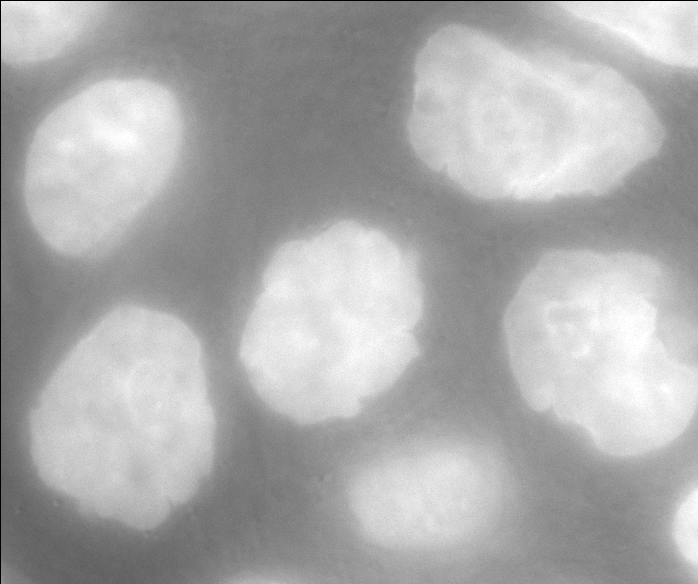

Supplement: Supplementary file 14 — Movie EV3 [file 44321_2025_289_MOESM14_ESM.zip › EMM-2025-21514_SourceData_Figure 4/4B/shCTL.tif]

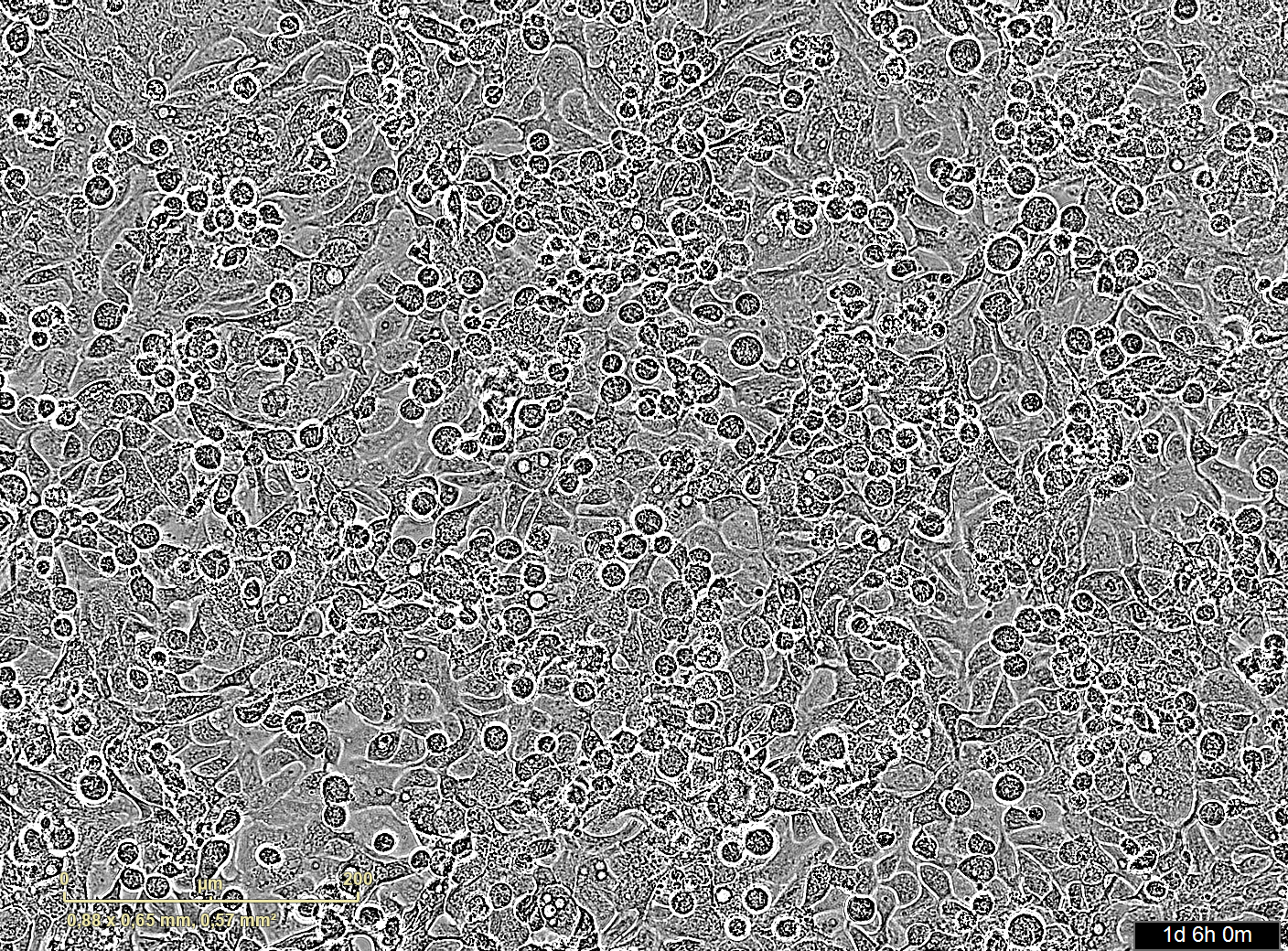

Supplement: Supplementary file 14 — Movie EV3 [file 44321_2025_289_MOESM14_ESM.zip › EMM-2025-21514_SourceData_Figure 4/4C/phase shECAD.tiff]

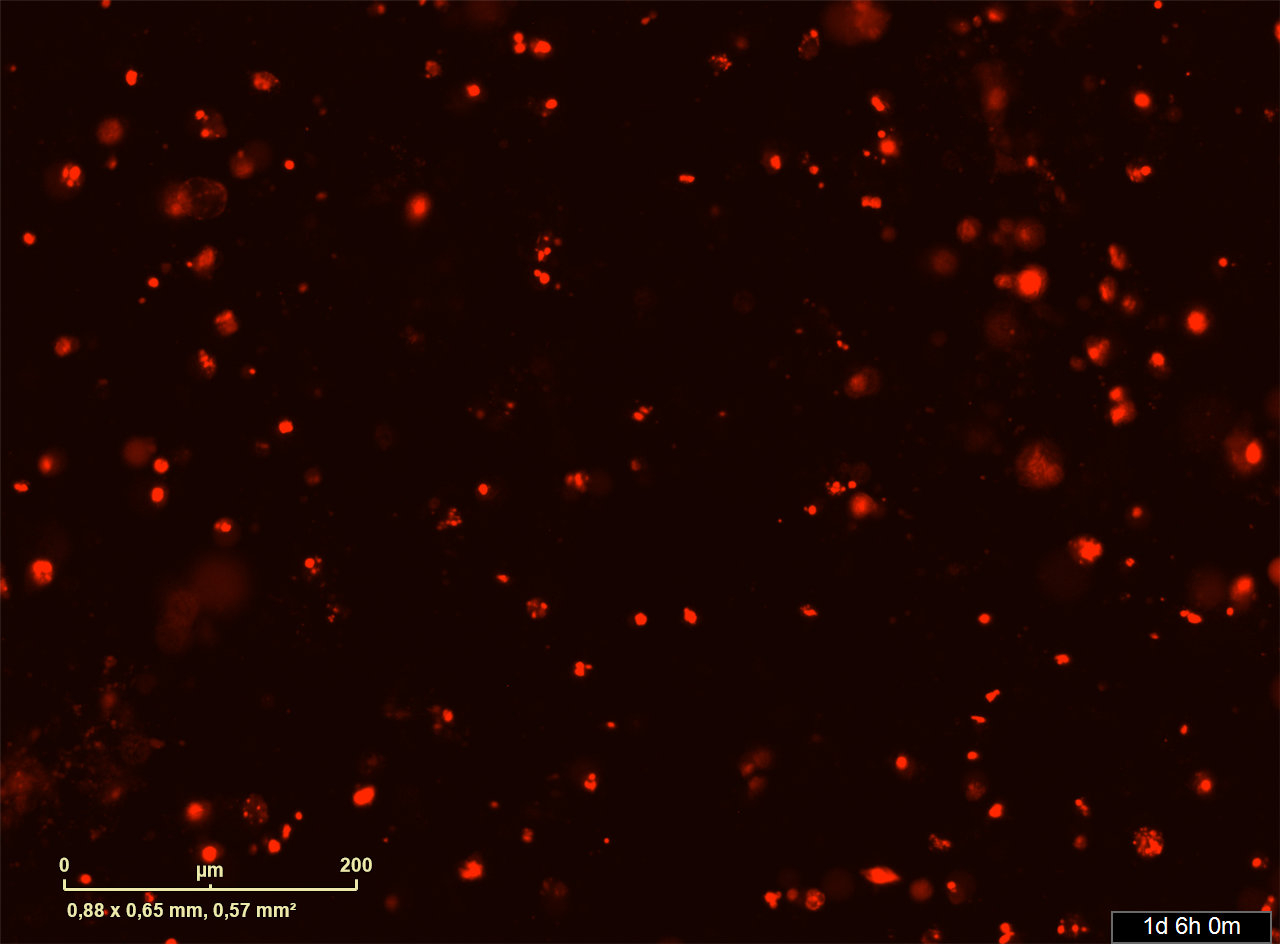

Supplement: Supplementary file 14 — Movie EV3 [file 44321_2025_289_MOESM14_ESM.zip › EMM-2025-21514_SourceData_Figure 4/4C/PI shCTL.tiff]

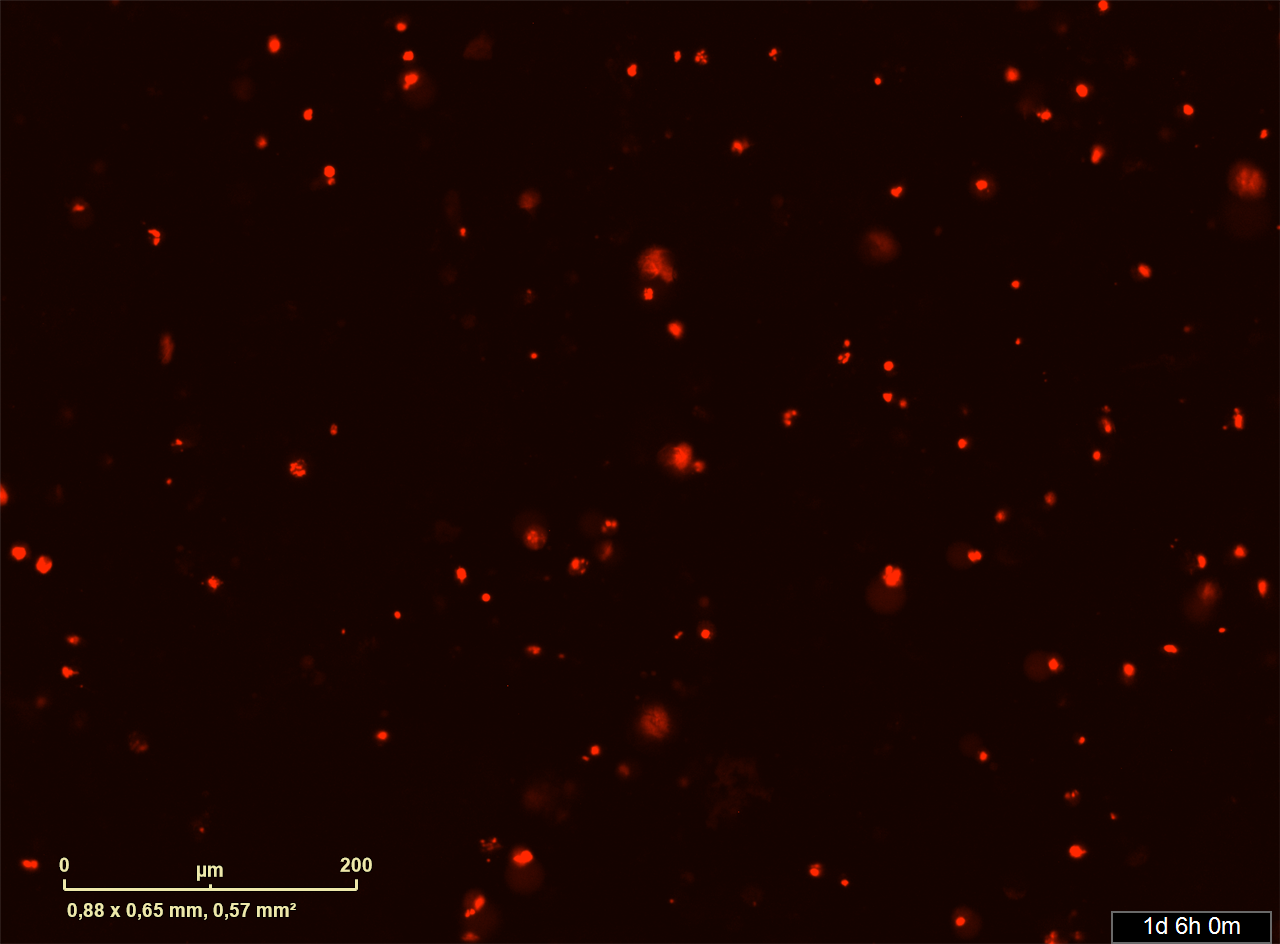

Supplement: Supplementary file 14 — Movie EV3 [file 44321_2025_289_MOESM14_ESM.zip › EMM-2025-21514_SourceData_Figure 4/4C/PI shECAD.tiff]

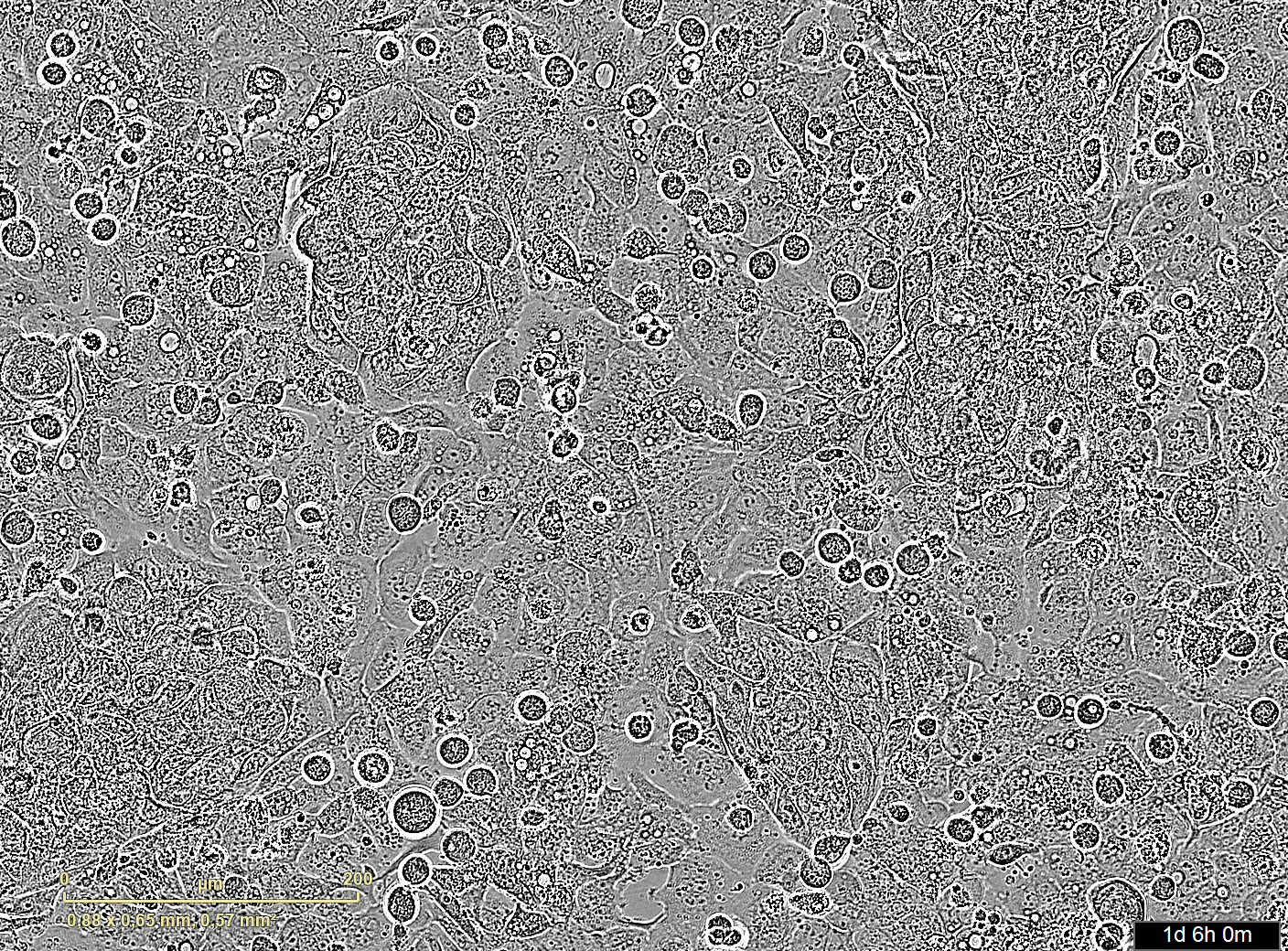

Supplement: Supplementary file 14 — Movie EV3 [file 44321_2025_289_MOESM14_ESM.zip › EMM-2025-21514_SourceData_Figure 4/4C/phase shCTL.tiff]

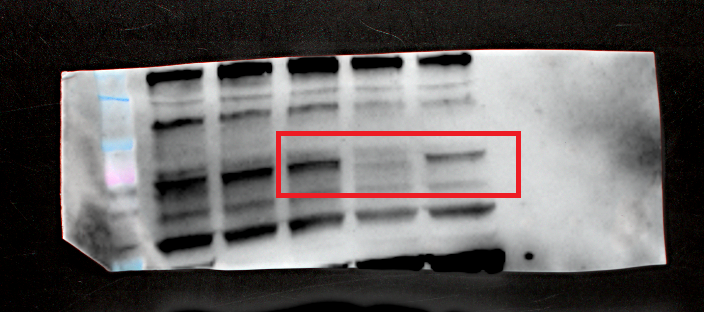

Supplement: Supplementary file 14 — Movie EV3 [file 44321_2025_289_MOESM14_ESM.zip › EMM-2025-21514_SourceData_Figure 4/4D/KIF3a.tif]

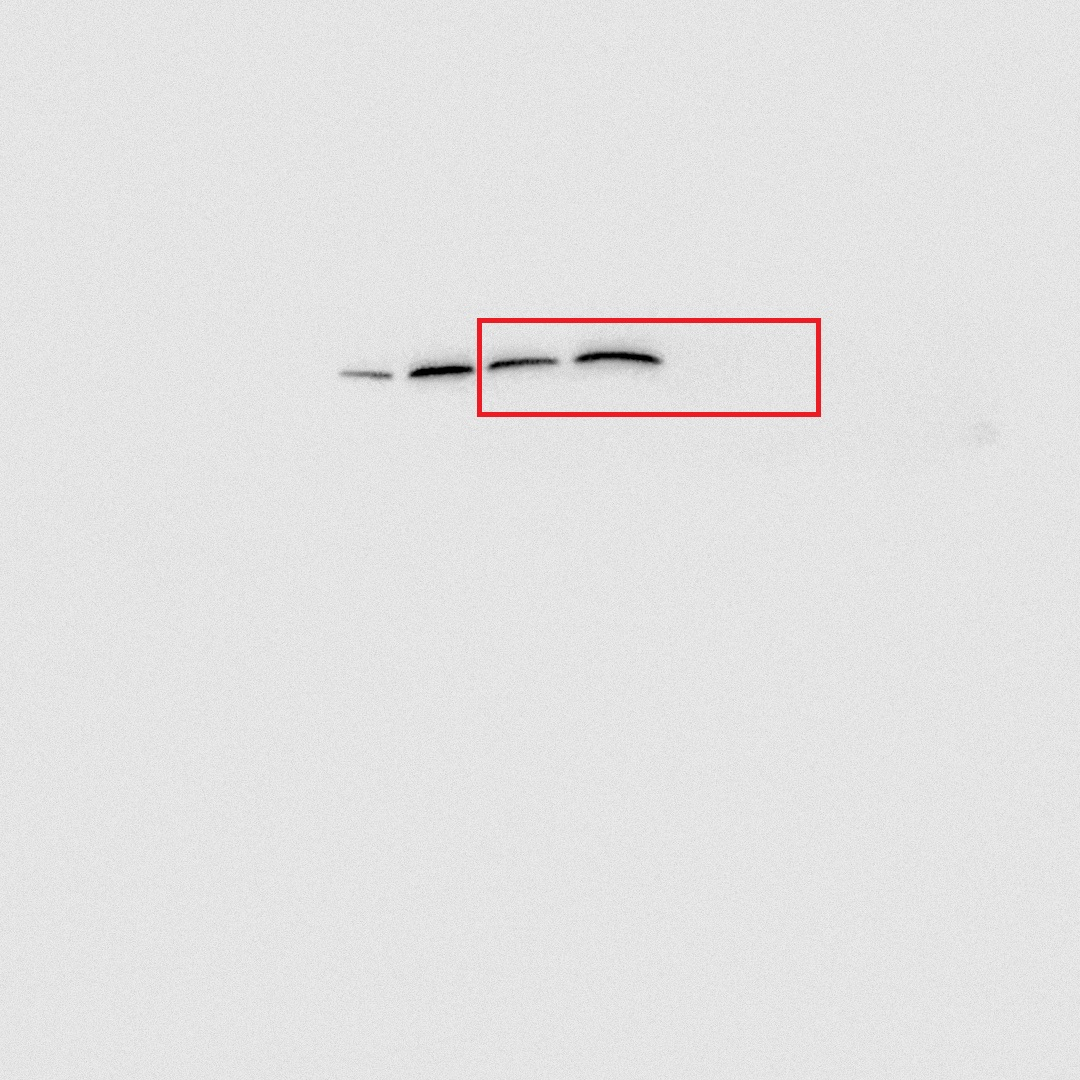

Supplement: Supplementary file 14 — Movie EV3 [file 44321_2025_289_MOESM14_ESM.zip › EMM-2025-21514_SourceData_Figure 4/4D/IFT20.tiff]

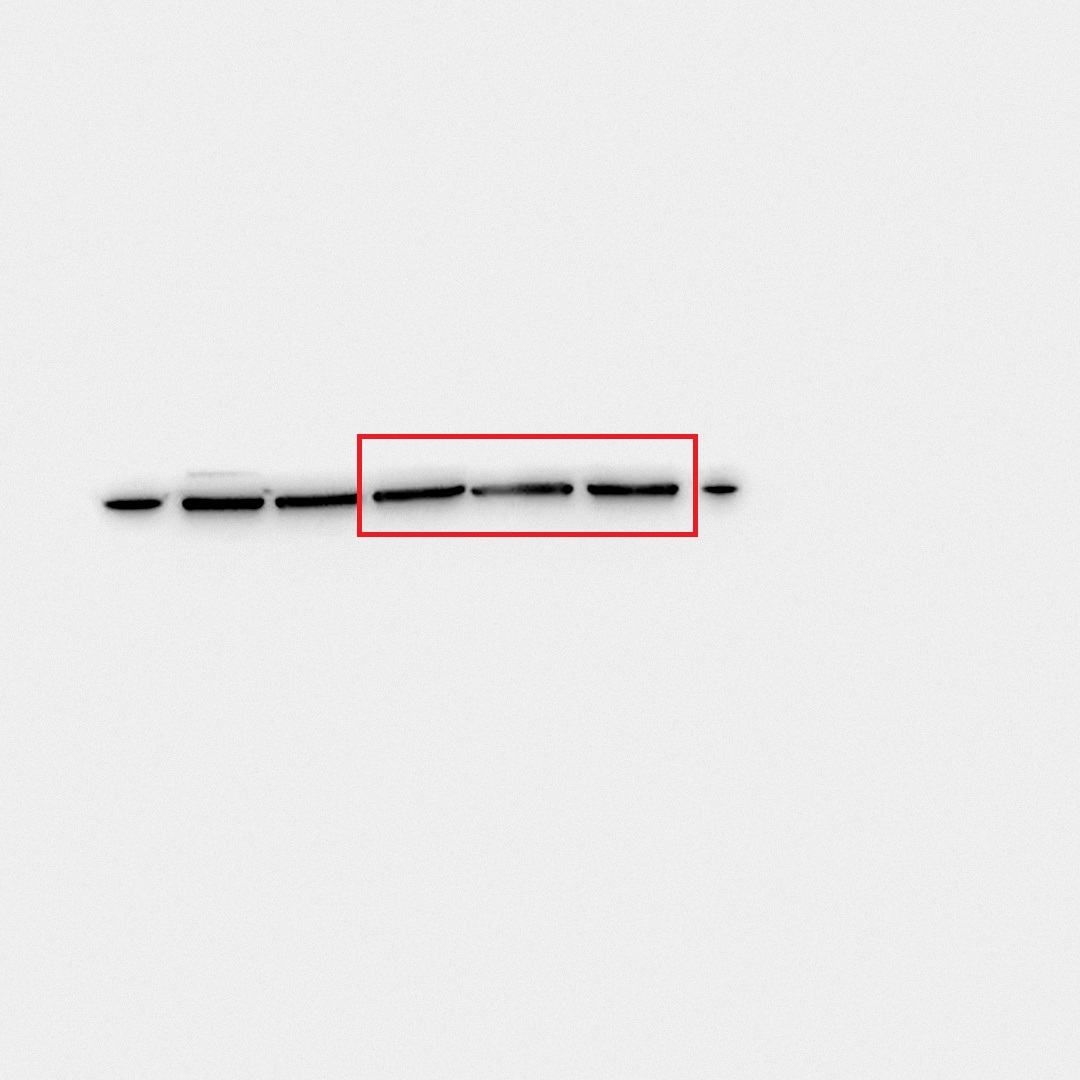

Supplement: Supplementary file 14 — Movie EV3 [file 44321_2025_289_MOESM14_ESM.zip › EMM-2025-21514_SourceData_Figure 4/4D/Actine.tiff]

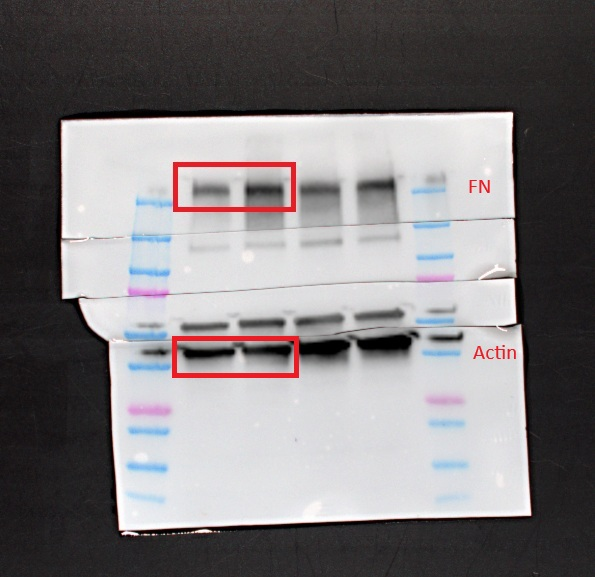

Supplement: Supplementary file 14 — Movie EV3 [file 44321_2025_289_MOESM14_ESM.zip › EMM-2025-21514_SourceData_Figure 4/4A/FN.tiff]

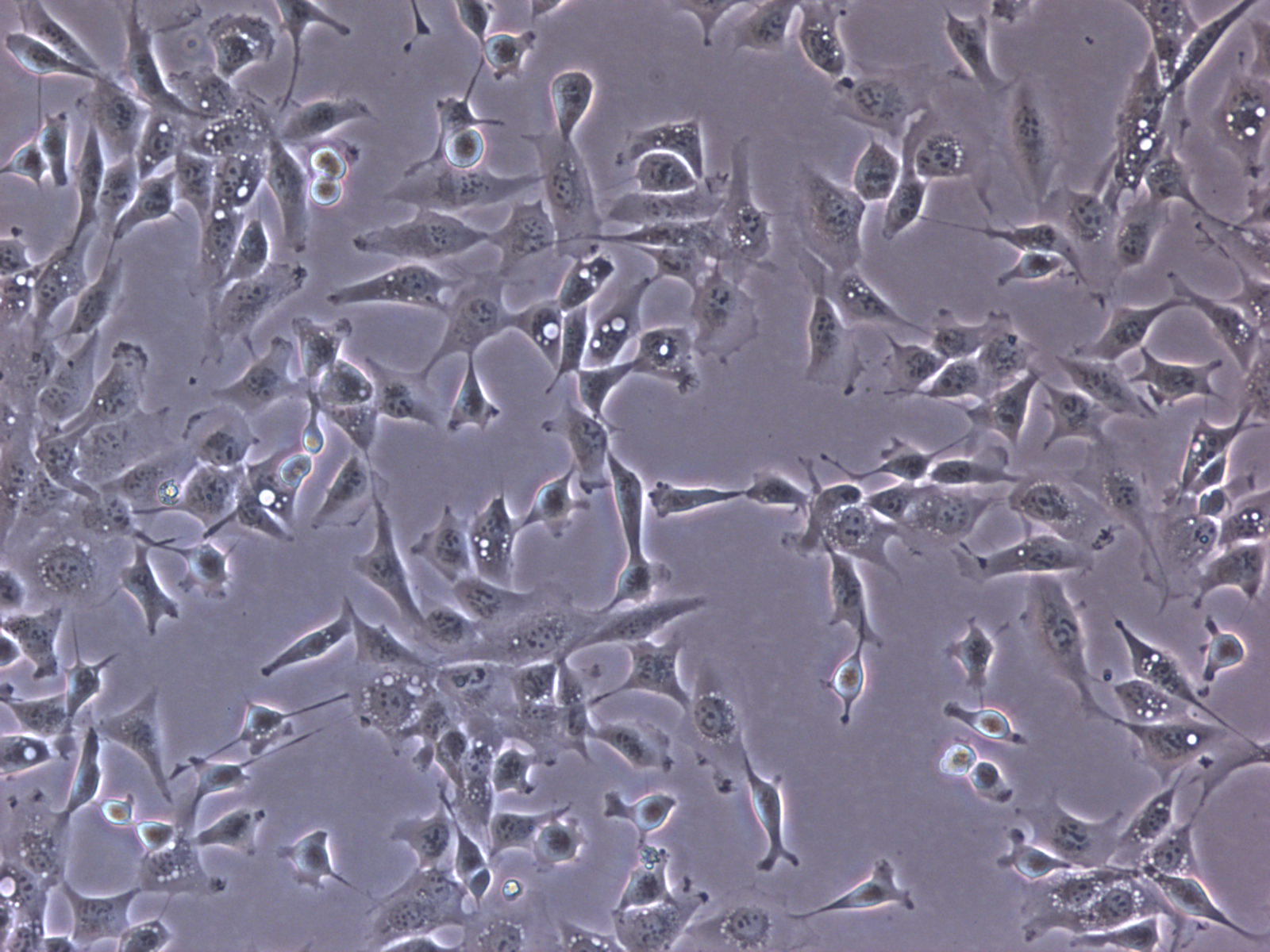

Supplement: Supplementary file 14 — Movie EV3 [file 44321_2025_289_MOESM14_ESM.zip › EMM-2025-21514_SourceData_Figure 4/4A/shEcad.tif]

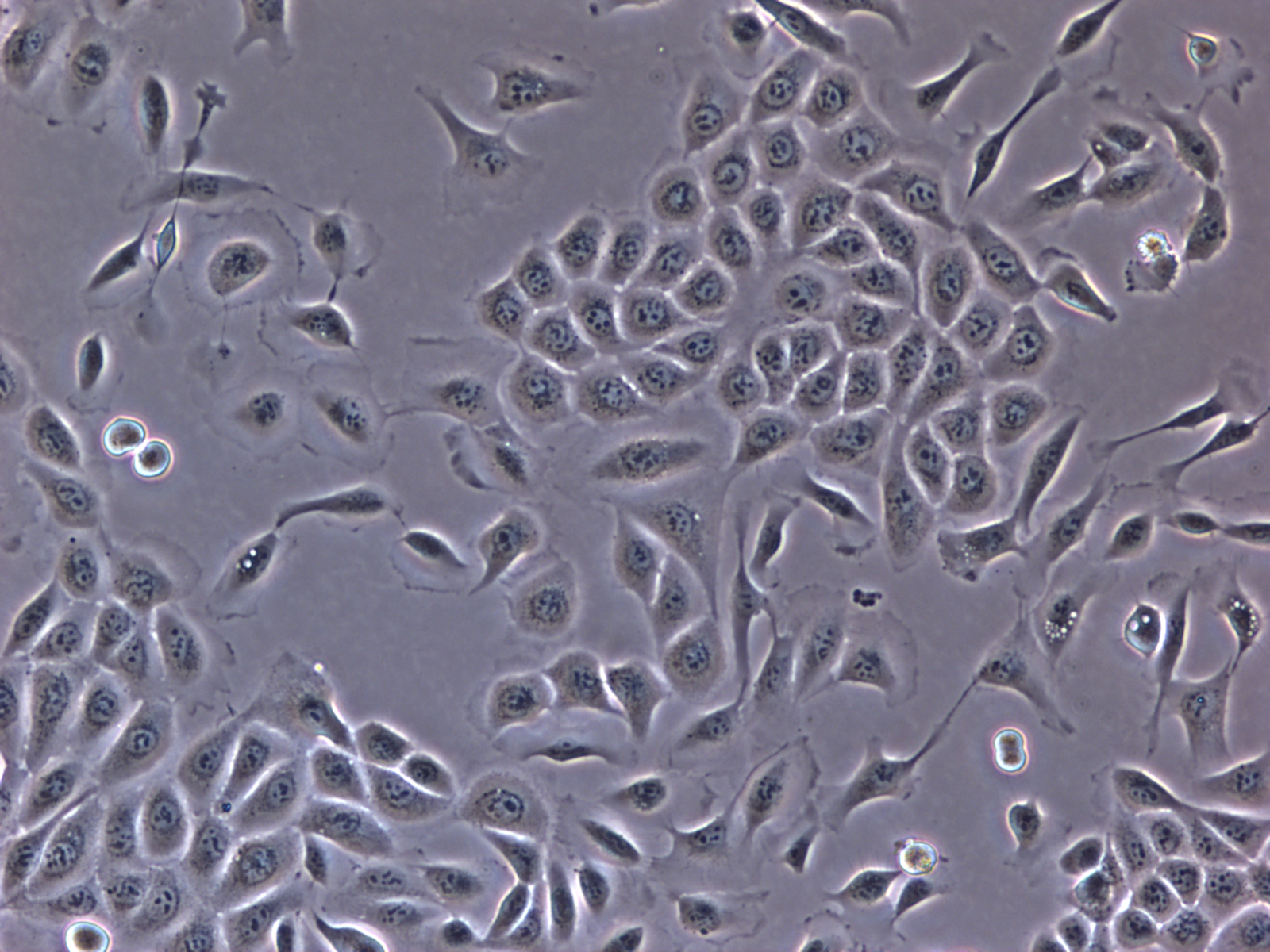

Supplement: Supplementary file 14 — Movie EV3 [file 44321_2025_289_MOESM14_ESM.zip › EMM-2025-21514_SourceData_Figure 4/4A/shCTL.tif]

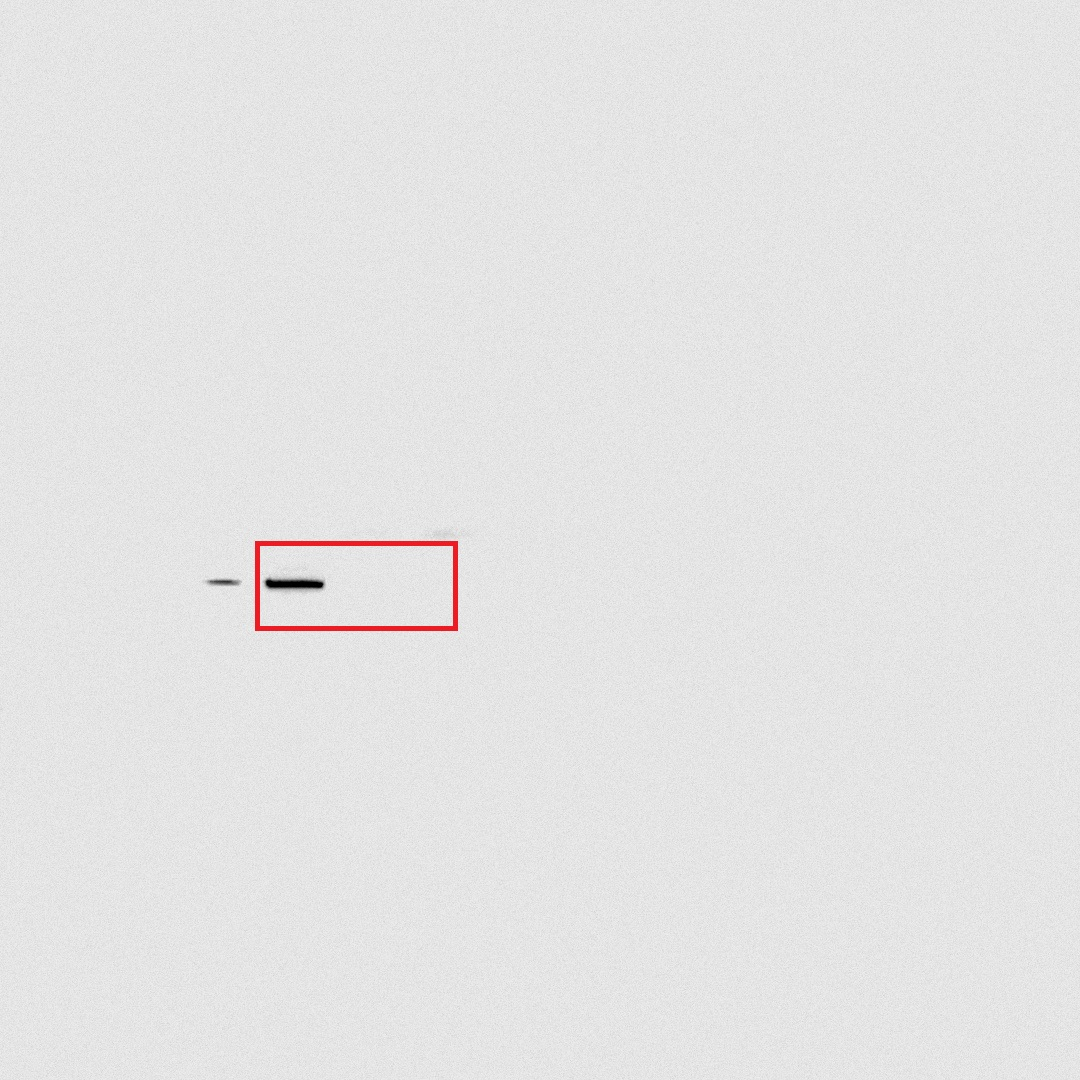

Supplement: Supplementary file 14 — Movie EV3 [file 44321_2025_289_MOESM14_ESM.zip › EMM-2025-21514_SourceData_Figure 4/4A/Ecad.tiff]

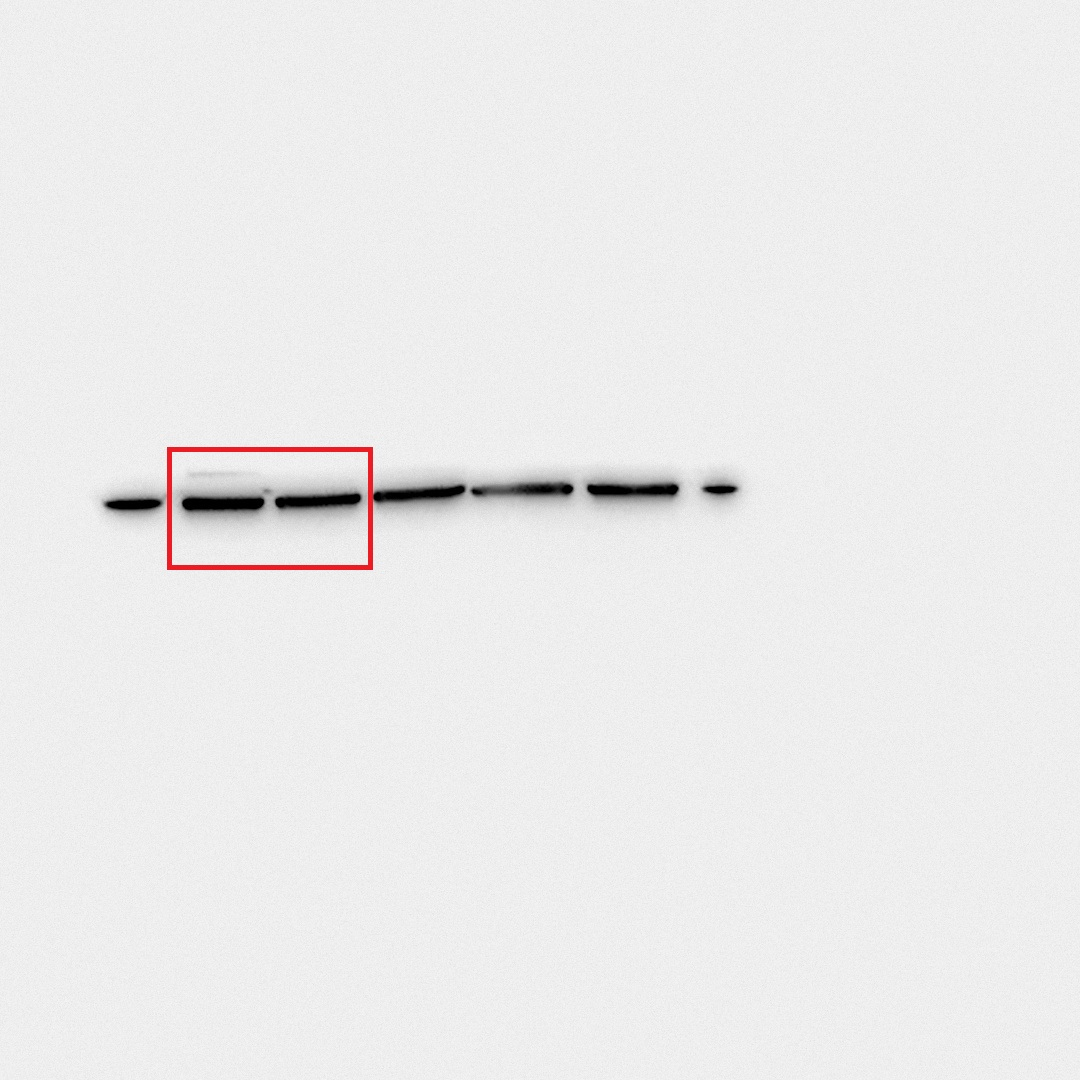

Supplement: Supplementary file 14 — Movie EV3 [file 44321_2025_289_MOESM14_ESM.zip › EMM-2025-21514_SourceData_Figure 4/4A/Actin 1.tiff]

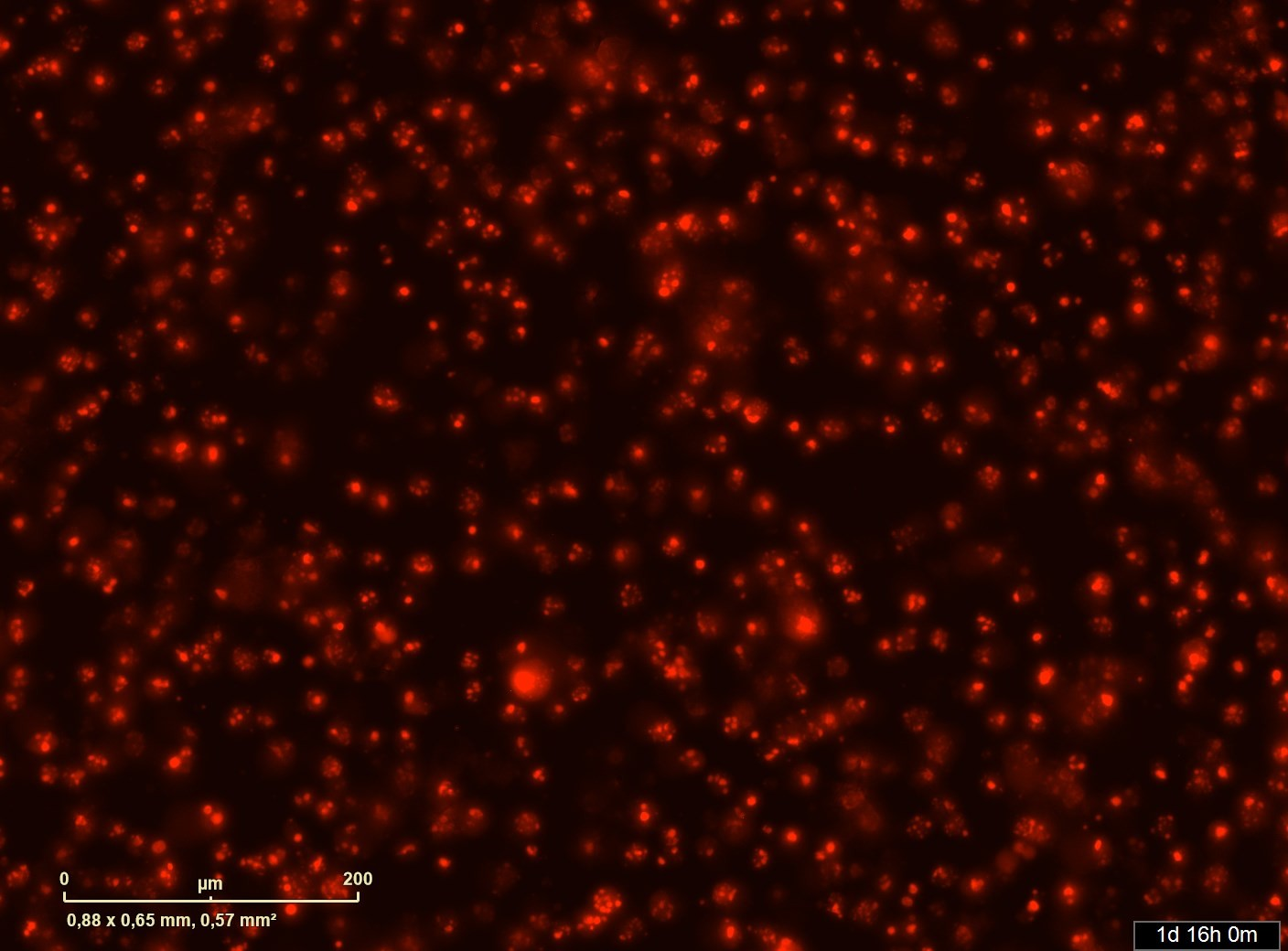

Supplement: Supplementary file 14 — Movie EV3 [file 44321_2025_289_MOESM14_ESM.zip › EMM-2025-21514_SourceData_Figure 4/4F/PI_sgIFT20.tiff]

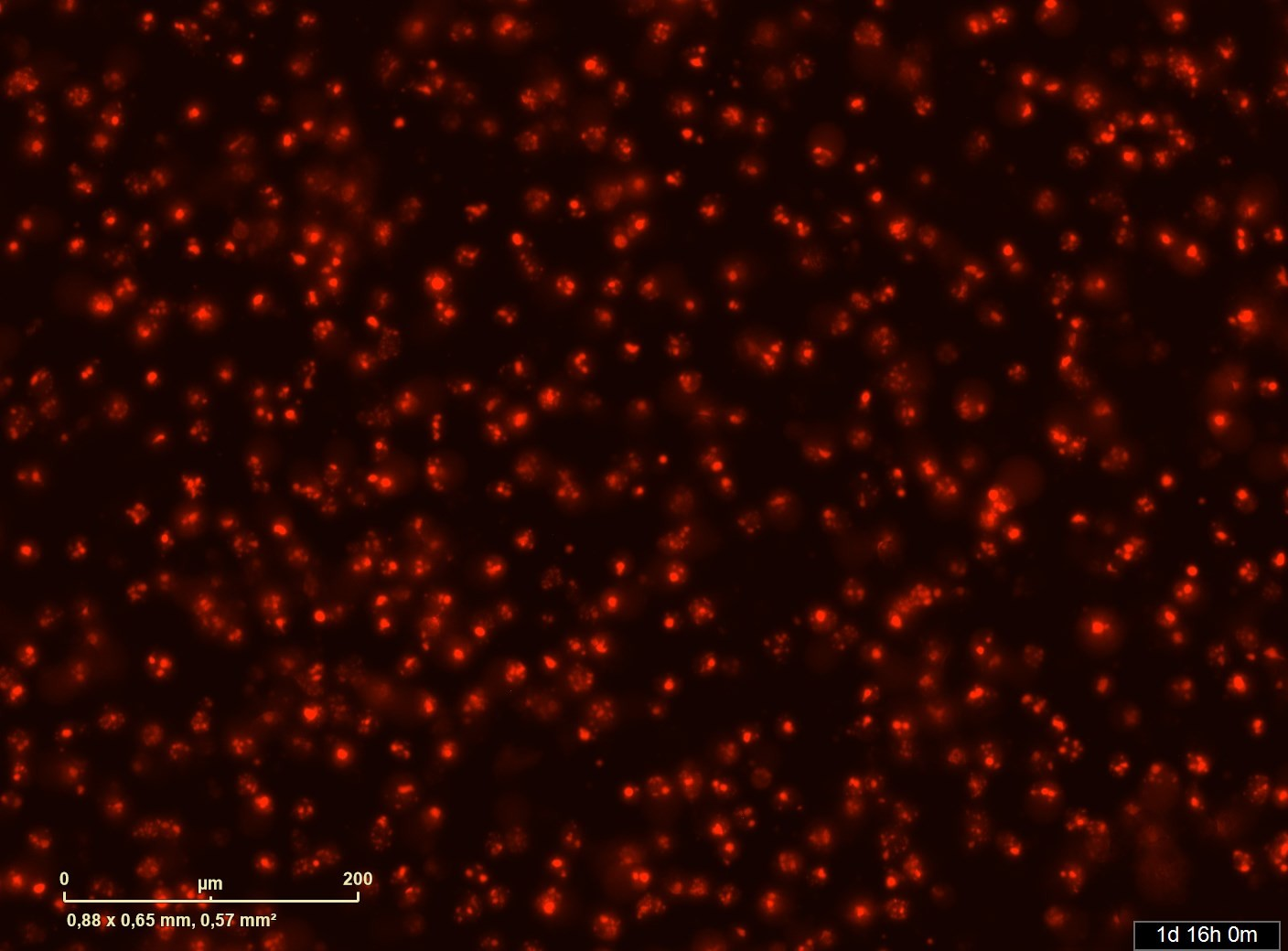

Supplement: Supplementary file 14 — Movie EV3 [file 44321_2025_289_MOESM14_ESM.zip › EMM-2025-21514_SourceData_Figure 4/4F/PI_sgKif3a.tiff]

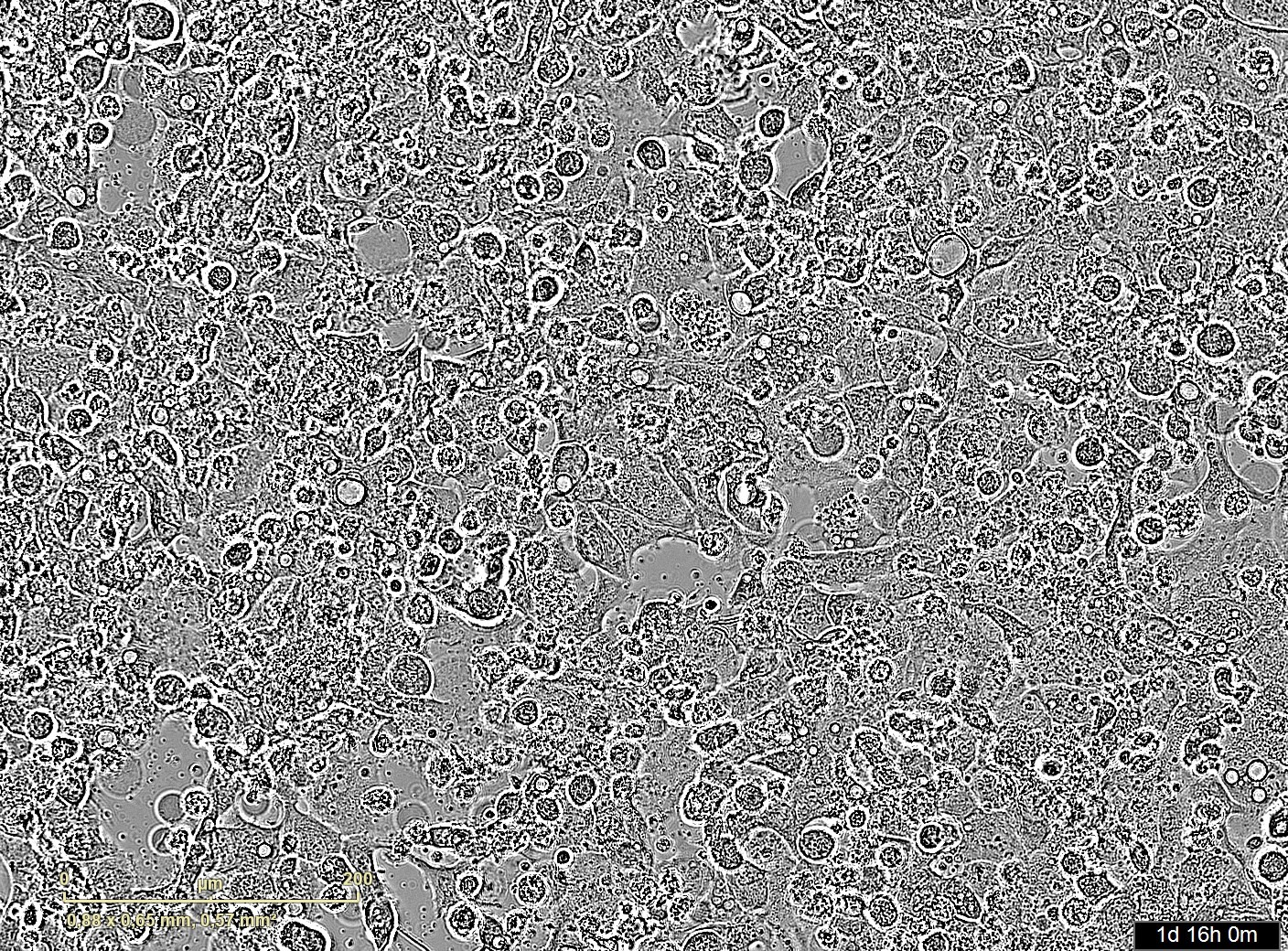

Supplement: Supplementary file 14 — Movie EV3 [file 44321_2025_289_MOESM14_ESM.zip › EMM-2025-21514_SourceData_Figure 4/4F/Phase_sgCTL.tiff]

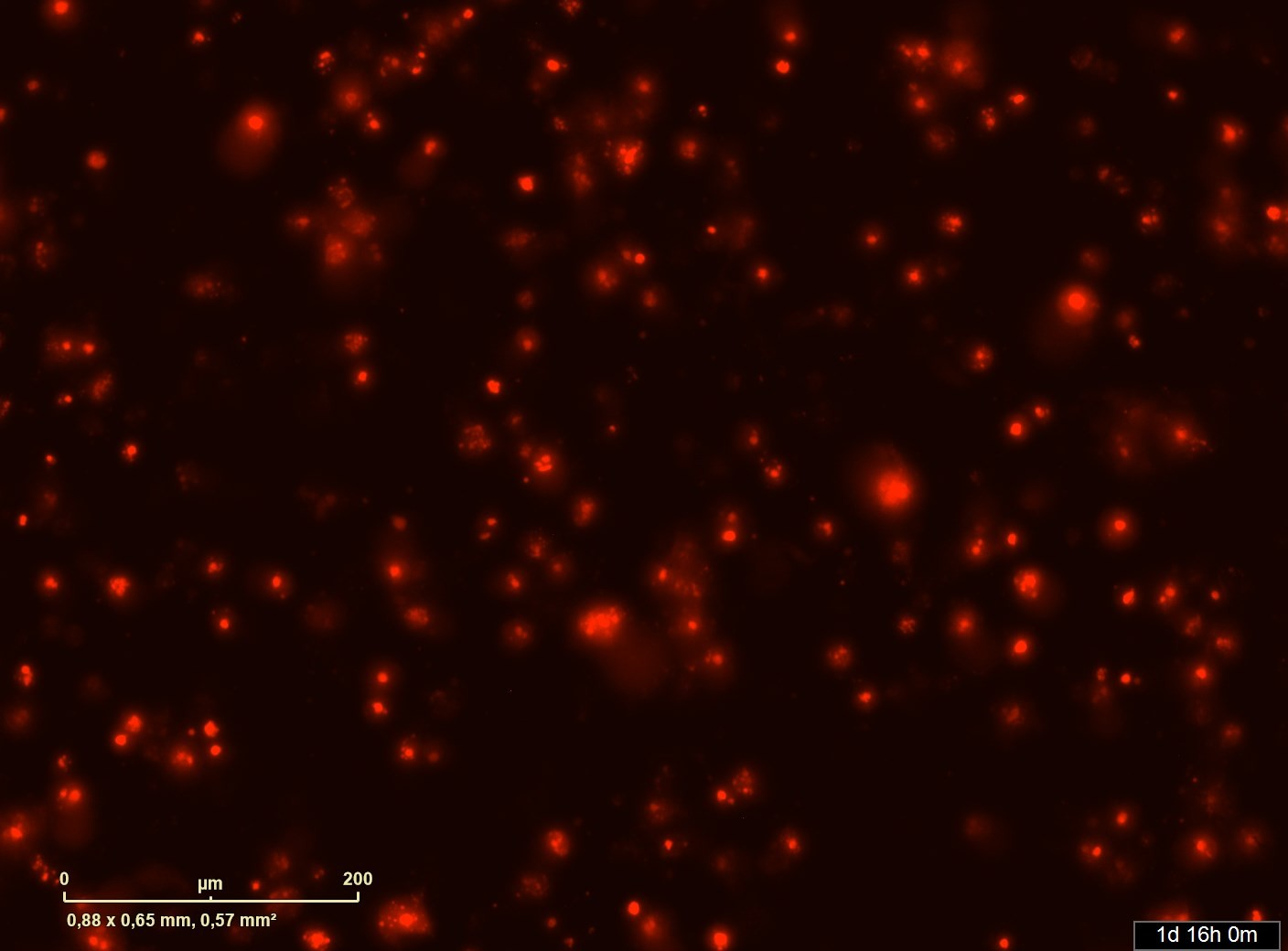

Supplement: Supplementary file 14 — Movie EV3 [file 44321_2025_289_MOESM14_ESM.zip › EMM-2025-21514_SourceData_Figure 4/4F/PI_sgCTL.tiff]

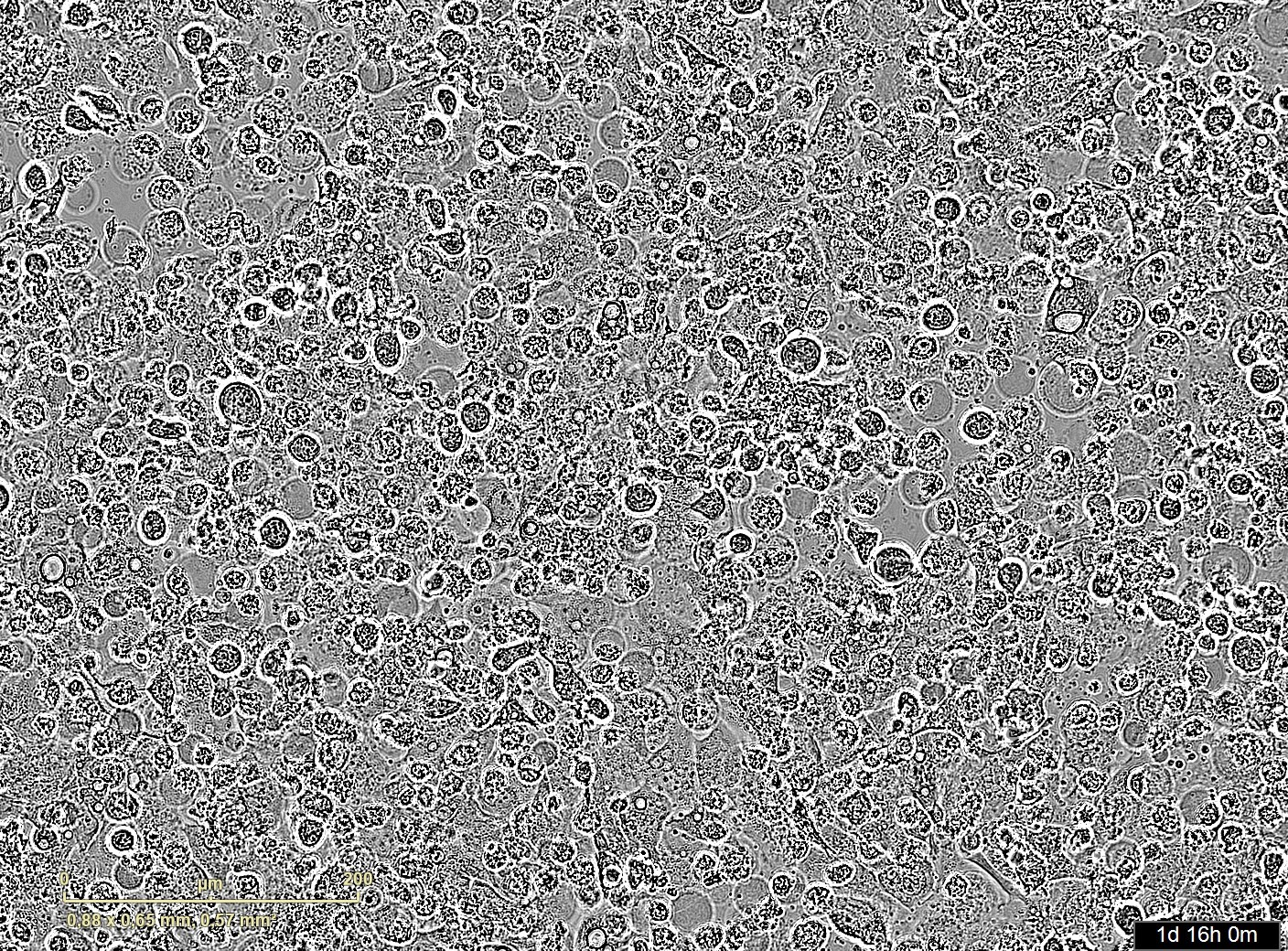

Supplement: Supplementary file 14 — Movie EV3 [file 44321_2025_289_MOESM14_ESM.zip › EMM-2025-21514_SourceData_Figure 4/4F/Phase_sgIFT20.tiff]

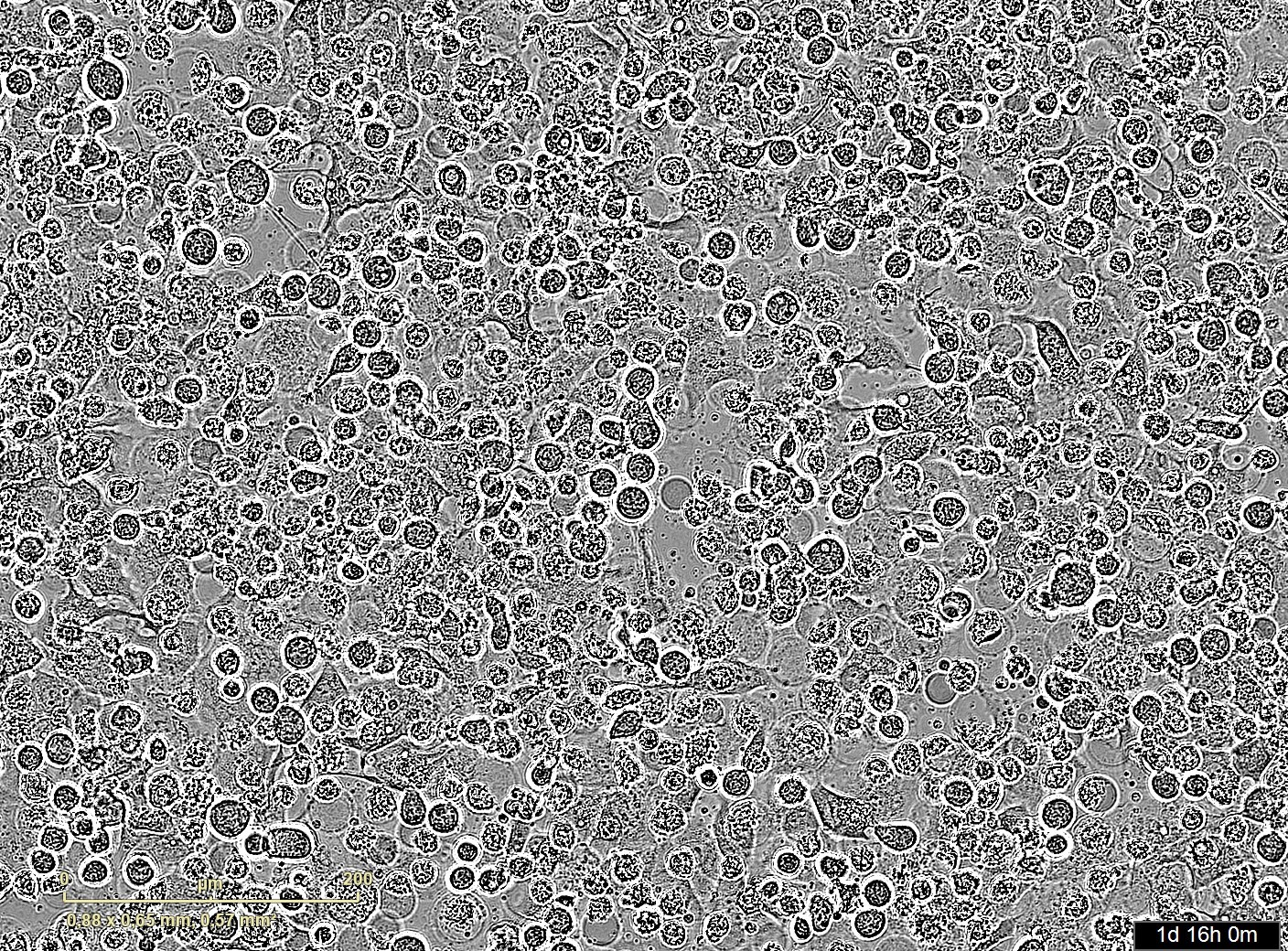

Supplement: Supplementary file 14 — Movie EV3 [file 44321_2025_289_MOESM14_ESM.zip › EMM-2025-21514_SourceData_Figure 4/4F/Phase_sgKif3a.tiff]

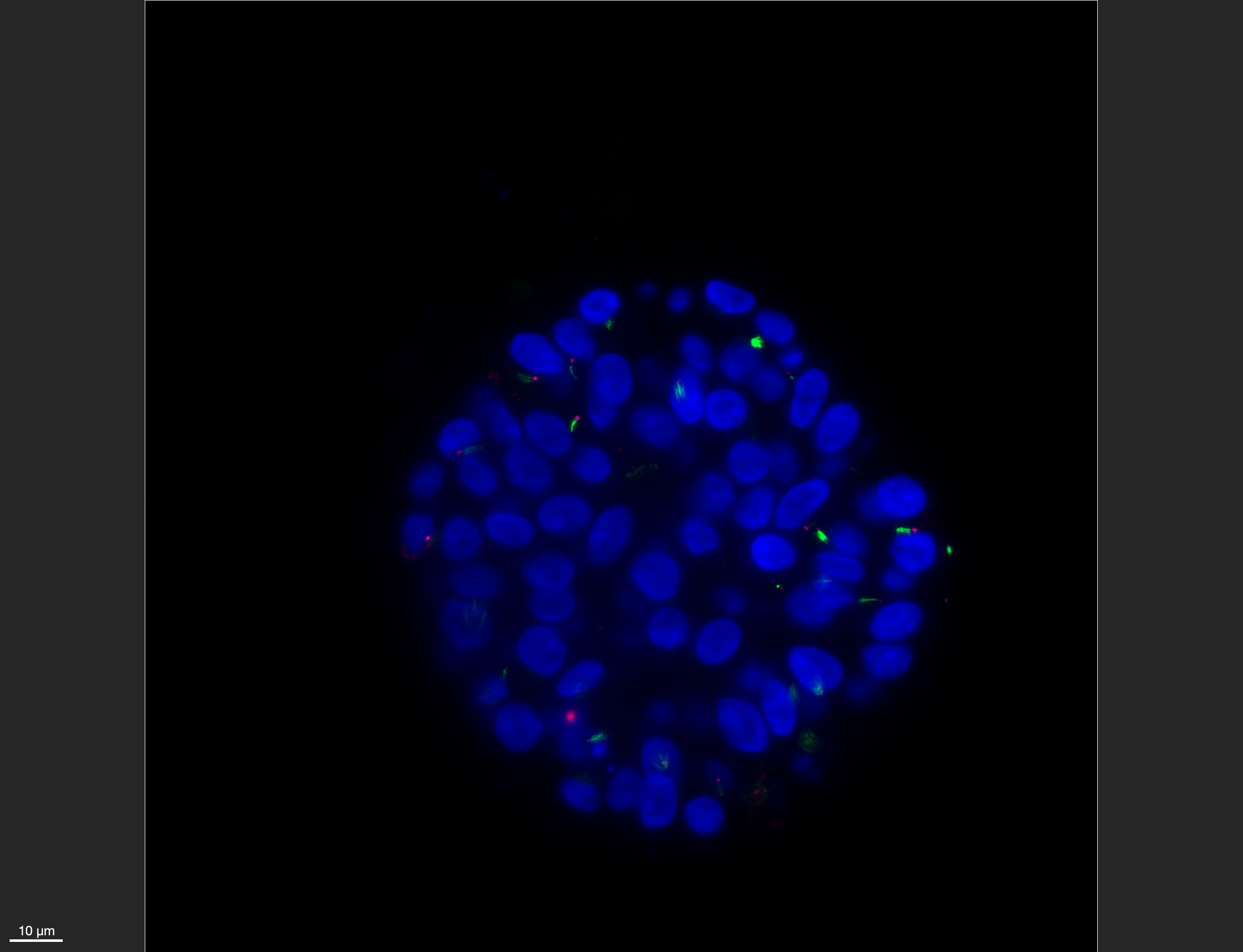

Supplement: Supplementary file 15 — Source data Fig. 1 [file 44321_2025_289_MOESM15_ESM.zip › EMM-2025-21514_SourceData_Figure 5/5A/PDOsPat.8_CTL.tif]

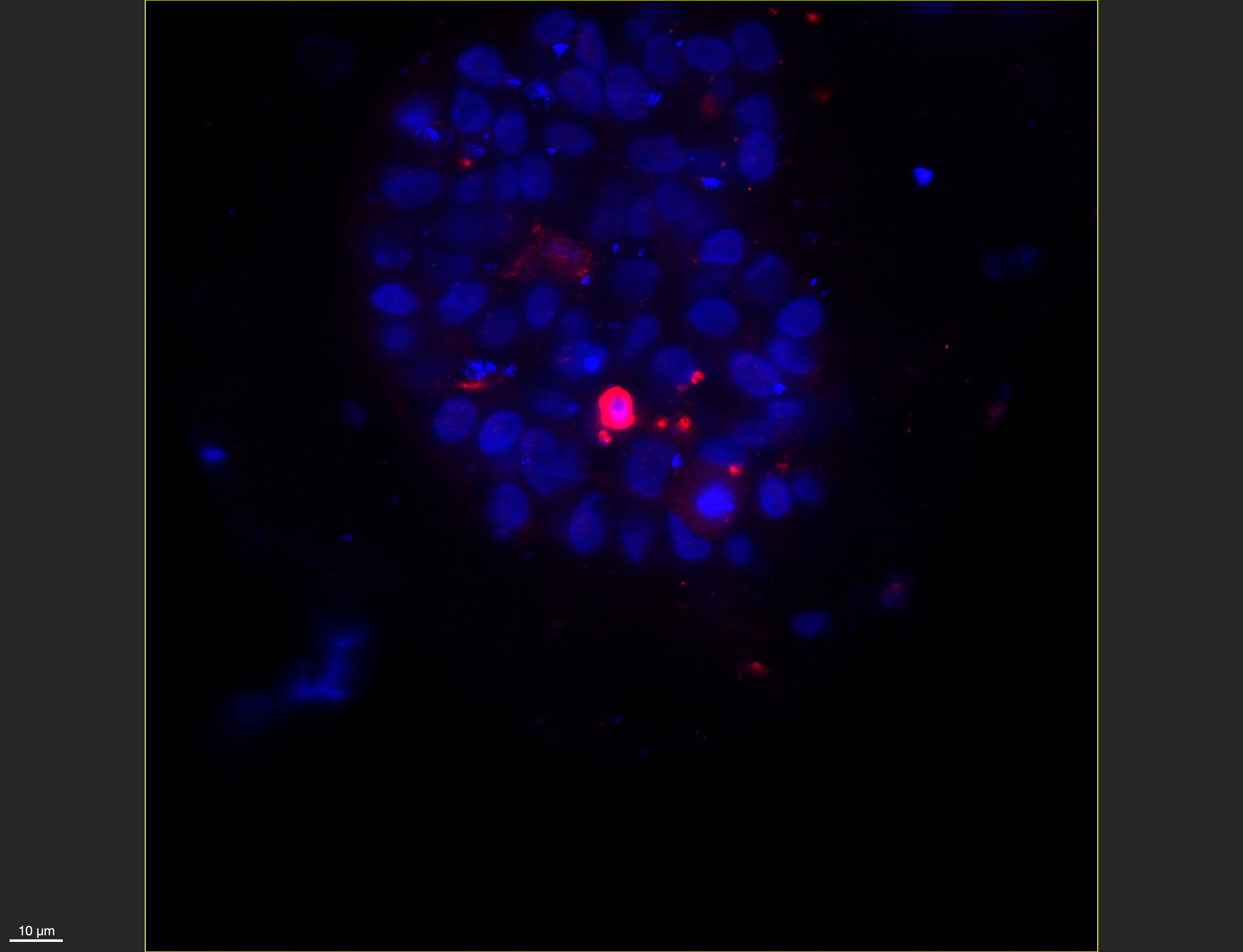

Supplement: Supplementary file 15 — Source data Fig. 1 [file 44321_2025_289_MOESM15_ESM.zip › EMM-2025-21514_SourceData_Figure 5/5A/PDOsPat.8_Nao-3.tif]

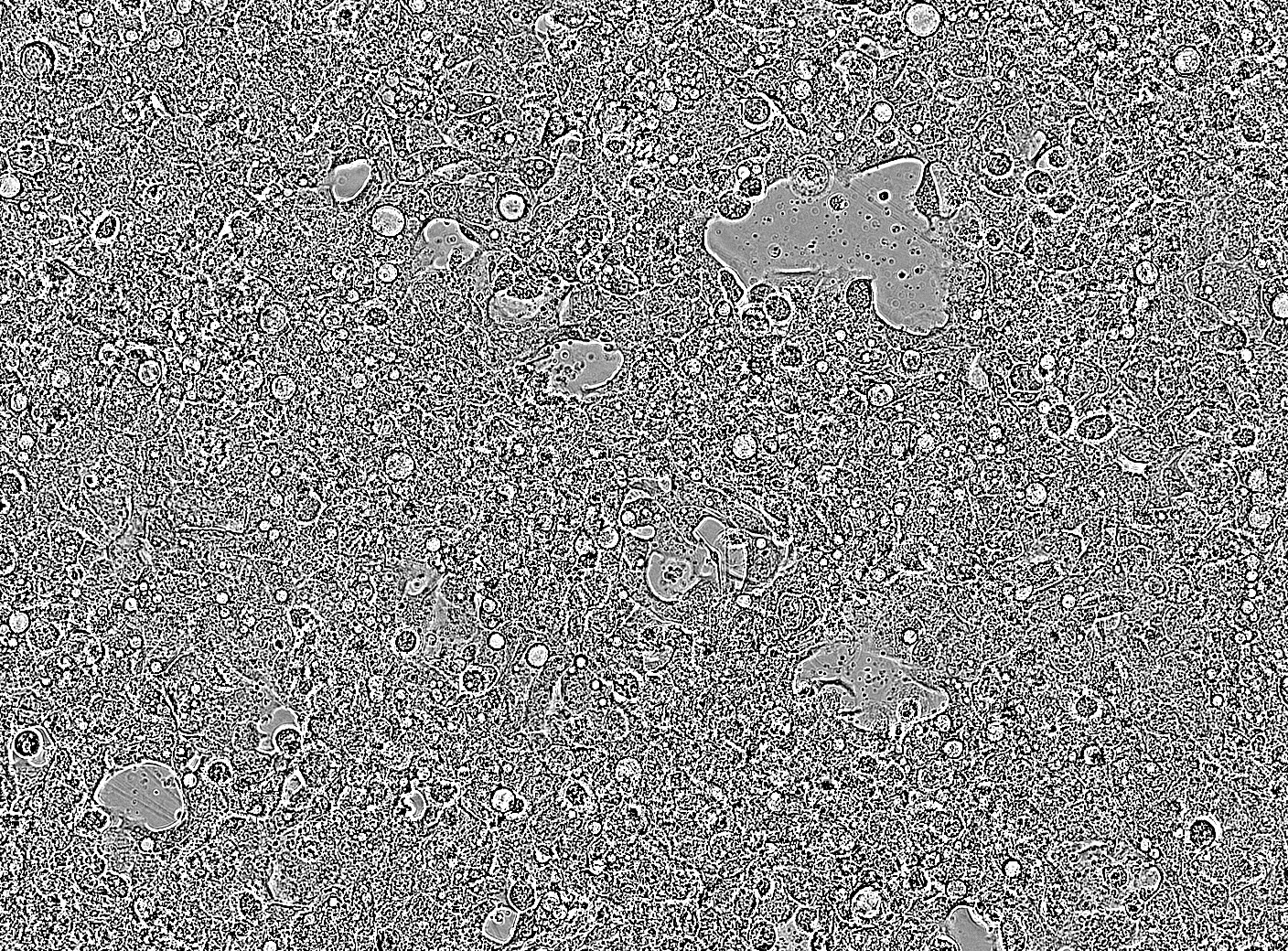

Supplement: Supplementary file 15 — Source data Fig. 1 [file 44321_2025_289_MOESM15_ESM.zip › EMM-2025-21514_SourceData_Figure 5/5K/Phase_Tax_sgCTL.tiff]

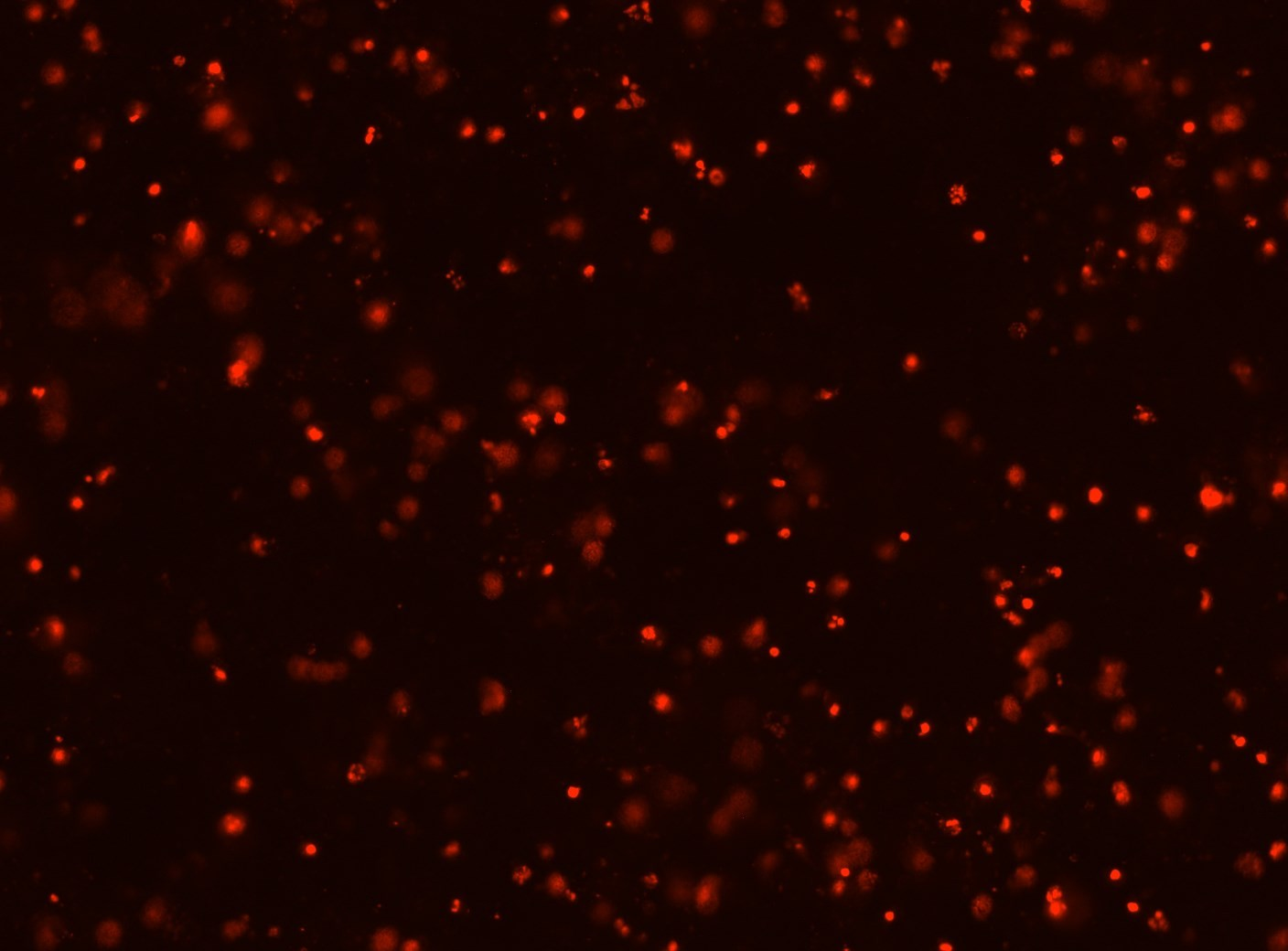

Supplement: Supplementary file 15 — Source data Fig. 1 [file 44321_2025_289_MOESM15_ESM.zip › EMM-2025-21514_SourceData_Figure 5/5K/PI_Tax_sgCTL.tiff]

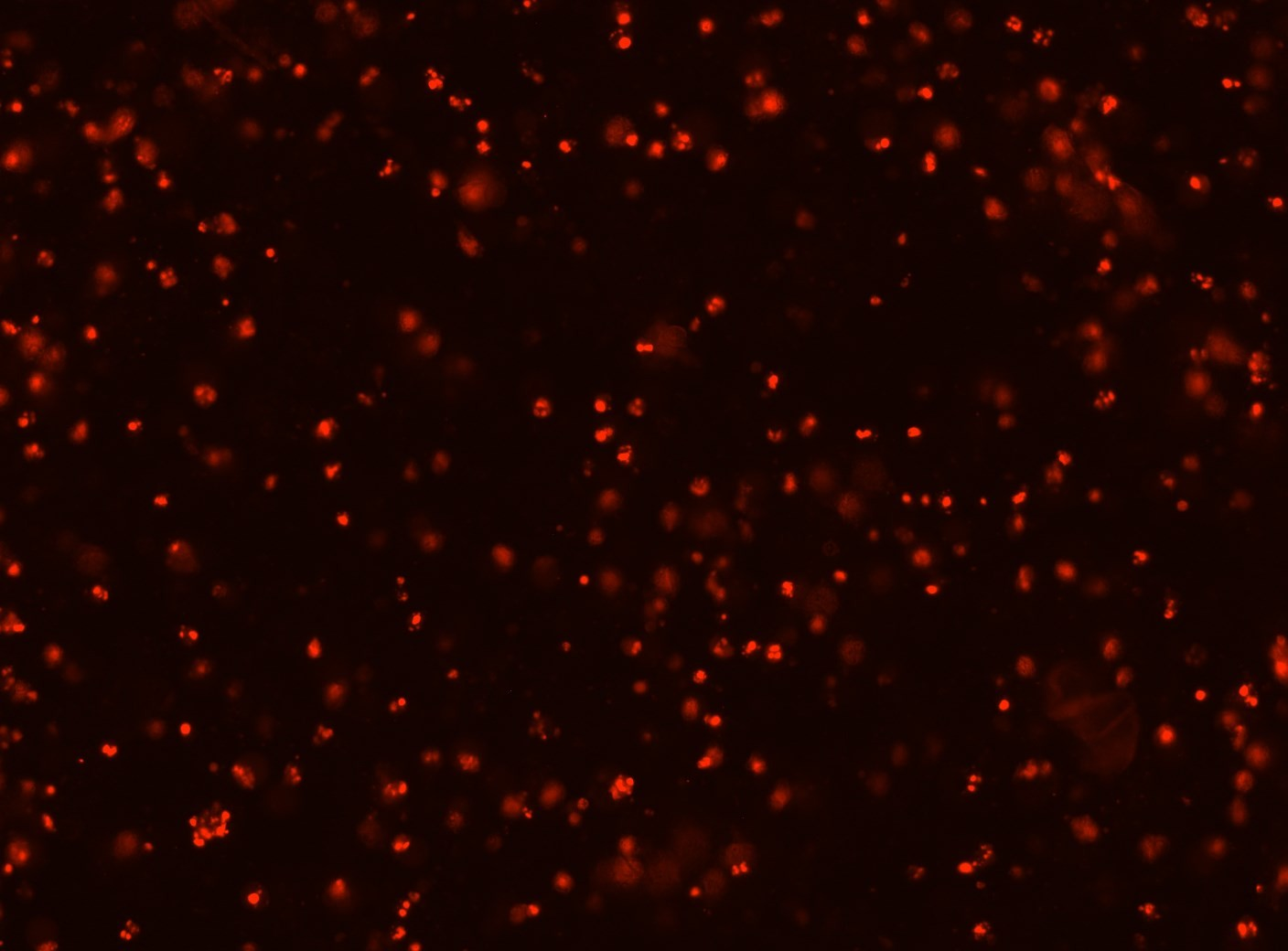

Supplement: Supplementary file 15 — Source data Fig. 1 [file 44321_2025_289_MOESM15_ESM.zip › EMM-2025-21514_SourceData_Figure 5/5K/PI_Tax_Bay_sgIFT20.tiff]

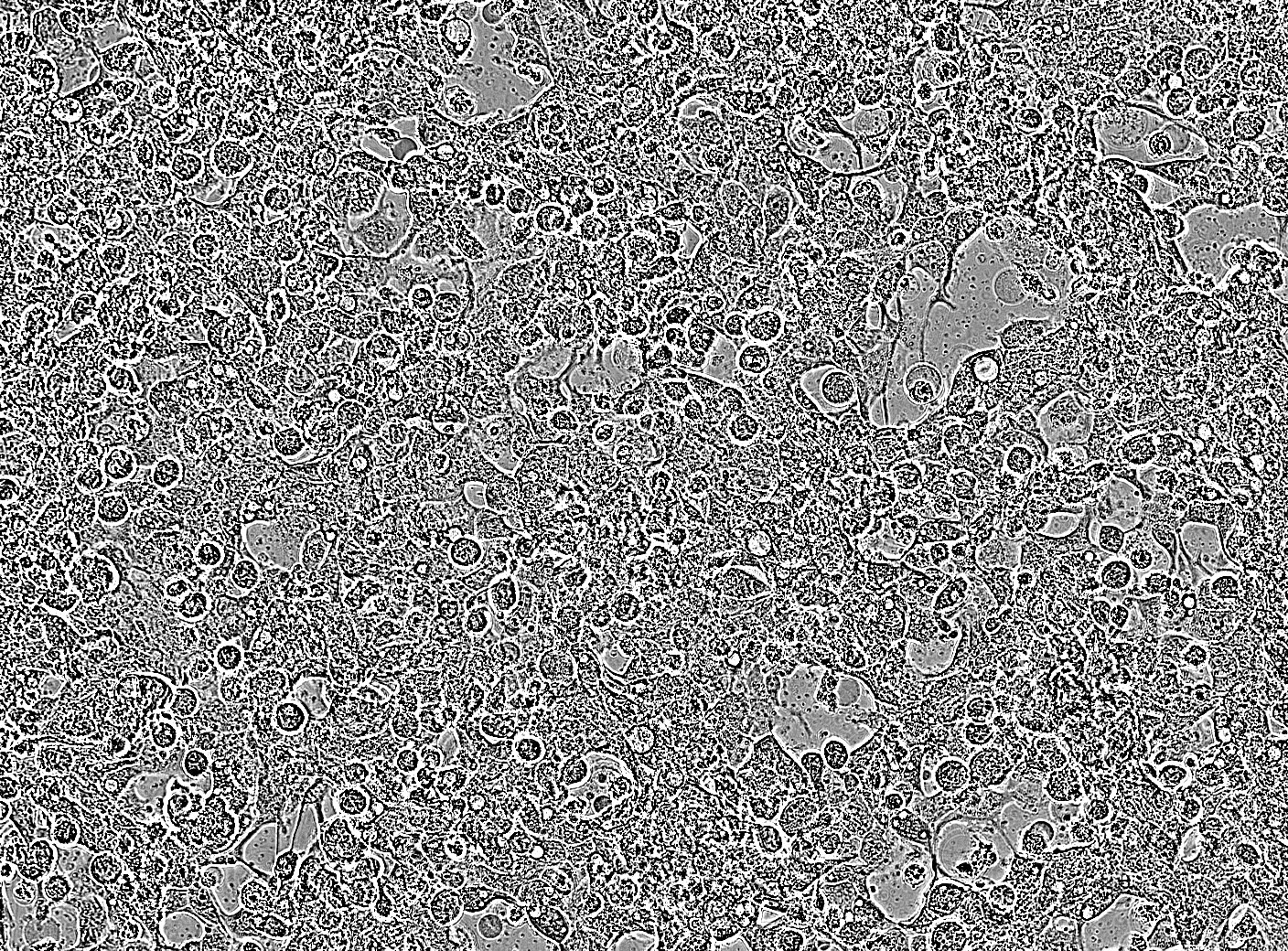

Supplement: Supplementary file 15 — Source data Fig. 1 [file 44321_2025_289_MOESM15_ESM.zip › EMM-2025-21514_SourceData_Figure 5/5K/Phase_Tax_Bay_sgIFT20.tiff]

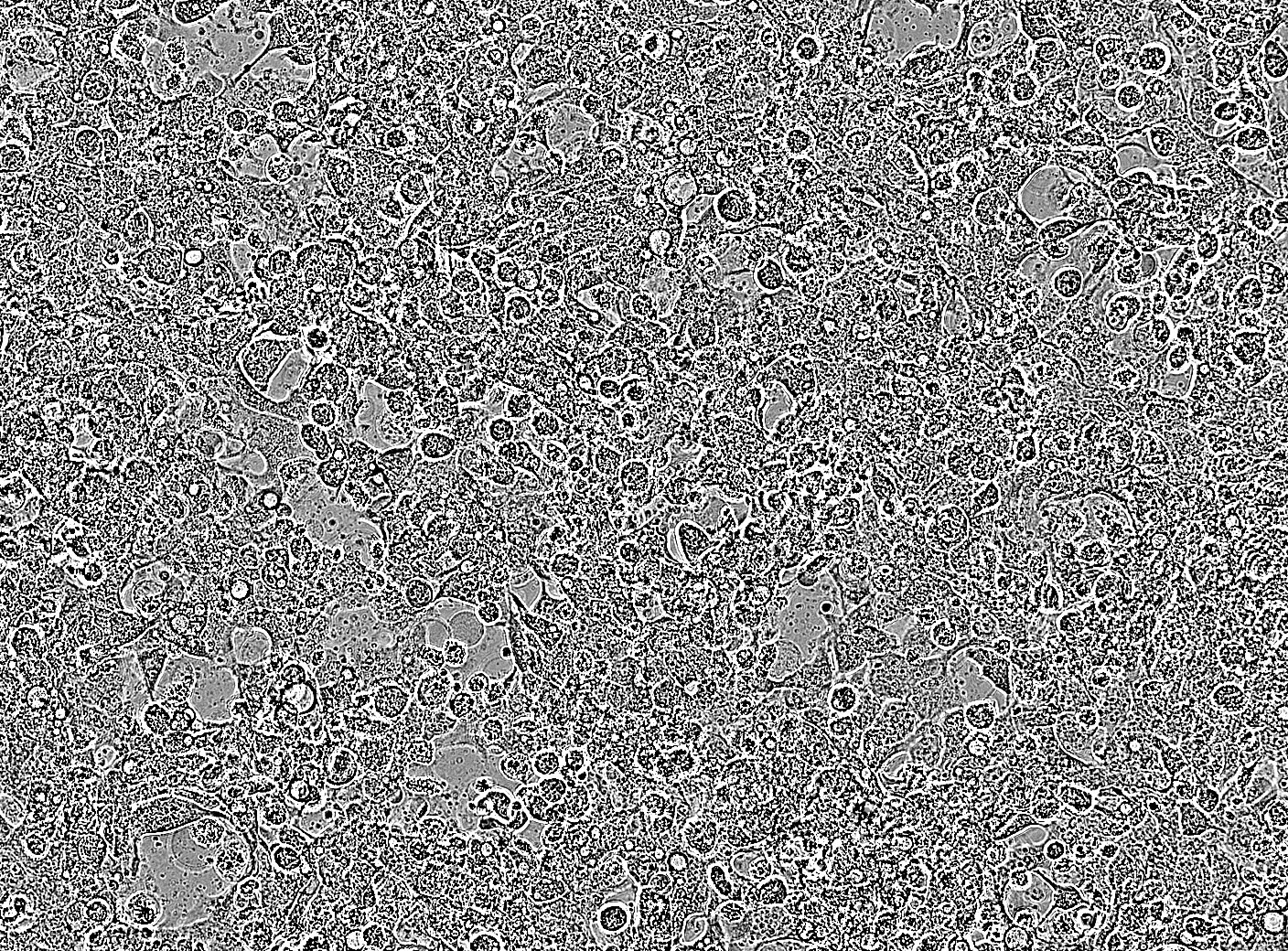

Supplement: Supplementary file 15 — Source data Fig. 1 [file 44321_2025_289_MOESM15_ESM.zip › EMM-2025-21514_SourceData_Figure 5/5K/Phase_Tax_sgIFT20.tiff]

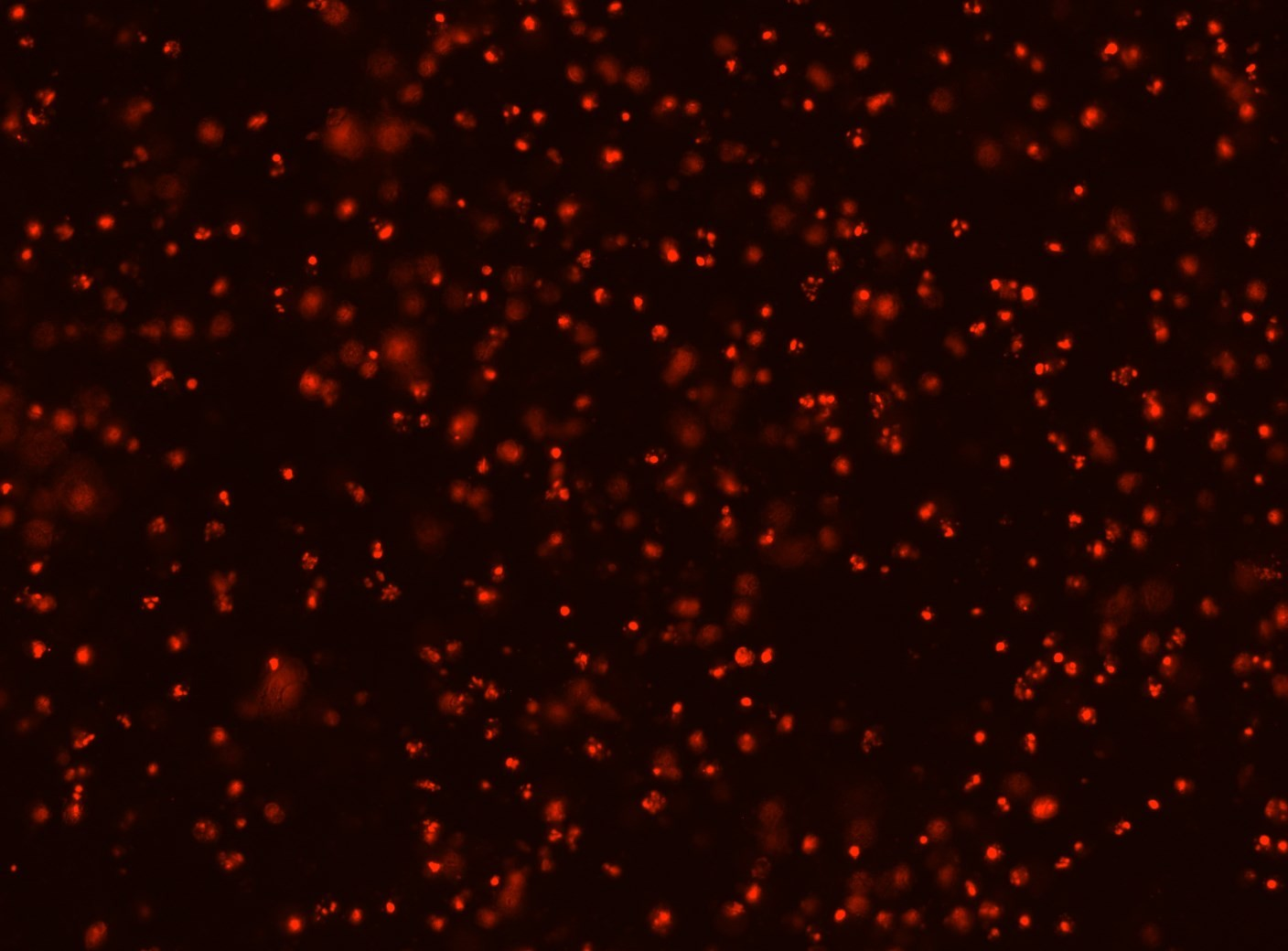

Supplement: Supplementary file 15 — Source data Fig. 1 [file 44321_2025_289_MOESM15_ESM.zip › EMM-2025-21514_SourceData_Figure 5/5K/PI_Tax_sgIFT20.tiff]
