# Supplementary material for: EMT-ciliary signaling in quasi-mesenchymal-stem-like cells drives therapeutic resistance and is a druggable vulnerability in triple-negative breast cancer
Source: EMBO Mol Med. 2025 Aug 26;17(10):2536–61. doi: 10.1038/s44321-025-00289-1 (PMC12514032; doi:10.1038/s44321-025-00289-1)
Supplement: Supplementary file 20 — Expanded View Figures [file 44321_2025_289_MOESM20_ESM.pdf]

Expanded View Figures

Figure EV1. Cancerous lesions in patient-derived biopsies and additional features of patient-derived cancer organoids.

(**A**) Paraffin sections from patient-derived biopsies (PDBs) were stained with H&E ($n = 13$, a representative result for patient#13 is shown), Scale bars: 2 mm (low magnification) and 100 µm (high magnification). (**B**) The morphology of PDOs from distinct patient samples (pat. #1-7) was examined by brightfield microscopy. Scale bar: 100 µm. (**C**) Paraffin sections of tumors were stained for the indicated proteins (a representative image is shown). Scale bar: 15 µm, inset: 3x. (**D**) Patient-Derived Organoids (PDOs) were stained for the indicated proteins (a representative image is shown). Scale bar: 15 µm, inset: 3x. (**E**) Heatmap showing inferred CNVs in cells of PDOs from patient sample#3 with unsupervised hierarchical clustering. (**F–I**) UMAP and violin plots illustrating the expression of mammary stem cell and pluripotency signatures in the distinct cell clusters composing PDOs from patient sample#3. Kruskal–Wallis test: ****$P < 2.2e\text{-}16$. Box plots show the median (center line), the 25th and 75th percentiles (lower and upper bounds of the box), and whiskers extending up to 1.5 times the interquartile range from the box limits. Data points beyond this range are considered outliers and are shown individually. Cluster 0 $n = 2201$, cluster 1 n = 1558, cluster 2, $n = 747$, cluster 3 $n = 242$, cluster 4 $n = 140$.

 © The Author(s)

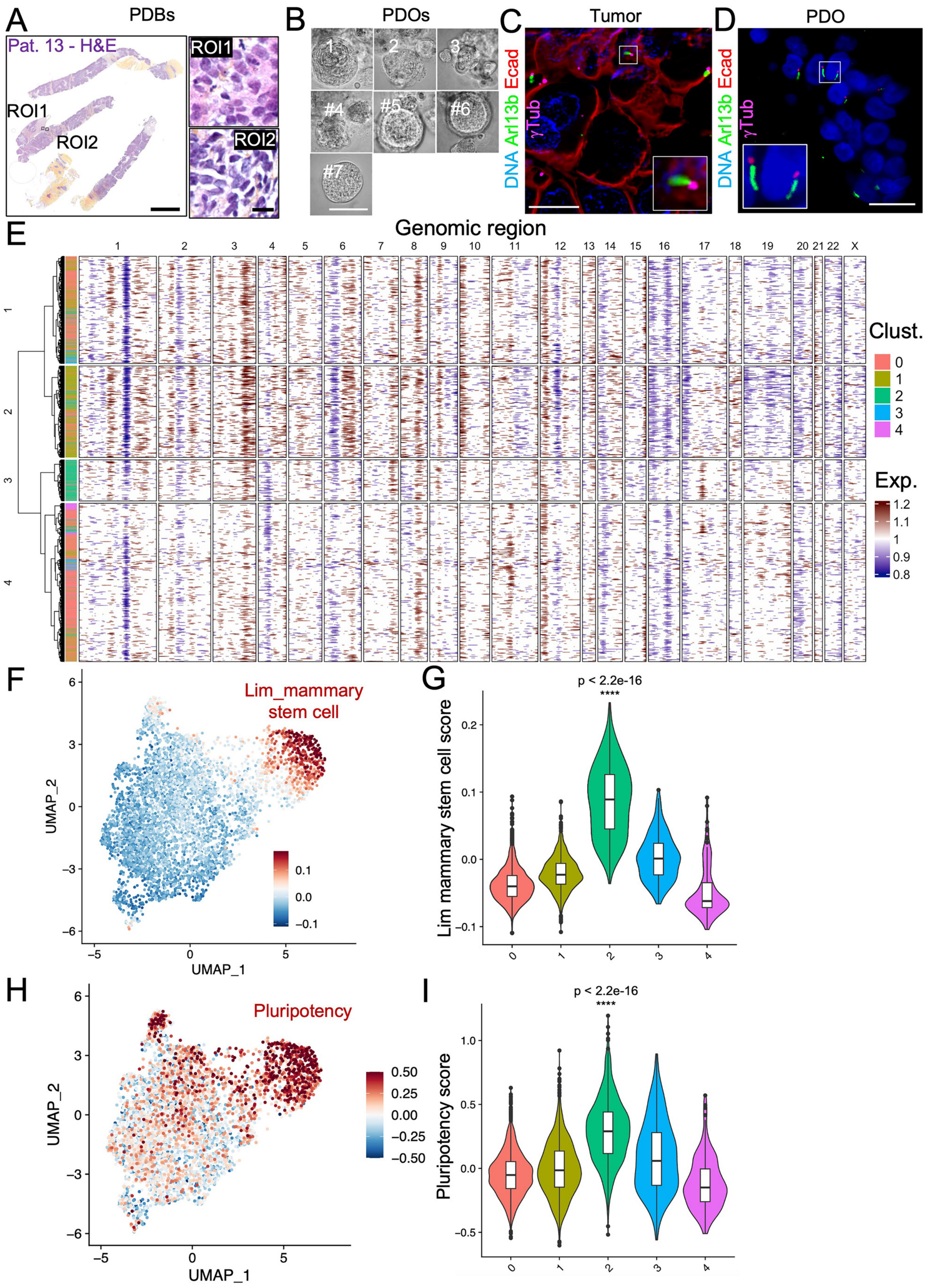
A
PDBs
Pat. 13 - H&E
ROI1
ROI2
B
PDOs
1
2
3
#4
#5
#6
#7
C
Tumor
DNA Arl13b Ecad γTub
D
PDO
DNA Arl13b Ecad γTub
E
Genomic region
1 2 3 4 5 6 7 8 9 10 11 12 13 14 15 16 17 18 19 20 21 22 X
Clust.
0
1
2
3
4
Exp.
1.2
1.1
1
0.9
0.8
F
Lim_mammary stem cell
UMAP_2
UMAP_1
0.1
0.0
−0.1
G
p < 2.2e-16
****
Lim mammary stem cell score
H
Pluripotency
UMAP_2
UMAP_1
0.50
0.25
0.00
−0.25
−0.50
I
p < 2.2e-16
****
Pluripotency score

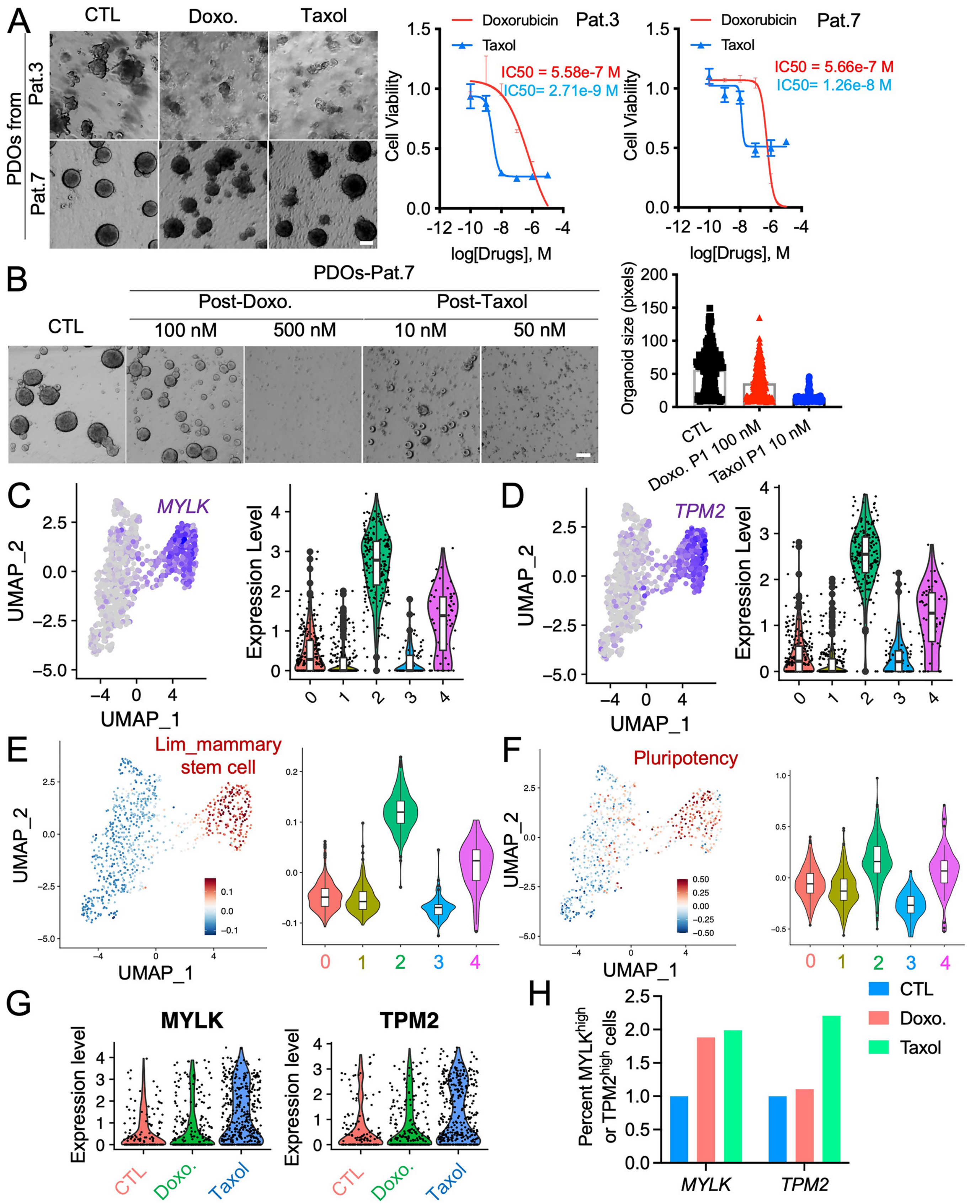
A
CTL
Doxo.
Taxol
PDOs from
Pat.3
Pat.7
Doxorubicin
Taxol
Pat.3
IC50 = 5.58e-7 M
IC50= 2.71e-9 M
Cell Viability
log[Drugs], M
Pat.7
IC50 = 5.66e-7 M
IC50= 1.26e-8 M
B
PDOs-Pat.7
Post-Doxo.
Post-Taxol
CTL
100 nM
500 nM
10 nM
50 nM
Organoid size (pixels)
CTL
Doxo. P1 100 nM
Taxol P1 10 nM
C
MYLK
UMAP_2
UMAP_1
Expression Level
D
TPM2
E
Lim_mammary stem cell
F
Pluripotency
G
MYLK
TPM2
Expression level
CTL
Doxo.
Taxol
H
Percent MYLKhigh or TPM2high cells
CTL
Doxo.
Taxol
MYLK
TPM2

Figure EV2. Impact of chemotherapy on PDOs and additional features of quasi-mesenchymal ciliated stem-like cells.

(**A**) The dose-dependent impact of chemotherapy on cell viability in PDOs was tested for two distinct patient (pat.) samples (#3 and #7). Representative images of PDOs treated with DMSO (CTL) or with doxorubicin (doxo., 100 nM) and Taxol (10 nM) after 48 h of treatment are shown. The cell viability was measured at different concentrations and the IC50 is shown for each drug (mean ± s.e.m., representative analysis of 3 independent experiments). Scale bar: 100 µm. (**B**) The impact of drugs on the ability of chemoresistant cancer cells to reconstitute PDOs after drug removal at two intermediate drug concentrations is shown and measured at the indicated concentrations. Scale bar: 100 µm (n ≥213 PDOs/treatment condition, mean ± s.e.m.). (**C**–**F**) UMAP and violin plots illustrating the expression of *MYLK*, *TPM2*, mammary stem cell and pluripotency signatures in the distinct cell clusters from PDOs. Box plots show the median (center line), the 25th and 75th percentiles (lower and upper bounds of the box), and whiskers extending up to 1.5 times the interquartile range from the box limits. Data points beyond this range are considered outliers and are shown individually. Cluster 0 $n = 251$, cluster 1 $n = 234$, cluster 2 $n = 213$, cluster 3 $n = 66$, cluster 4 $n = 56$. (**G**, **H**) Expression levels of *MYLK* and *TPM2* was examined in cells from control and post-chemotherapy PDOs. The proportion of cells expressing high levels of *MYLK* and *TPM2* (> 2) was determined for each sample and normalized to the control. *MYLK* > 2 CTL $n = 12$, Doxo. $n = 42$, Taxol $n = 131$; *TPM2* > 2 CTL $n = 18$, Doxo. $n = 37$, Taxol $n = 128$.

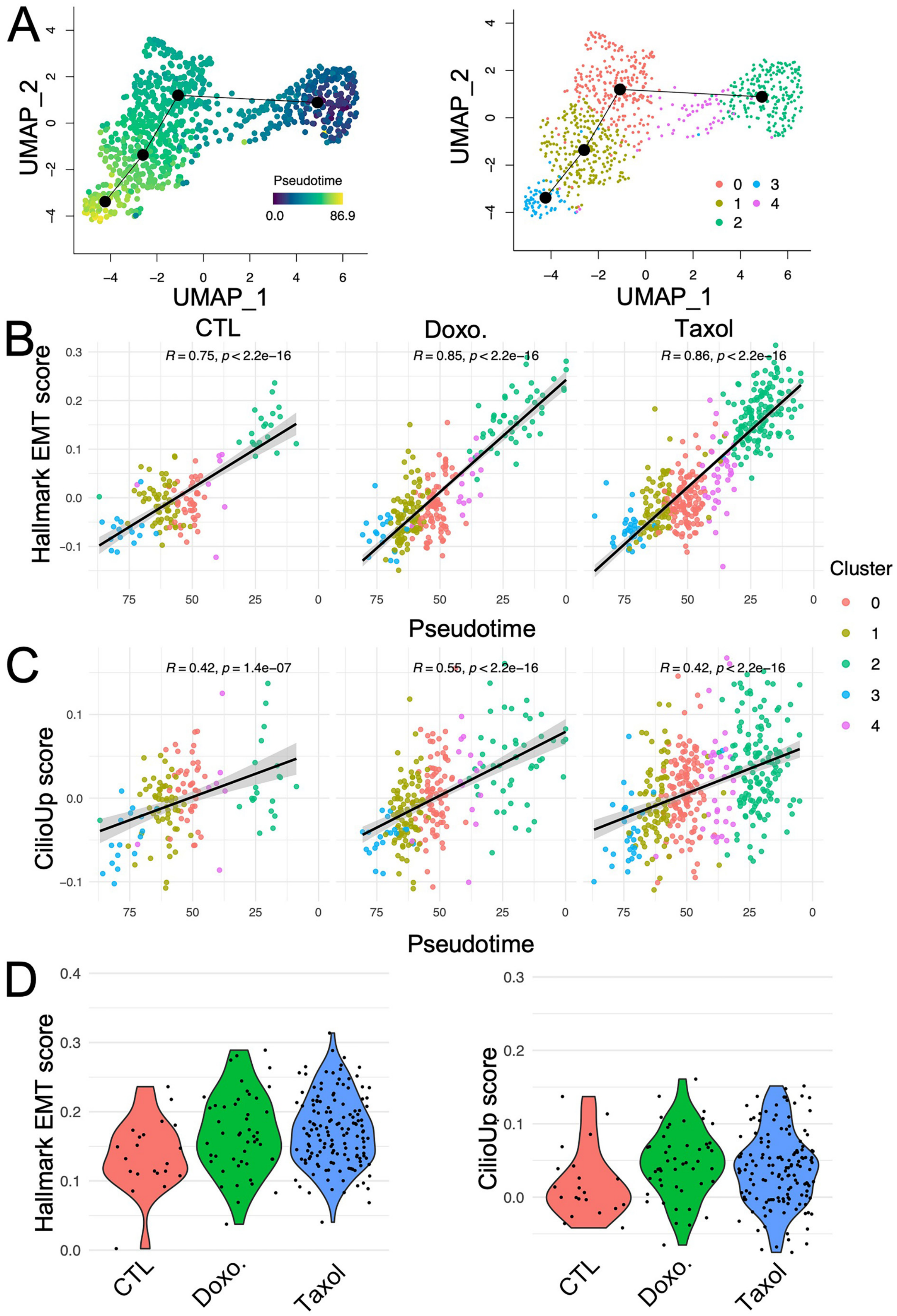
A
UMAP_2
UMAP_1
Pseudotime
0.0
86.9
0
1
2
3
4
B
CTL
Doxo.
Taxol
R = 0.75, p < 2.2e−16
R = 0.85, p < 2.2e−16
R = 0.86, p < 2.2e−16
Hallmark EMT score
Pseudotime
Cluster
C
R = 0.42, p = 1.4e−07
R = 0.55, p < 2.2e−16
R = 0.42, p < 2.2e−16
CilioUp score
Pseudotime
D
Hallmark EMT score
CilioUp score
CTL
Doxo.
Taxol

© The Author(s)

Figure EV3. Distribution of cells along the pseudotime trajectory and progressive expression of EMT and ciliogenesis programs.

(**A**) UMAP combining data from 3 samples (PDOs from patient sample #3, CTL/Doxo./Taxol) colored by pseudotime value. Cells are ordered along an inferred trajectory; a black line connects all clusters. (**B**, **C**) Distribution of cells from distinct samples and clusters along the pseudotime trajectory and correlative analysis of the expression of EMT and CilioUp signatures. Wilcoxon test: Hallmark EMT score CTL $R = 0.75$ $P < 2.2e\text{-}16$, Doxo. $R = 0.85$ $P < 2.2e\text{-}16$, Taxol $R = 0.86$ $P < 2.2e\text{-}16$; CilioUp score CTL $R = 0.42$ $P = 1.4e\text{-}07$, Doxo. $R = 0.56$ $P < 2.2e\text{-}16$, Taxol $R = 0.42$ $P < 2.2e\text{-}16$. (**D**) Violin plots illustrating the expression of EMT and ciliogenesis transcriptional programs in individual cells of cluster 2 per treatment condition. CTL $n = 21$, Doxo. $n = 48$, Taxol $n = 144$.

© The Author(s)

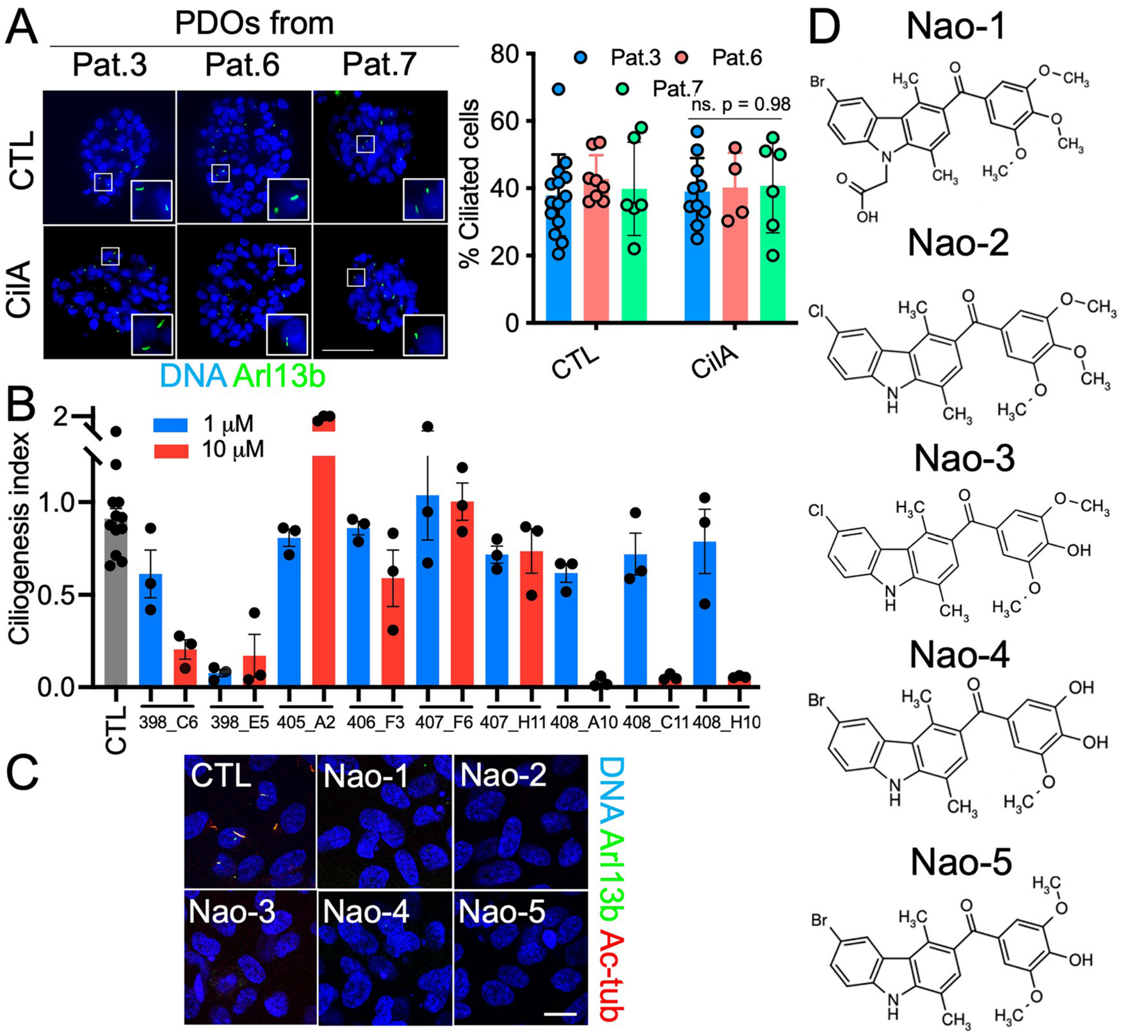

Figure EV4. Identification of small-molecule inhibitors of primary ciliogenesis.

(**A**) PDOs from distinct patient (pat.) samples were treated with DMSO (CTL) or Ciliobrevin A (CilA, 100 µM, 24 h) and stained for Arl13b. Scale bar: 50 µm. The percentage of ciliated cells was quantified in distinct PDOs for each patient sample ($n \geq 4$ PDOs/patient sample/treatment condition, means ± s.e.m.; Student's t test: CTL vs. CilA n.s. $P = 0.98$). (**B**) A secondary screen with small molecules identified as candidate ciliogenesis inhibitors in a primary screen (Fig. 3A) at two distinct concentrations (1 and 10 µM) was conducted. The ciliogenesis index was determined 24 h after each treatment. Results are relative to CTL, DMSO-treated cells ($n = 3$, mean ± s.e.m.). (**C**) The impact of the most potent inhibitors on ciliogenesis is shown (10 µM, Nao-1 = 398_C6; Nao-2 = 398-E5; Nao-3 = 408-A10; Nao-4 = 408_C11; Nao-5 = 408_H10). Scale bar: 15 µm. (**D**) Chemical structures of Naonedins.

 © The Author(s)

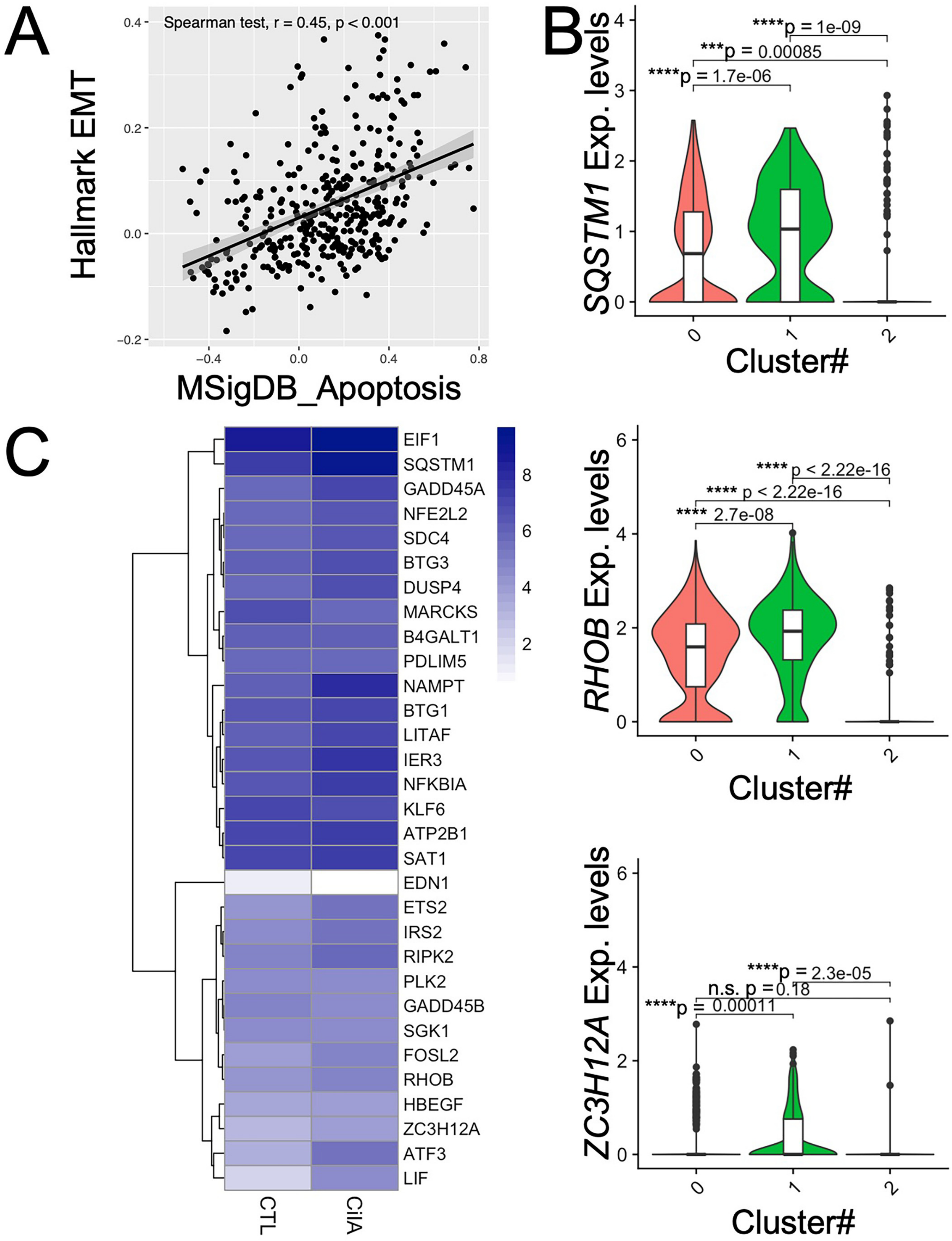
A
Spearman test, r = 0.45, p < 0.001
Hallmark EMT
MSigDB_Apoptosis
B
SQSTM1 Exp. levels
****p = 1e-09
***p = 0.00085
****p = 1.7e-06
Cluster#
RHOB Exp. levels
**** p < 2.22e-16
**** p < 2.22e-16
**** 2.7e-08
ZC3H12A Exp. levels
****p = 2.3e-05
n.s. p = 0.18
****p = 0.00011
C
EIF1
SQSTM1
GADD45A
NFE2L2
SDC4
BTG3
DUSP4
MARCKS
B4GALT1
PDLIM5
NAMPT
BTG1
LITAF
IER3
NFKBIA
KLF6
ATP2B1
SAT1
EDN1
ETS2
IRS2
RIPK2
PLK2
GADD45B
SGK1
FOSL2
RHOB
HBEGF
ZC3H12A
ATF3
LIF
CTL
CilA

Figure EV5. Mechanisms of EMT-cilium-dependent regulation of chemoresistance.

(**A**) The correlation in the expression levels of EMT and apoptosis gene expression signatures in cancer cells from PDOs treated with Nao-3 was determined using a Spearman test, $r = 0.45$, ***$P < 0.001$. (**B**) Violin plots illustrating the expression levels of the indicated gene transcripts in cell clusters from DMSO-treated and Nao-3-treated PDOs. Kruskal–Wallis test: ns. $P = 0.18$; ***$P = 0.00085$; ****$P < 0.0001$. Box plots show the median (center line), the 25th and 75th percentiles (lower and upper bounds of the box), and whiskers extending up to 1.5 times the interquartile range from the box limits. Data points beyond this range are considered outliers and are shown individually. Cluster 0 $n = 499$, cluster 1 $n = 281$, cluster 2 $n = 122$. (**C**) Heatmap illustrating the expression levels of the indicated gene transcripts in HMLER shEcad cells treated with a vehicle control (CTL, DMSO) or Ciliobrevin A (CilA). GSE160549 dataset.

 © The Author(s)
