# Supplementary material for: EMT-ciliary signaling in quasi-mesenchymal-stem-like cells drives therapeutic resistance and is a druggable vulnerability in triple-negative breast cancer
Source: EMBO Mol Med. 2025 Aug 26;17(10):2536–61. doi: 10.1038/s44321-025-00289-1 (PMC12514032; doi:10.1038/s44321-025-00289-1)
Supplement: Supplementary file 1 — Table EV1 [file 44321_2025_289_MOESM1_ESM.pdf]

EMBO Molecular Medicine

EMT-ciliary signaling in quasi-mesenchymal stem-like cells mediates TNBC therapeutic resistance

Camille Tessier, Jennifer Derrien, Aurore Dupuy, Thomas Pelé, Martin Moquet, Julie Roul, Elise Douillard, Camille El Harrif, Xavier Pinson, Matthieu Le Gallo, Florence Godey, Patrick Tas, Roselyne Viel, Eloise Grasset, Claude Prigent, Eric Letouzé, Peggy Suzanne, Patrick Dallemagne, Mario Campone, Robert A. Weinberg, Jacqueline Lees, Philippe Juin, and Vincent Guen

Corresponding author: Vincent Guen (vincent.guen@inserm.fr)

Review Timeline:

Submission Date:	28th Feb 25
Editorial Decision:	9th Apr 25
Revision Received:	10th Jun 25
Editorial Decision:	27th Jun 25
Revision Received:	2nd Jul 25
Accepted:	9th Jul 25

Editor: Lise Roth

Transaction Report:

(Note: With the exception of the correction of typographical or spelling errors that could be a source of ambiguity, letters and reports are not edited. Depending on transfer agreements, referee reports obtained elsewhere may or may not be included in this compilation. Referee reports are anonymous unless the Referee chooses to sign their reports.)

9th Apr 2025

Dear Dr. Guen,

Thank you for submitting your manuscript to EMBO Molecular Medicine and please accept my apologies for the delay in getting back to you as we were waiting for a referee report. Unfortunately, referee #3 has not yet got back to us, but given that both referees #1 and #2 make similar recommendations, we prefer to make a decision now to avoid further delay in the process. Should referee #3 provide a report, we will send it to you on the understanding that we will not ask you to carry out any extensive experiments beyond those requested in the attached reports from referees #1 and #2. As you will see from the reports below, the referees recognise the interest of the study and generally support publication of your work, subject to appropriate revisions.

Addressing the reviewers' concerns in full will be necessary for further considering the manuscript in our journal, and acceptance of the manuscript will entail a second round of review. EMBO Molecular Medicine encourages a single round of revision only and therefore, acceptance or rejection of the manuscript will depend on the completeness of your responses included in the next, final version of the manuscript. For this reason, and to save you frustration at the end, I would strongly discourage you from returning an incomplete revision.

If you would like to discuss further the points raised by the referees, I am available to do so via email or video. Let me know if you are interested in this option.

We are expecting your revised manuscript within three months, if you anticipate any delay, please contact us.

When submitting your revised manuscript, please carefully review the instructions that follow below. We perform an initial quality control of all revised manuscripts before re-review; failure to include requested items will delay the evaluation of your revision.

We require:

1) A .docx formatted version of the manuscript text (including legends for main figures, EV figures and tables). Please make sure that the changes are highlighted to be clearly visible.

2) Individual production quality figure files as .eps, .tif, .jpg (one file per figure). For guidance, download the 'Figure Guide PDF' (https://www.embopress.org/page/journal/17574684/authorguide#figureformat).

3) At EMBO Press we ask authors to provide source data for the main figures. Our source data coordinator will contact you to discuss which figure panels we would need source data for and will also provide you with helpful tips on how to upload and organize the files.

4) A .docx formatted letter INCLUDING the reviewers' reports and your detailed point-by-point responses to their comments. As part of the EMBO Press transparent editorial process, the point-by-point response is part of the Review Process File (RPF), which will be published alongside your paper.

5) A complete author checklist, which you can download from our author guidelines (https://www.embopress.org/page/journal/17574684/authorguide#submissionofrevisions). Please insert information in the checklist that is also reflected in the manuscript. The completed author checklist will also be part of the RPF.

6) All Materials and Methods need to be described in the main text using our 'Structured Methods' format. According to this format, the Methods section includes a Reagents and Tools Table (listing key reagents, experimental models, software and relevant equipment and including their sources and relevant identifiers) followed by a Methods and Protocols section describing the methods, ideally using a step-by-step protocol format. The aim is to facilitate adoption of the methodologies across labs. Please download and fill our Reagents and Tools Table template (.docx), which you can find in our author guidelines: https://www.embopress.org/page/journal/14693178/authorguide#structuredmethods.
When submitting your revised manuscript, please do not include the Reagents and Tools Table in the Methods section of the manuscript but upload it as a separate file choosing the file type "Reagent Table".
An example of a Method paper with Structured Methods can be found here:
https://www.embopress.org/doi/10.15252/msb.20178071

7) Please note that all corresponding authors are required to supply an ORCID ID for their name upon submission of a revised manuscript.

8) It is mandatory to include a 'Data Availability' section after the Materials and Methods. Before submitting your revision, primary

datasets produced in this study need to be deposited in an appropriate public database, and the accession numbers and database listed under 'Data Availability'. Please remember to provide a reviewer password if the datasets are not yet public (see https://www.embopress.org/page/journal/17574684/authorguide#dataavailability).

In case you have no data that requires deposition in a public database, please state so in this section. Note that the Data Availability Section is restricted to new primary data that are part of this study.

9) For data quantification: please specify the name of the statistical test used to generate error bars and P values, the number (n) of independent experiments (specify technical or biological replicates) underlying each data point and the test used to calculate p-values in each figure legend. The figure legends should contain a basic description of n, P and the test applied. Graphs must include a description of the bars and the error bars (s.d., s.e.m.). Please provide exact p values.

10) Our journal encourages inclusion of *data citations in the reference list* to directly cite datasets that were re-used and obtained from public databases. Data citations in the article text are distinct from normal bibliographical citations and should directly link to the database records from which the data can be accessed. In the main text, data citations are formatted as follows: "Data ref: Smith et al, 2001" or "Data ref: NCBI Sequence Read Archive PRJNA342805, 2017". In the Reference list, data citations must be labeled with "[DATASET]". A data reference must provide the database name, accession number/identifiers and a resolvable link to the landing page from which the data can be accessed at the end of the reference. Further instructions are available at .

11) We replaced Supplementary Information with Expanded View (EV) Figures and Tables that are collapsible/expandable online. EV Figures should be cited as 'Figure EV1, Figure EV2" etc... in the text and their respective legends should be included in the main text after the legends of regular figures.
- For the figures that you do NOT wish to display as Expanded View figures, they should be bundled together with their legends in a single PDF file called *Appendix*, which should start with a short Table of Content. Appendix figures should be referred to in the main text as: "Appendix Figure S1, Appendix Figure S2" etc.
- Additional Tables/Datasets should be labeled and referred to as Table EV1, Dataset EV1, etc. Legends have to be provided in a separate tab in case of .xls files. Alternatively, the legend can be supplied as a separate text file (README) and zipped together with the Table/Dataset file.
See detailed instructions here:

12) The paper explained: EMBO Molecular Medicine articles are accompanied by a summary of the articles to emphasize the major findings in the paper and their medical implications for the non-specialist reader. Please provide a draft summary of your article highlighting
- the medical issue you are addressing,
- the results obtained and
- their clinical impact.
This may be edited to ensure that readers understand the significance and context of the research. Please refer to any of our published articles for an example.

13) Author contributions: CRediT has replaced the traditional author contributions section because it offers a systematic machine readable author contributions format that allows for more effective research assessment. Please remove the Authors Contributions from the manuscript and use the free text boxes beneath each contributing author's name in our system to add specific details on the author's contribution. More information is available in our guide to authors.

14) Disclosure statement and competing interests: We updated our journal's competing interests policy in January 2022 and request authors to consider both actual and perceived competing interests. Please review the policy https://www.embopress.org/competing-interests and update your competing interests if necessary.

15) Every published paper now includes a 'Synopsis' to further enhance discoverability. Synopses are displayed on the journal webpage and are freely accessible to all readers. They include a short stand first (maximum of 300 characters, including space) as well as 2-5 one-sentences bullet points that summarizes the paper. Please write the bullet points to summarize the key NEW findings. They should be designed to be complementary to the abstract - i.e. not repeat the same text. We encourage inclusion of key acronyms and quantitative information (maximum of 30 words / bullet point). Please use the passive voice. Please attach these in a separate file or send them by email, we will incorporate them accordingly.

Please also suggest a visual abstract to illustrate your article as a PNG file 550 px wide x 300-600 px high. A cropped portion of this image will serve as thumbnail for the table of content on our webpage.

16) As part of the EMBO Publications transparent editorial process initiative (see our Editorial at http://embomolmed.embopress.org/content/2/9/329), EMBO Molecular Medicine will publish online a Review Process File (RPF) to accompany accepted manuscripts.
In the event of acceptance, this file will be published in conjunction with your paper and will include the anonymous referee

reports, your point-by-point response and all pertinent correspondence relating to the manuscript. Let us know whether you agree with the publication of the RPF and as here, if you want to remove or not any figures from it prior to publication. Please note that the Authors checklist will be published at the end of the RPF.

EMBO Molecular Medicine has a "scooping protection" policy, whereby similar findings that are published by others during review or revision are not a criterion for rejection. Should you decide to submit a revised version, I do ask that you get in touch after three months if you have not completed it, to update us on the status.

I look forward to receiving your revised manuscript.

Yours sincerely,

Lise Roth

Lise Roth, PhD
Senior Editor
EMBO Molecular Medicine

***** Reviewer's comments *****

Referee #1 (Remarks for Author):

The manuscript describes a quasi-mesenchymal stem-like population that have cilia and demonstrates the implication of these cells in mediating chemoresistance. The authors also identify a novel small molecule that targets the quasi-mesenchymal ciliated cells and resensitizes the chemo-resistant sub population by inhibiting ciliogenesis. Overall, the manuscript is well-written and contributes novel insights.

Here are some of the questions that need to be addressed:

1. The definition of a "quasi-mesenchymal" state seems to change across the manuscript. In the introduction, it refers to Ecad+ Vim+ cells that (may) possess stemness and cilia. Later, the authors state that ciliogenesis may promote a "quasi-mesenchymal" cell state. Finally, in cell line experiments shEcadh HMLER cells are called "quasi-mesenchymal". A clear definition of the quasi-mesenchymal cell state, and how it differs from a hybrid state, or the ciliated sub population would help make the manuscript easier to follow.

2. There is growing literature connecting hybrid E/M or quasi-mesenchymal states to resistance to different therapies. The authors should discuss their results in the context of these papers.
https://www.frontiersin.org/journals/immunology/articles/10.3389/fimmu.2021.797261/full
https://pubmed.ncbi.nlm.nih.gov/33328216/
https://www.sciencedirect.com/science/article/pii/S1534580721008911

3. In figure 2E, F and G, it shows that the % of cluster 2 cell population , that has both EMT and ciliogenesis upregulation is higher in drug treated populations [not sure if they can be referred to as chemoresistant population]. This data could be better represented by depicting the % of cells showing high enrichment of EMT and ciliogenesis in the drug treated population.

4. Figure 2 H-K talks about drug-induced plasticity rather than selection of the ciliated sub population. The evidence for this seems to be too weak to support this claim. Two reasons for this are : 1) There is no or weak evidence to link the loss of Chr 11 amplification to appearance of the quasi-mesenchymal phenotype and 2) The loss of Amplification of Chr 11 and the upregulation of EMT and ciliogenesis is shown at a population level, rather than at a single cell level. Additionally, it is not very reliable to infer cell state switching with pre/ post treatment data, where cell lineages are not tracked, or there is no live-cell imaging data.

Referee #2 (Comments on Novelty/Model System for Author):

The manuscript entitled "EMT-ciliary signalling in quasi-mesenchymal-stem-like cells drives therapeutic resistance and is a druggable vulnerability in triple-negative breast cancer" by Tessier et al. focuses on the role of EMT in the therapeutic responses in TNBC. In a model of patient-derived organoids from TNBC samples the authors find that cells in quasy-mesenchymal stem cell-like state display primary cilia and present selective survival to chemotherapy. Further, the authors develop a family of small molecules specifically able to target ciliogenesis and specifically supress chemoresistance in these cells. The main conclusion of this study is that an EMT-ciliary signalling axis induces chemoresistance in quasi-mesenchymal stem-like cells to help chemotherapy evasion and represents an interesting druggable target in TNBC.
Overall, this is a clear and well-written manuscript, bringing novel concepts and understanding in TNBC therapeutic resistance. I only have the following minor points that I would like to bring up for discussion that could further enrich the manuscript.
1. The authors demonstrate a "significant phenotypic heterogeneity at the level of both EMT state and primary ciliogenesis". As stated in Materials and Methods section, "organoids were analysed between passage 0 and passage 7. Did the authors observe any evolution of this heterogeneity along the passages, and is there a selection for a certain phenotype?
2. Among the genes that were found the marker genes of each cluster (Fig 1N) and the marker genes of each cluster in control, doxorubicin and taxol-treated PDOs (Fig 2D)it looks like both MYLK and TPM2 are found overexpressed, and are the only one commonly overexpressed between the two clusterings. Since both are involved in EMT, migration SASP production..., do the authors observe a change in expression of these genes after treatment of PDOS compared to controls?

Response to referees - Ms. No. EMM-2025-21514

We are grateful to the referees for their enthusiasm for our findings, and also their questions, which we found very insightful. We have made revisions to the manuscript to address these comments, including modifications to the text and the addition of a number of new analyses.

We believe that the edits in the text and the added data have strengthened the manuscript and we thank the referees again for their input. Changes to the manuscript are marked as highlighted in the revised version.

Below we give point-by-point answers to the questions. The referees' comments are shown in full in **bold**. Our responses are in regular text with quotes from the edited manuscript shown in *italics*.

Referee #1:

The manuscript describes a quasi-mesenchymal stem-like population that have cilia and demonstrates the implication of these cells in mediating chemoresistance. The authors also identify a novel small molecule that targets the quasi-mesenchymal ciliated cells and resensitizes the chemo-resistant sub population by inhibiting ciliogenesis. Overall, the manuscript is well-written and contributes novel insights.

Here are some of the questions that need to be addressed:

1. The definition of a "quasi-mesenchymal" state seems to change across the manuscript. In the introduction, it refers to Ecad+ Vim+ cells that (may) possess stemness and cilia. Later, the authors state that ciliogenesis may promote a "quasi-mesenchymal" cell state. Finally, in cell line experiments shEcadh HMLER cells are called "quasi-mesenchymal". A clear definition of the quasi-mesenchymal cell state, and how it differs from a hybrid state, or the ciliated sub population would help make the manuscript easier to follow.

We thank the referee for raising this issue. We apologize for not explaining this better in the prior version of the manuscript. We have now edited the text to provide a more detailed description of hybrid E/M states, quasi-mesenchymal states, and a specific discussion on where the quasi-mesenchymal ciliated stem-like state lie along the EMT spectrum. Significant modifications are as follows:

Page 2, paragraph 1: "Tumor cells that activate this cell plasticity program can transit in successive intermediate epithelial-mesenchymal (E/M) states before reaching a fully elongated mesenchymal morphology, an endpoint that is rarely seen in spontaneously arising tumors (Grasset et al, 2022; Gupta et al, 2019; Pastushenko & Blanpain, 2019). The distinct phenotypic intermediate E/M states arrayed between the two E-M extreme phenotypes are also referred to as quasi-mesenchymal states (Yang et al, 2020). Quasi-mesenchymal states encompass at least three distinct phenotypic states, including early hybrid, hybrid, and late hybrid, with different functional properties (Huang et al., 2013; Nieto et al., 2016; Pastushenko & Blanpain, 2019; Wilson et al, 2020; Yu et al, 2013)."

Page 3, paragraph 2: "In the present work we examine human TNBC, and show that cancer cells that have activated EMT and reside in a quasi-mesenchymal ciliated stem-like state represent a subset of late hybrid E/M cells that bear a primary cilium."

Page 4, paragraph 2: "Collectively, our findings establish that the subset of hybrid E/M cancer cells that reside in the most mesenchymal-like state, and display a stem-like phenotype, represents a subpopulation of late hybrid E/M stem-like cells that assemble primary cilia in human TNBCs. From here on, we refer to these cells as quasi-mesenchymal ciliated stem-like cells."

*Page 7 paragraph 1: "*Of note, shEcad cells resided in a more advanced quasi-mesenchymal state along the EMT spectrum in comparison to the quasi-mesenchymal ciliated stem-like cells of PDOs. *However, we demonstrated that quasi-mesenchymal HMLER shEcad cells also display increased levels of primary cilia, as by Arl13b immunostaining ($p \leq 0.001$, Fig. 4B), enabling analysis of the role of primary cilia in cells that acquire a quasi-mesenchymal phenotype upon EMT in this model."*

Page 9 paragraph 1: "Our TNBC PDOs faithfully recapitulate EMT-associated inter- and intratumor phenotypic heterogeneity of cancer cells. Notably, they are comprised of cells residing in multiple quasi-mesenchymal states, enabling investigation on the role of these distinct intermediate EMT states in response to therapy. We demonstrate that a subset of late hybrid E/M cells, bear primary cilia, display a stem-like phenotype, and operate as therapy resistant cells in the analyzed TNBC samples. These data illustrate the value of PDOs for functional studies on the causes and consequences of EMT activation in tumors using patient samples, and for the development of new pharmacological strategies targeting these plasticity programs to counteract therapeutic resistance in human cancers."

2. There is growing literature connecting hybrid E/M or quasi-mesenchymal states to resistance to different therapies. The authors should discuss their results in the context of these papers.

https://www.frontiersin.org/journals/immunology/articles/10.3389/fimmu.2021.797261/fulf;https://pubmed.ncbi.nlm.nih.gov/33328216/;https://www.sciencedirect.com/science/article/pii/S1534580721008911

We have now expanded the introduction to provide additional insights into the quasi-mesenchymal states in therapeutic resistance on page 2, as follows:

"Progress in understanding EMT-induced therapeutic resistance have been possible with the help of mouse tumor models and cancer cell lines in vitro (Shibue & Weinberg, 2017). Emerging studies indicate that neoplastic cells in distinct quasi-mesenchymal states operate as chemotherapy- and immune-checkpoint blockade (ICB)-resistant cells (Dongre et al., 2021; Luond et al, 2021; Sahoo et al, 2021). However, there is still limited understanding of which intermediate EMT states together with their related signaling circuitries mediate therapeutic resistance in human cancers."

3. In figure 2E, F and G, it shows that the % of cluster 2 cell population , that has both EMT and ciliogenesis upregulation is higher in drug treated populations [not sure if they can be referred to as chemoresistant population]. This data could be better represented by depicting the % of cells showing high enrichment of EMT and ciliogenesis in the drug treated population.

We fully agree with the referee that adding data on the enrichment of cells co-expressing high levels of EMT and ciliogenesis programs upon chemotherapy could reinforce the manuscript. To determine the proportion of cells displaying this hallmark after distinct treatments, we plotted cells dissociated from control PDOs and PDOs post-chemotherapy on a graph enabling their separation based on expression of the hallmark EMT signature (x axis, EMT-hallmark score) and of the ciliogenesis signature (y axis, CilioUp score). A population of cells co-expressing both signatures at high levels was identified in each sample and was here referred to as $EMT^{high}/CilioUp^{High}$ cells. We found that the proportion of $EMT^{high}/CilioUp^{High}$ cells were markedly enriched in the drug treated samples in comparison to the control sample (3.23-fold and 4.58-fold enrichment for doxorubicin- and taxol-treated samples, respectively). These data further confirms that cells in the quasi-mesenchymal ciliated state are enriched after chemotherapy. We have added the data in Fig. 2H-I and believe that this analysis strengthens our conclusion. We are grateful to the referee for this suggestion. We have modified the text to discuss this result on page 5, as follows:

"Additionally, we examined the percentage of cells co-expressing high levels of EMT and ciliogenesis transcriptional programs in the distinct PDO samples. We found higher proportions of these cells in post-chemotherapy PDOs, compared to control PDOs (Fig. 2H-I). Altogether, our data establish that chemotherapy results in the selective survival of quasi-mesenchymal ciliated stem-like cells."

4. Figure 2 H-K talks about drug-induced plasticity rather than selection of the ciliated sub population. The evidence for this seems to be too weak to support this claim. Two reasons for this are : 1) There is no or weak evidence to link the loss of Chr 11 amplification to appearance of the quasi-mesenchymal phenotype and 2) The loss of Amplification of Chr 11 and the upregulation of EMT and ciliogenesis is shown at a population level, rather than at a single cell level. Additionally, it is not very reliable to infer cell state switching with pre/ post treatment data, where cell lineages are not tracked, or there is no live-cell imaging data.

We thank the reviewer for raising this issue. We certainly did not wish to exclude that selection of cells pre-existing in the quasi-mesenchymal ciliated state before treatment contribute to development of this cell population in post-chemotherapy PDOs. We wanted to emphasize that both selection of cells that resided in this state before therapy and selection of cells that acquired and progressed in this state as a response to treatment are two potential mechanisms. Each of these two non-mutually exclusive possibilities rely on selection of cells that upregulate EMT and ciliogenesis programs. We realize that we failed to clearly explain this in the prior version of the manuscript. Based on the referee's comment on the weakness of the chr11 data and on the suboptimal pseudotime analysis, we have now revised the manner to address this question in the manuscript with new data and text modifications to offer a more balanced interpretation. The data addressing this question are

now presented in Fig. EV 3A-D. We replaced the chr11 data by newly generated data and we modified the text on page 5, as follows:

"We wondered whether development of the quasi-mesenchymal ciliated stem-like cells in post-chemotherapy PDOs reflects selective survival of cells that acquired this state as a result of upregulation of EMT and ciliogenesis programs before treatment and/or as a response to the drugs. Using pseudotime analysis, we found a trajectory supporting the notion that cells of the quasi-mesenchymal ciliated stem-like cluster arise from cells of the non-quasi-mesenchymal ciliated stem-like clusters both in control and post-chemotherapy PDOs (Fig. EV 3A-C). Progression of single cells along the pseudotime trajectory significantly correlates with their progressive expression of EMT and ciliogenesis programs both in control and post-chemotherapy samples (Fig. EV 3B-C). Interestingly, we found that the amplitude of the correlation is higher in the post-chemotherapy samples, compared to the control (Fig. EV 3B-C). Additionally, we found that some quasi-mesenchymal ciliated cells of cluster 2 which belong to post-chemotherapy PDOs express higher levels of EMT and ciliogenesis programs in comparison to the ones belonging to control PDOs (Fig. EV 3B-D). Altogether, these data suggest that development of the quasi-mesenchymal ciliated stem-like cells in post-chemotherapy PDOs can both reflect selection of cells that acquired this state before treatment and of cells that acquired and progressed in this state as a response to the drugs."

Referee #2:

The manuscript entitled "EMT-ciliary signalling in quasi-mesenchymal-stem-like cells drives therapeutic resistance and is a druggable vulnerability in triple-negative breast cancer" by Tessier et al. focuses on the role of EMT in the therapeutic responses in TNBC. In a model of patient-derived organoids from TNBC samples the authors find that cells in quasy-mesenchymal stem cell-like state display primary cilia and present selective survival to chemotherapy. Further, the authors develop a family of small molecules specifically able to target ciliogenesis and specifically supress chemoresistance in these cells. The main conclusion of this study is that an EMT-ciliary signalling axis induces chemoresistance in quasi-mesenchymal stem-like cells to help chemotherapy evasion and represents an interesting druggable target in TNBC.

Overall, this is a clear and well-written manuscript, bringing novel concepts and understanding in TNBC therapeutic resistance. I only have the following minor points that I would like to bring up for discussion that could further enrich the manuscript.

1. The authors demonstrate a "significant phenotypic heterogeneity at the level of both EMT state and primary ciliogenesis". As stated in Materials and Methods section, "organoids were analysed between passage 0 and passage 7. Did the authors observe any evolution of this heterogeneity along the passages, and is there a selection for a certain phenotype?

We thank the reviewer for raising this important question. We worked on organoids collected at different passages because of the difference in PDO growth dynamics between distinct patient samples. We analyzed organoids at early passages for some samples that we could not maintain in culture in the long term. Our decision to work on PDOs collected from

passage 0 to 7 was based on these parameters and not on the evolution of phenotypic heterogeneity of cells forming the PDOs along the passages.

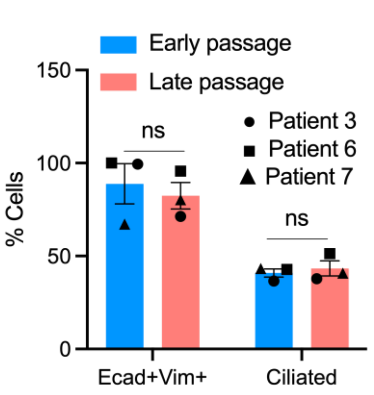

Figure 1: Evolution of phenotypic heterogeneity at the level of EMT and primary ciliogenesis in PDOs. The percentage of Ecad+Vim+ cells and of ciliated cells in PDOs from 3 distinct patient samples at early and late passages was determined using immunostaining for Ecad, Vim and Arl13b. Early passage: < P5 and late passage: > P5. 3-14 PDOs per patient sample per passage. 3 distinct patient samples. ns = not significant.

However, to address the interesting question raised by the Referee, we have now examined the percentage of Ecad+/Vim+ cells and the percentage of ciliated cells in PDOs derived from three distinct patient samples at successive passages. We found no significant difference in the percentage of Ecad+/Vim+ cells ($p = 0.65$) and of ciliated cells ($p = 0.62$) forming PDOs analyzed at distinct passages. We decided not to include these data in the manuscript but have included the data here (Figure 1).

However, we agree with the Referee that providing additional information on the reason why we worked on organoids collected from these passages, in the text, could enrich the manuscript. Accordingly, we modified the methods section on page 10 as follows:

"PDOs were collected at distinct passages (between 0 and 7) because of the difference in the organoid growth dynamics between distinct patient samples. The phenotypic appearance of PDOs over these early successive passages remained similar for each patient sample".

2. Among the genes that were found the marker genes of each cluster (Fig 1N) and the marker genes of each cluster in control, doxorubicin and taxol-treated PDOs (Fig 2D)it looks like both MYLK and TPM2 are found overexpressed, and are the only one commonly overexpressed between the two clusterings. Since both are involved in EMT, migration SASP production..., do the authors observe a change in expression of these genes after treatment of PDOS compared to controls?

We thank the reviewer for raising this other important question. We have now examined the expression of *MYLK* and *TPM2* in control and post-chemotherapy samples. We found higher overall expression of the genes in post-chemotherapy PDOs, compared to control PDOs, reflecting the enrichment of cells expressing high levels of *MYLK* and *TPM2*. These data strengthen our conclusion that post-chemotherapy samples are enriched for cells that reside in the quasi-mesenchymal ciliated stem-like state. We have included new data on *MYLK* and *TPM2* expression in Fig. EV 2C, D, G, H and have modified the text to discuss this on page 4 and 5, as follows:

Page 4, paragraph 3:

"We also conducted scRNAseq analysis of cells dissociated from control or post-chemotherapy PDOs. Unsupervised clustering identified five distinct cell states (clusters 0-4, Fig. 2B-D, Table 4). Cells of cluster 2 and 4 expressed high levels of MYLK and TPM2 marker genes of the quasi-mesenchymal ciliated stem-like state (Fig. 2D, Fig. EV 2C-D), and the highest levels of the EMT, ciliogenesis, and stem cell transcriptional programs ($p \leq 0.0001$,

Fig. 2F-G, Fig. EV 2E-F, Table 5). These data indicate that these cells are in the quasi-mesenchymal ciliated stem-like state."

Page 4, paragraph 4:

"In contrast, the post-chemotherapy PDOs showed reduction in the levels of cluster 1 and 3 cells and increases in the levels of cluster 2 and 4 cells (Fig. 2E), supporting the notion that quasi-mesenchymal ciliated stem-like cells are enriched in post-chemotherapy PDOs. In agreement with this finding, we detected that the proportion of cells expressing high levels of the quasi-mesenchymal ciliated stem-like cell markers MYLK and TPM2 are enriched in post-chemotherapy PDOs (Fig. EV 2G-H). Additionally, we examined the percentage of cells co-expressing high levels of EMT and ciliogenesis transcriptional programs in the distinct PDO samples. We found higher proportions of these cells in post-chemotherapy PDOs, compared to control PDOs (Fig. 2H-I). Altogether, our data establish that chemotherapy results in the selective survival of quasi-mesenchymal ciliated stem-like cells."

27th Jun 2025

Dear Dr. Guen,

Thank you for submitting your revised study. Referees #1 and #2 reviewed your revised manuscript and as you will see below, they are satisfied with the revisions. I will therefore be able to accept your manuscript once the following editorial concerns are addressed:

1/ Manuscript text:
- Please accept previous changes, and only keep in track changes mode any new modification in the text.
- Please provide up to 5 keywords.
- Methods:
o Human samples: Include a statement confirming that informed consent was obtained from all subjects and that the experiments conformed to the principles set out in the WMA Declaration of Helsinki and the Department of Health and Human Services Belmont Report.
o Human research participants: If collected and within the bounds of privacy constraints, report on age, sex and gender or ethnicity for all study participants.
o For all cell material: Report if the cell lines were recently authenticated and tested for mycoplasma contamination.
o Statistics: please provide a statement on inclusion/exclusion criteria, and randomization
- Data availability: thank you for providing reviewer token. Please also provide a URL, and note that the dataset must be publicy available before acceptance of the manuscript. Kindly note that this section is restricted to new primary data that are part of this study.
- Our journal encourages inclusion of *data citations in the reference list* to directly cite datasets that were re-used and obtained from public databases. Data citations in the article text are distinct from normal bibliographical citations and should directly link to the database records from which the data can be accessed. In the main text, data citations are formatted as follows: "Data ref: Smith et al, 2001" or "Data ref: NCBI Sequence Read Archive PRJNA342805, 2017". In the Reference list, data citations must be labeled with "[DATASET]". A data reference must provide the database name, accession number/identifiers and a resolvable link to the landing page from which the data can be accessed at the end of the reference.
- Acknowledgements: please add project numbers where available.
- The figure legends should be divided as follows: main figure legends, followed by the EV figure legends, under the heading Expanded View Figure Legends

2/ Figures:
- Tables 1 - 6 should be renamed Dataset EV1 - Dataset EV6. Each dataset needs its legend removed from the manuscript text and added to the corresponding file, in a separate tab/worksheet.
- Tables 7 - 10 should be renamed Table EV1 - Table EV4. Each table should have its legend added to the top of the page.
- Each movie file will need a legend added to a simple readme text file and placed in a folder that should be uploaded in ZIP format.
- Figures should be called out in chronoligical order in the manuscript text (currently, Figure EV5 is called out before Figure EV2).
- Please address the queries from our data editors in the figure legends:
1. Please define the annotated p values ****/***/**/* as well as provide the exact p-values for the same in the legends of figures 1P, R; 2A, F, G; EV1 G, I as appropriate.
2. Please note that the exact p values are not provided in the legends of figures 1D, H, J, L; 3C, G, I; 4B, C, E, F; 5G, L; EV5 A, EV5 B
3. Please indicate the statistical test used for data analysis in the legends of figures 1D, H, J, L, P, R; 2A, F, G; 3C, G, I; 4B, C, E, F; 5G, L; EV1 G, I; EV3 B, C; EV4 A
4. Please note that the box plots need to be defined in terms of minima, maxima, centre, bounds of box and whiskers, and percentile in the legends of figures 1P, R; 2F, G; 5G, I; EV1 G, I; EV2 C, D, E, F, EV5 B
5. Please note that information related to n is missing in the legends of figures 1C, P, R; 2F, G; 3E, I; 4E, F; 5G, I, J, L; EV1 G, I; EV2 C, D, E, F, G; EV3 D, EV4 B, EV5 B
6. Please note that the error bars are not defined in the legends of figures 3I, 4E, F; 5J

3/ Thank you for providing Source Data. Please upload them as one ZIPed file per figure.

4/ Please provide a complete author checklist, which you can download from our author guidelines (https://www.embopress.org/page/journal/17574684/authorguide#submissionofrevisions). Please insert information in the checklist that is also reflected in the manuscript. The completed author checklist will also be part of the RPF.

5/ I introduced minor edits in your synopsis text, please let me know if you agree or amend as you see fit:
"Cells that activate an epithelial-mesenchymal transition (EMT) program to acquire a quasi-mesenchymal ciliated stem-like state

were shown to mediate therapeutic resistance in human triple-negative breast cancer (TNBC).
o Quasi-mesenchymal ciliated stem-like cells represent a subset of late hybrid epithelial-mesenchymal cells.
o EMT-induced primary cilia mediate chemoresistance of quasi-mesenchymal ciliated stem-like cells.
o The small molecule Nao-3 represses primary ciliogenesis and induces the death of quasi-mesenchymal ciliated stem-like cells."

Thank you for providing a nice visual abstract. Please resize it to a jpeg/TIFF/png file 550 px wide x 300-600 px high. A cropped portion of this image will serve as thumbnail for the table of content on our webpage.

6/ As part of the EMBO Publications transparent editorial process initiative (see our Editorial at http://embomolmed.embopress.org/content/2/9/329), EMBO Molecular Medicine will publish online a Review Process File (RPF) to accompany accepted manuscripts.
This file will be published in conjunction with your paper and will include the anonymous referee reports, your point-by-point response and all pertinent correspondence relating to the manuscript. Let us know whether you agree with the publication of the RPF and as here, if you want to remove or not any figures from it prior to publication.
Please note that the Authors checklist will be published at the end of the RPF.

I look forward to receiving your revised manuscript.

Yours sincerely,

Lise Roth

Lise Roth, PhD
Senior Editor
EMBO Molecular Medicine

***** Reviewer's comments *****

Referee #1 (Remarks for Author):

suitable for publication.

Referee #2 (Remarks for Author):

Many thanks for the review. The authors have sufficiently addressed all of my concerns and significantly improved the manuscript in this revision.

The authors addressed the remaining editorial issues.

9th Jul 2025

Dear Dr. Guen,

Thank you for submitting your revised files. I am pleased to inform you that your manuscript is accepted for publication and is now being sent to our publisher to be included in the next available issue of EMBO Molecular Medicine.

Your manuscript will be processed for publication by EMBO Press. It will be copy edited and you will receive page proofs prior to publication. Please note that you will be contacted by Springer Nature Author Services to complete licensing and payment information.

You may qualify for financial assistance for your publication charges - either via a Springer Nature fully open access agreement or an EMBO initiative. Check your eligibility: https://www.embopress.org/page/journal/17574684/authorguide#chargesguide

Should you be planning a Press Release on your article, please get in contact with embo_production@springernature.com as early as possible in order to coordinate publication and release dates.

If you have any questions, please do not hesitate to contact the Editorial Office. Thank you for your contribution to EMBO Molecular Medicine.

Yours sincerely,

Lise Roth

Lise Roth, Ph.D
Senior Editor
EMBO Molecular Medicine

>>> Please note that it is EMBO Molecular Medicine policy for the transcript of the editorial process (containing referee reports and your response letter) to be published as an online supplement to each paper. If you do NOT want this, you will need to inform the Editorial Office via email immediately. More information is available here: https://www.embopress.org/transparent-process#Review_Process

EMBO Press Author Checklist

Corresponding Author Name: Vincent GUEN
Journal Submitted to: EMBO Molecular Medicine
Manuscript Number: EMM-2025-21514

USEFUL LINKS FOR COMPLETING THIS FORM
The EMBO Journal - Author Guidelines
EMBO Reports - Author Guidelines
Molecular Systems Biology - Author Guidelines
EMBO Molecular Medicine - Author Guidelines

Reporting Checklist for Life Science Articles (updated January
This checklist is adapted from Materials Design Analysis Reporting (MDAR) Checklist for Authors. MDAR establishes a minimum set of requirements in transparent reporting in the life sciences (see Statement of Task: 10.31222/osf.io/9sm4x). Please follow the journal's guidelines in preparing your
Please note that a copy of this checklist will be published alongside your article.

Abridged guidelines for figures
1. Data
The data shown in figures should satisfy the following conditions:
- the data were obtained and processed according to the field's best practice and are presented to reflect the results of the experiments in an accurate and unbiased manner.
- ideally, figure panels should include only measurements that are directly comparable to each other and obtained with the same assay.
- plots include clearly labeled error bars for independent experiments and sample sizes. Unless justified, error bars should not be shown for technical
- if n<5, the individual data points from each experiment should be plotted. Any statistical test employed should be justified.
- Source Data should be included to report the data underlying figures according to the guidelines set out in the authorship guidelines on Data

2. Captions
Each figure caption should contain the following information, for each panel where they are relevant:
- a specification of the experimental system investigated (eg cell line, species name).
- the assay(s) and method(s) used to carry out the reported observations and measurements.
- an explicit mention of the biological and chemical entity(ies) that are being measured.
- an explicit mention of the biological and chemical entity(ies) that are altered/varied/perturbed in a controlled manner.
- the exact sample size (n) for each experimental group/condition, given as a number, not a range;
- a description of the sample collection allowing the reader to understand whether the samples represent technical or biological replicates (including how many animals, litters, cultures, etc.).
- a statement of how many times the experiment shown was independently replicated in the laboratory.
- definitions of statistical methods and measures:
  - common tests, such as t-test (please specify whether paired vs. unpaired), simple χ2 tests, Wilcoxon and Mann-Whitney tests, can be unambiguously identified by name only, but more complex techniques should be described in the methods section;
  - are tests one-sided or two-sided?
  - are there adjustments for multiple comparisons?
  - exact statistical test results, e.g., P values = x but not P values < x;
  - definition of 'center values' as median or average;
  - definition of error bars as s.d. or s.e.m.

Please complete ALL of the questions below.
Select "Not Applicable" only when the requested information is not relevant for your study.

Materials

Newly Created Materials	Information included in the manuscript?	In which section is the information available? (Reagents and Tools Table, Materials and Methods, Figures, Data Availability Section)
New materials and reagents need to be available; do any restrictions apply?	Yes	Material and Methods, Data Availbability Section

Antibodies	Information included in the manuscript?	In which section is the information available? (Reagents and Tools Table, Materials and Methods, Figures, Data Availability Section)
For **antibodies** provide the following information:<br>- Commercial antibodies: RRID (if possible) or supplier name, catalogue number and or/clone number<br>- Non-commercial: RRID or citation	Yes	Material and Methods, Reagents_Tools_Table

DNA and RNA sequences	Information included in the manuscript?	In which section is the information available? (Reagents and Tools Table, Materials and Methods, Figures, Data Availability Section)
**Short novel DNA or RNA including primers, probes**: provide the sequences.	Yes	Material and Methods

Cell materials	Information included in the manuscript?	In which section is the information available? (Reagents and Tools Table, Materials and Methods, Figures, Data Availability Section)
**Cell lines:** Provide species information, strain. Provide accession number in repository **OR** supplier name, catalog number, clone number, and/**OR** RRID.	Yes	Material and Methods, Reagents_Tools_Table
**Primary cultures:** Provide species, strain, sex of origin, genetic modification status.	Yes	Material and Methods, Reagents_Tools_Table
Report if the cell lines were recently **authenticated** (e.g., by STR profiling) and tested for mycoplasma contamination.	Yes	Material and Methods

Experimental animals	Information included in the manuscript?	In which section is the information available? (Reagents and Tools Table, Materials and Methods, Figures, Data Availability Section)
**Laboratory animals or Model organisms:** Provide species, strain, sex, age, genetic modification status. Provide accession number in repository **OR** supplier name, catalog number, clone number, **OR** RRID.	Not Applicable	
**Animal observed in or captured from the field:** Provide species, sex, and age where possible.	Not Applicable	
Please detail **housing and husbandry conditions**.	Not Applicable	

Plants and microbes	Information included in the manuscript?	In which section is the information available? (Reagents and Tools Table, Materials and Methods, Figures, Data Availability Section)
**Plants:** provide species and strain, ecotype and cultivar where relevant, unique accession number if available, and source (including location for collected wild specimens).	Not Applicable	
**Microbes:** provide species and strain, unique accession number if available, and source.	Not Applicable	

Human research participants	Information included in the manuscript?	In which section is the information available? (Reagents and Tools Table, Materials and Methods, Figures, Data Availability Section)
If collected and within the bounds of privacy constraints report on age, sex and gender or ethnicity for all study participants.	Yes	Material and Methods

Core facilities	Information included in the manuscript?	In which section is the information available? (Reagents and Tools Table, Materials and Methods, Figures, Data Availability Section)
If your work benefited from core facilities, was their service mentioned in the acknowledgments section?	Yes	Ackowledgments

Design

**Study protocol**	**Information included in the manuscript?**	**In which section is the information available?** (Reagents and Tools Table, Materials and Methods, Figures, Data Availability Section)
If study protocol has been **pre-registered, provide DOI in the manuscript**. For clinical trials, provide the trial registration number **OR** cite DOI.	Yes	Material and Methods
Report the **clinical trial registration number** (at ClinicalTrials.gov or equivalent), where applicable.	Not Applicable	

**Laboratory protocol**	**Information included in the manuscript?**	**In which section is the information available?** (Reagents and Tools Table, Materials and Methods, Figures, Data Availability Section)
Provide DOI OR other citation details if **external detailed step-by-step protocols** are available.	Not Applicable	

**Experimental study design and statistics**	**Information included in the manuscript?**	**In which section is the information available?** (Reagents and Tools Table, Materials and Methods, Figures, Data Availability Section)
Include a statement about **sample size** estimate even if no statistical methods were used.	Yes	Material and Methods
Were any steps taken to minimize the effects of subjective bias when allocating animals/samples to treatment (e.g. **randomization procedure**)? If yes, have they been described?	Yes	Material and Methods
Include a statement about **blinding** even if no blinding was done.	Yes	Material and Methods
Describe **inclusion/exclusion criteria** if samples or animals were excluded from the analysis. Were the criteria pre-established?<br><br>If sample or data points were omitted from analysis, report if this was due to attrition or intentional exclusion and provide justification.	Yes	Material and Methods
For every figure, are **statistical tests** justified as appropriate? Do the data meet the assumptions of the tests (e.g., normal distribution)? Describe any methods used to assess it. Is there an estimate of variation within each group of data? Is the variance similar between the groups that are being statistically compared?	Yes	Material and Methods

**Sample definition and in-laboratory replication**	**Information included in the manuscript?**	**In which section is the information available?** (Reagents and Tools Table, Materials and Methods, Figures, Data Availability Section)
In the figure legends: state number of times the experiment was **replicated** in laboratory.	Yes	Material and Methods, Figures
In the figure legends: define whether data describe **technical or biological replicates**.	Yes	Material and Methods, Figures

Ethics

**Ethics**	**Information included in the manuscript?**	**In which section is the information available?** (Reagents and Tools Table, Materials and Methods, Figures, Data Availability Section)
Studies involving **human participants**: State details of **authority granting ethics approval** (IRB or equivalent committee(s), provide reference number for approval.	Yes	Material and Methods
Studies involving **human participants**: Include a statement confirming that **informed consent** was obtained from all subjects and that the experiments conformed to the principles set out in the WMA Declaration of Helsinki and the Department of Health and Human Services Belmont Report.	Yes	Material and Methods
Studies involving **human participants: For** publication of **patient photos**, include a statement confirming that consent to publish was obtained.	Not Applicable	
Studies involving experimental **animals**: State details of **authority granting ethics approval** (IRB or equivalent committee(s), provide reference number for approval. Include a statement of compliance with ethical regulations.	Not Applicable	
Studies involving **specimen and field samples:** State if relevant **permits** obtained, provide details of authority approving study; if none were required, explain why.	Not Applicable	

**Dual Use Research of Concern (DURC)**	**Information included in the manuscript?**	**In which section is the information available?** (Reagents and Tools Table, Materials and Methods, Figures, Data Availability Section)
Could your study fall under dual use research restrictions? Please check biosecurity documents and list of **select agents and toxins** (CDC): https://www.selectagents.gov/sat/list.htm	Not Applicable	
If you used a select agent, is the security level of the lab appropriate and reported in the manuscript?	Not Applicable	
If a study is subject to dual use research of concern regulations, is the name of the **authority granting approval and reference number** for the regulatory approval provided in the manuscript?	Not Applicable	

Reporting

The MDAR framework recommends adoption of discipline-specific guidelines, established and endorsed through community initiatives. Journals have their own policy about requiring specific guidelines and recommendations to complement MDAR.

**Adherence to community standards**	**Information included in the manuscript?**	**In which section is the information available?** (Reagents and Tools Table, Materials and Methods, Figures, Data Availability Section)
State if relevant guidelines or checklists (e.g., **ICMJE, MIBBI, ARRIVE, PRISMA**) have been followed or provided.	Not Applicable	
For **tumor marker prognostic studies,** we recommend that you follow the **REMARK** reporting guidelines (see link list at top right). See author guidelines, under 'Reporting Guidelines'. Please confirm you have followed these guidelines.	Not Applicable	
For **phase II and III randomized controlled trials**, please refer to the **CONSORT** flow diagram (see link list at top right) and submit the CONSORT checklist (see link list at top right) with your submission. See author guidelines, under 'Reporting Guidelines'. Please confirm you have submitted this list.	Not Applicable	

Data Availability

**Data availability**	**Information included in the manuscript?**	**In which section is the information available?** (Reagents and Tools Table, Materials and Methods, Figures, Data Availability Section)
Have **primary datasets** been deposited according to the journal's guidelines (see 'Data Deposition' section) and the respective accession numbers provided in the Data Availability Section?	Yes	Datasets publicly available (https://www.ncbi.nlm.nih.gov/geo/query/acc.cgi?acc=GSE298840)
Were **human clinical and genomic datasets** deposited in a public access-controlled repository in accordance to ethical obligations to the patients and to the applicable consent agreement?	Yes	https://www.ncbi.nlm.nih.gov/geo/query/acc.cgi?acc=GSE298840
Are **computational models** that are central and integral to a study available without restrictions in a machine-readable form? Were the relevant accession numbers or links provided?	Not Applicable	
If publicly available data were reused, provide the respective **data citations in the reference list**.	Yes	References
